# Retro-Cope elimination of cyclic alkynes: reactivity trends and rational design of next-generation bioorthogonal reagents†

**DOI:** 10.1039/d4sc04211e

**Published:** 2024-08-27

**Authors:** Steven E. Beutick, Song Yu, Laura Orian, F. Matthias Bickelhaupt, Trevor A. Hamlin

**Affiliations:** a Department of Chemistry and Pharmaceutical Sciences, Amsterdam Institute of Molecular and Life Sciences (AIMMS), Vrije Universiteit Amsterdam De Boelelaan 1108 Amsterdam 1081 HZ The Netherlands t.a.hamlin@vu.nl; b Dipartimento di Scienze Chimiche, Università degli Studi di Padova Via Marzolo 1 Padova 35129 Italy; c Institute of Molecules and Materials, Radboud University Heyendaalseweg 135 Nijmegen 6525 AJ The Netherlands; d Department of Chemical Sciences, University of Johannesburg Auckland Park Johannesburg 2006 South Africa

## Abstract

The retro-Cope elimination reaction between dimethylhydroxylamine (DMHA) and various cyclic alkynes has been quantum chemically explored using DFT at ZORA-BP86/TZ2P. The purpose of this study is to understand the role of the following three unique activation modes on the overall reactivity, that is (i) additional cycloalkyne predistortion *via* fused cycles, (ii) exocyclic heteroatom substitution on the cycloalkyne, and (iii) endocyclic heteroatom substitution on the cycloalkyne. Trends in reactivity are analyzed and explained by using the activation strain model (ASM) of chemical reactivity. Based on our newly formulated design principles, we constructed *a priori* a suite of novel bioorthogonal reagents that are highly reactive towards the retro-Cope elimination reaction with DMHA. Our findings offer valuable insights into the design principles for highly reactive bioorthogonal reagents in chemical synthesis.

## Abstract 2

Diversify your bonds! The reactivity of preactivated cycloalkynes in the retro-Cope elimination reaction was quantum chemically investigated. Based on our finding, we rationally designed a suite of next-generation reagents.

## Graphical abstract

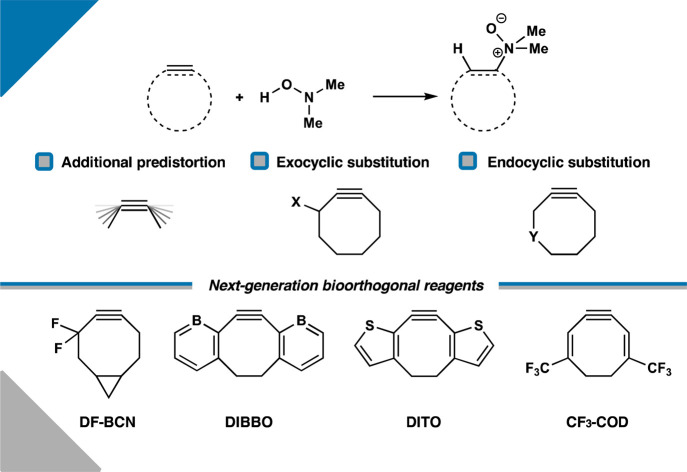

## Introduction

Bioorthogonal chemistry has emerged as a powerful tool for studying and manipulating biological systems.^1^ Bioorthogonal reactions are biocompatible and robust, go with fast reaction rate constants, and do not interfere or react with the native biological environment. Bioorthogonal reagents that meet these requirements enable the probing of biological processes by labeling and imaging target molecules, both *in vitro* and *in vivo*.^2^ Common biorthogonal reactions are the tetrazine-based inverse electron demand Diels–Alder (IED-DA),^3^ traceless Staudinger ligation reactions,^4^ and 1,3-dipolar cycloadditions^5^ such as copper(i)-catalyzed azide–alkyne cycloaddition (CuAAC). Bertozzi and co-workers developed the strain-promoted azide–alkyne cycloaddition reaction (SPAAC) as a selective biorthogonal chemical reaction.^6^ The field continues to flourish with the development of several novel bioorthogonal reactions, including strain-promoted alkyne-nitrone cycloadditions (SPANC),^7^ isonitrile-based [4 + 1] cycloadditions,^8^ and photoactivated tetrazole ligations.^9^

**Scheme 1:**
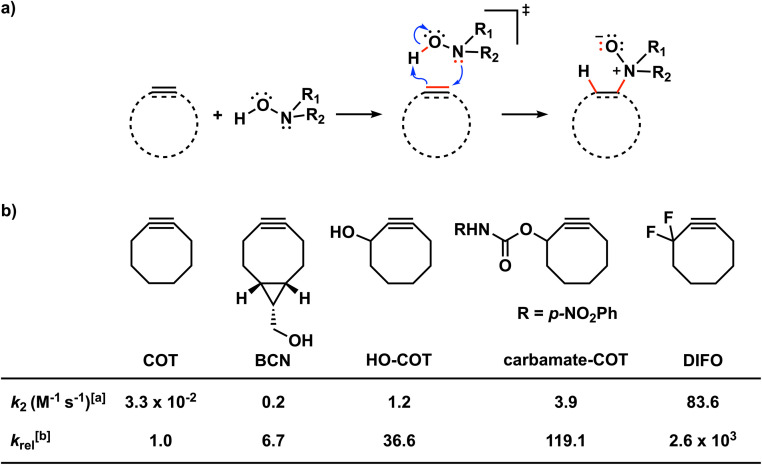
(a) The retro-Cope elimination reactions between cyclic alkynes or alkenes and hydroxylamines. (b) Effects of modifying cyclic octynes on the reactivity in the retro-Cope elimination reaction with diethylhydroxylamine.^10*a*^^*a*^ The second-order rate constants were determined for a 1 : 1 ratio of the reactants in CD_3_CN at room temperature using NMR spectroscopy.^1^^*b*^ The relative second-order rate constants (*k*_rel_) were calculated relative to the parent cyclooctyne, COT (*k*_rel_ = *k*/*k*_COT_).

Recently, Kang *et al.* presented the retro-Cope elimination reaction (hydroamination of cyclic cyclooctyne) between functionalized (cyclic) alkynes and *N*,*N*-dialkylhydroxylamines as a novel bioorthogonal reaction (Scheme 1a).^10^ The calculated Gibbs free energy activation barrier at M06-2X/6-311G(2d,p)//M06-2X/6-31G(d,p) for the parent cyclooctyne was determined to be 18.9 kcal mol^−1^, which is adequate for the reaction to proceed at room temperature. Experimentally, the retro-Cope elimination reaction between the non-substituted cyclooctyne (COT) and hydroxylamine in CD_3_CN at room temperature shows second-order rate constants of 3.25 × 10^−2^ M^−1^ s^−1^, an order of magnitude higher than the strain-promoted azide–alkyne cycloaddition (SPAAC) reaction with benzyl azide.^10*a*,11^ Notably, the retro-Cope elimination reaction is highly regioselective when the COT is substituted with electronegative heteroatoms at the propargylic position. Furthermore, Kang *et al.* showcased the retro-Cope elimination reaction in an *in vitro* study and demonstrated mutual orthogonality in conjunction with the inverse electron demand Diels Alder (IED-DA) reaction between tetrazine and strained alkenes.^10*a*,12^ In doing so, they nicely underscored the potential of this reaction to be applied in mutually orthogonal bioorthogonal chemistry.

**Scheme 2:**
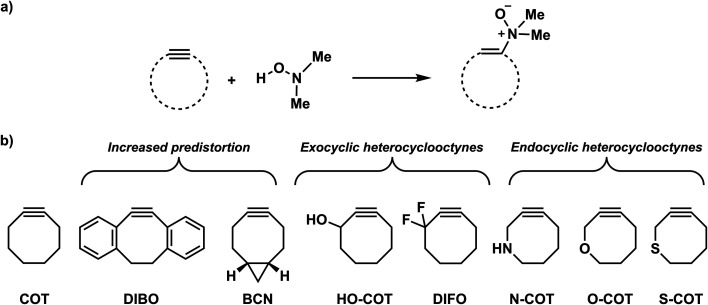
Studied retro-Cope elimination reactions between (substituted) cyclooctynes and dimethylhydroxylamine (DMHA). The set of reactants included in this study consists of the archetypal cyclooctyne COT, cyclooctynes with additional predistortion by rigid cyclic substituents (DIBO, BCN), and with exocyclic heteroatoms (HO-COT, DIFO) and endocyclic heteroatoms (N-COT, O-COT, S-COT).

Decorated cyclooctynes have emerged as functional bioorthogonal reagents in 1,3-dipolar cycloadditions.^5*e*,13^ Similarly, these cyclic alkynes show high reactivity and selectivity towards the retro-Cope elimination reaction.^10*a*^ Kim and co-workers^10*a*^ reported only a minor reactivity enhancement after the introduction of additional strain by three-membered ring fusion in bicyclo[6.1.0]nonyne (BCN), in contrast to a drastic two-order magnitude increase in the second-order rate constants observed for the SPAAC reaction reported by Dommerholt *et al.*^14^ The authors described further enhanced reactivity upon adding electronegative substituents on the exocyclic propargylic position, achieving up to three orders of magnitude increase in the second-order rate constants (Scheme 2). Using the distortion/interaction model, Kang *et al.* show that the predistortion gives rise to the lowering of the activation barrier for the strained cyclooctyne relative to its linear counterpart.^15,16*a*^ Interestingly, upon the addition of electronegative substituents on the propargylic position, they found that the lowering of the activation barrier was not caused by a less favorable interaction term but, rather, driven by the lowering of the strain energy for both fragments. They further attribute this to a shift of the transition state towards the reactants in accordance with the Hammond postulate.

We have computationally investigated the concerted retro-Cope elimination reaction between dimethylhydroxylamine (DMHA) and cyclic alkynes at ZORA-BP86/TZ2P (Scheme 2). Three sets of cyclooctynes were studied. The first set includes cyclic alkynes with increased predistortion due to the introduction of cyclic functionality (DIBO and BCN). The second and third sets consist of cycloalkynes modified at the exocyclic (HO-COT and DIFO) and endocyclic (N-COT, O-COT, and S-COT) propargylic position, respectively. The *anti*-pathways (dimethyl groups and propargylic substituent on opposite sides) and the *syn*-pathways (dimethyl groups and propargylic substituent on the same side) were investigated. To gain insight into the physical factors that control the reactivity, the activation strain model (ASM)^16^ of reactivity in conjunction with the matching energy decomposition analysis (EDA) and quantitative Kohn–Sham molecular orbital (KS-MO)^17^ theory analysis. Based on our found rational design principles, we constructed *a priori* a suite of novel bioorthogonal reagents that are highly reactive towards the retro-Cope elimination reaction with DMHA.

## Computational methods

### Computational details

All calculations were performed with the AMS2021 program (an example input file is provided in the ESI†).^18^ After a conformational search using Grimme's CREST 2.12 (ref. 19) using default settings in the gas phase, all geometries and energies were computed using the generalized gradient approximation (GGA) functional BP86,^20^ and the MOs were expanded using a large uncontracted set of Slater-type orbitals (STO): TZ2P.^21^ Our benchmarks^22*a*,*b*^ and numerous DFT studies^22*c*–*g*^ support the use of this method to compute trends in reactivity for similar reactions. The trends in reactivity remain the same in implicit solvent (COSMO(acetonitrile)-BP86/TZ2P//BP86/TZ2P). Additionally, the trends were successfully reproduced with dispersion corrections (BP86-D3(BJ)/TZ2P//BP86/TZ2P), and at M06-2X/TZ2P//BP86/TZ2P, and DLPNO-CCSD(T)/def2-QZVPP//BP86/TZ2P (Table S3†). The zeroth order approximation (ZORA) accounted for the scalar relativistic effects.^23^ The accuracies of the fit scheme (Zlm fit)^24*a*^ and the integration grid (Becke grid)^24*b*^ were set to VERYGOOD. The convergence criteria for the SCF and geometry optimization procedures were set to 10^−5^ hartree. Frequency calculations were performed to characterize the nature of stationary points where local minima had real frequencies while transition structures had a single imaginary frequency. The steepest descent path from the TS was calculated using the intrinsic reaction coordinate (IRC) method,^25^ which follows the transition vector (the vibrational normal mode associated with the reaction and with a negative force constant) from the transition structure toward the reactants and product and was analyzed with the aid of the PyFrag 2019.^26^ All structures were visualized using CYLview.^27^

### Activation strain model and energy decomposition analysis

Quantitative analyses of the activation barriers associated with the studied reactions were obtained using the activation strain model (ASM) of reactivity.^16^ The PES, that is, Δ*E*(*ζ*), was decomposed into the strain energy, Δ*E*_strain_(*ζ*), and interaction energy, Δ*E*_int_(*ζ*) [eqn (1)]. All energy terms were projected onto the length of the forming C⋯H bond, which undergoes a well-defined change during the reaction. Other reaction coordinates, such as the length of the C⋯N bond or C⋯C stretch, were shown to provide the same general conclusions.^28^

1 Δ*E*(*ζ*) = Δ*E*_strain_(*ζ*) + Δ*E*_int_(*ζ*)

The Δ*E*_strain_(*ζ*) is associated with the rigidity and the structural deformation of the reactants from their equilibrium structure to the geometry they adopt at the coordinate of *ζ* during the reaction. The Δ*E*_int_(*ζ*) is related to the electronic structure of the reactants and their spatial orientation and represents the mutual interactions between the deformed reactants.

To obtain a deeper insight into the physical mechanism behind Δ*E*_int_(*ζ*), we employed our canonical energy decomposition analysis (EDA),^17^ which decomposes the Δ*E*_int_ between the deformed reactants, within the framework of Kohn–Sham MO theory, into three physically meaningful terms [eqn (2)].

2 Δ*E*_int_(*ζ*) = Δ*V*_elstat_(*ζ*) + Δ*E*_Pauli_(*ζ*) + Δ*E*_oi_(*ζ*)

The electrostatic interaction, Δ*V*_elstat_(*ζ*), corresponds to the classical electrostatic interaction between the unperturbed charge distributions of the deformed reactants. The Pauli repulsion, Δ*E*_Pauli_(*ζ*), comprises the Pauli-repulsive orbital interactions between closed-shell orbitals. The term Δ*E*_oi_(*ζ*) represents the stabilizing orbital interactions, such as charge transfer, namely, the interactions between the occupied orbitals of one reactant and the unoccupied orbitals of the other reactant, and polarization, that is, the occupied–unoccupied orbital mixing within one reactant due to the presence of the other reactant.

### Voronoi deformation density analysis

The electron density distribution is analyzed using the Voronoi deformation density (VDD) method^29^ for computing atomic charges. The VDD atomic charge on atom A (*Q*^VDD^_A_) is computed as the (numerical) integral of the deformation density in the volume of the Voronoi cell of atom A (eqn (3)). The Voronoi cell of atom A is defined as the compartment of space bounded by the bond midplanes on and perpendicular to all bond axes between nucleus A and its neighboring nuclei.3
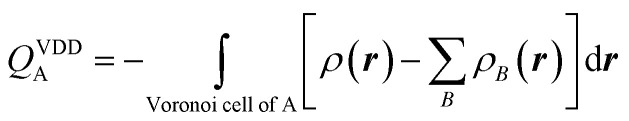


Here, *ρ*(*r*) is the electron density of the molecule, and 
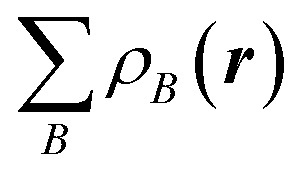
 is the superposition of atomic densities *ρ*_*B*_ of a fictitious promolecule without chemical interactions that is associated with the situation in which all atoms are neutral. The interpretation of the VDD charge *Q*^VDD^_A_ is rather straightforward and transparent: instead of measuring the amount of charge associated with a particular atom A, *Q*^VDD^_A_ directly monitors how much charge flows, due to chemical interactions, out of (*Q*^VDD^_A_ > 0) or into (*Q*^VDD^_A_ < 0) the Voronoi cell of atom A.

## Results and discussion

### Cyclic alkynes

First, we discuss the effect of the bending of the alkyne, or alkyne predistortion, on the retro-Cope elimination reactivity by comparison of the linear 2-butyne and cyclooctyne (COT). The computed electronic activation energies (Δ*E*^‡^) and reaction energies (Δ*E*_rxn_) associated with the retro-Cope elimination reaction between these reactants and dimethylhydroxylamine (DMHA) are provided in Table 1. The electronic activation barrier is significantly lowered from 15.8 kcal mol^−1^ to 4.6 kcal mol^−1^ (Table 1) going from 2-butyne to COT.

**Table 1:** Transition state structures for the retro-Cope elimination reactions, Δ*r* in the transition state (in Å), electronic energies relative to reactants of the reactant complex Δ*E*_RC_ (in kcal mol^−1^), transition state Δ*E*^‡^ (in kcal mol^−1^), product Δ*E*_rxn_ (in kcal mol^−1^), Gibbs free energies of activation Δ*G*^‡^ (in kcal mol^−1^) and the second-order rate constants relative to COT, *k*_rel_, for retro-Cope elimination reactions of 2-butyne and COT with DMHA a

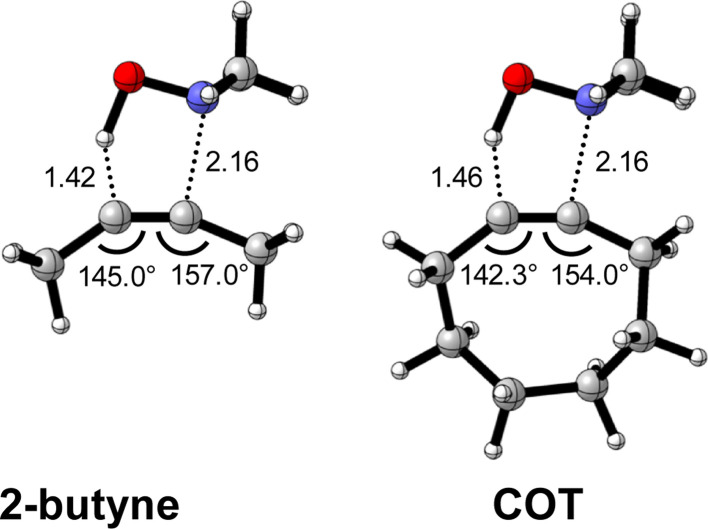		
	2-Butyne	COT
Δ*r*^b^	0.74	0.70
Δ*E*_RC_	−3.0	−3.0
Δ*E*^‡^	15.8	4.6
Δ*E*_rxn_	−6.9	−14.4
Δ*G*^‡^	26.7	15.8
*k*_rel_ c	1.2 × 10^−8^	1.0

a Computed at ZORA-BP86/TZ2P.

b Δ*r* is the difference in length between the forming C⋯N and C⋯H bonds at the transition state.

c The relative second-order rate constants (*k*_rel_) were calculated relative to the COT (*k*/*k*_COT_).

**Fig. 1:**
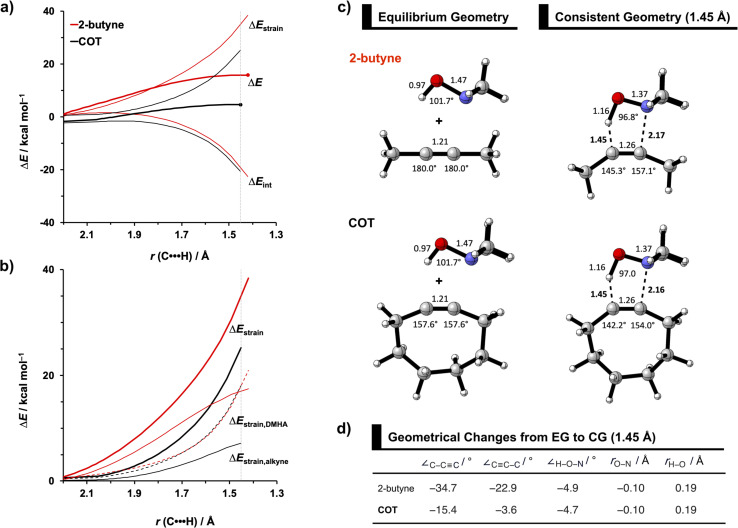
(a) Activation strain model and (b) decomposition of strain energies for the retro-Cope elimination reactions of 2-butyne and COT with DMHA. Transition states are indicated by dots. The energy terms along the IRC are projected on the length of the newly forming C⋯H bond and the vertical dotted line indicates the point at which the length of the C⋯H bond is 1.45 Å. (c) Structural information (in Å and deg.) associated with the retro-Cope elimination reactions of propyne and COT with DMHA along *anti*-pathways and (d) changes in key angles and bond distances from equilibrium geometries to consistent geometries where the lengths of the C⋯H bond are 1.45 Å. All were computed at ZORA-BP86/TZ2P.

To identify the physical factors leading to the enhanced retro-Cope elimination reactivity of the strained alkynes relative to the linear 2-butyne, the activation strain model (ASM) of reactivity is applied (Fig. 1a). We find that predistortion of the alkyne in COT serves to enhance retro-Cope elimination reactivity *via* (1) reducing the activation strain and (2) enhancing the overall interaction energy *via* more stabilizing orbital interactions. Kim *et al.* also observed a notable reduction in the activation strain of the retro-Cope elimination reaction with COT.^10^ Relative to its linear counterpart, the strain in COT is reduced by approximately 10 kcal mol^−1^ at a consistent geometry, where the lengths of the C⋯H bond are 1.45 Å, and the slope of the strain in the alkyne fragment is smaller along the reaction coordinate. By partitioning the strain energy in terms of the two fragments (Fig. 1b), we confirm that the strain is lowered for the COT fragment, due to the alkyne predistortion in the COT reagent. To understand the origin of this effect, further analysis of both equilibrium geometry and consistent geometry (Fig. 1c and d) reveals that the reaction of predistorted alkyne, COT, simply requires less bending of the alkyne to react with DMHA.

In addition, we show how predistortion of COT also acts to enhance the stabilizing interaction energy with DMHA (Fig. 1a). Using our EDA, we could pinpoint the enhanced interaction energy originates from significantly more stabilizing orbital interactions (Fig. S3b†). The predistortion of the alkyne results in a stabilization of the LUMO and a slight destabilization of the HOMO.^30^ The lowering of the LUMO, is caused by the in-phase mixing of the σ* and π* orbitals, resulting in a smaller π*-LUMO_COT_ and HOMO_DMHA_ energy gap.

### Cyclic alkynes with additional predistortion

Next, we compare the reactivity of our parent COT with two popular bioorthogonal cyclic alkynes reagents in 1,3-dipolar cycloaddition reactions^5^ dibenzocyclooctyne (DIBO)^31^ and bicyclononyne (BCN)^14^ that feature additional alkyne predistortion (∠C–C–C) from the presence of fused carbocycles (154.7° and 154.5° relative to 157.6° for COT in the optimized reactants (Fig. S1†)). The computed electronic activation energies (Δ*E*^‡^) and reaction energies (Δ*E*_rxn_) associated with the retro-Cope elimination reaction between these reactants and dimethylhydroxylamine (DMHA) are shown in Table 2. Our calculations show that the activation barrier for the retro-Cope elimination reaction with DMHA decreases from 4.6 to 4.1 to 2.1 kcal mol^−1^, as going from COT to DIBO to BCN.

**Table 2:** Transition state structures for the retro-Cope elimination reactions, Δ*r* in the transition state (in Å), electronic energies relative to reactants of the reactant complex Δ*E*_RC_ (in kcal mol^−1^), transition state Δ*E*^‡^ (in kcal mol^−1^), product Δ*E*_rxn_ (in kcal mol^−1^), Gibbs free energies of activation Δ*G*^‡^ (in kcal mol^−1^) and the second-order rate constants relative to COT, *k*_rel_, for retro-Cope elimination reactions of COT, DIBO, and BCN with DMHA a

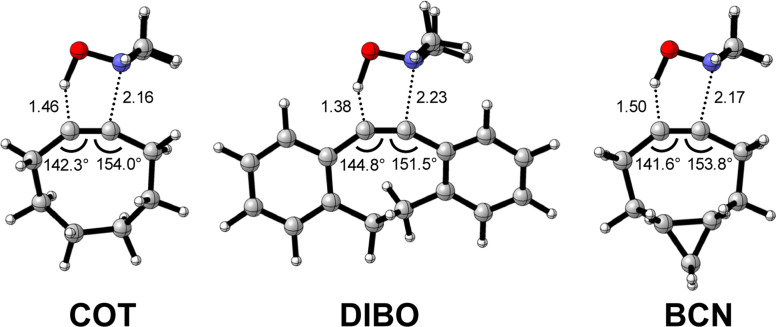			
	COT	DIBO	BCN
Δ*r*^b^	0.70	0.85	0.67
Δ*E*_RC_	−3.0	−2.7	−3.1
Δ*E*^‡^	4.6	4.1	2.1
Δ*E*_rxn_	−14.4	−9.4	−12.7
Δ*G*^‡^	15.8	15.6	13.9
*k*_rel_ c	1.0	1.5	27.0

a Computed at ZORA-BP86/TZ2P.

b Δ*r* is the difference in length between the forming C⋯N and C⋯H bonds at the transition state.

c The relative second-order rate constants (*k*_rel_) were calculated relative to COT (*k*/*k*_COT_).

**Fig. 2:**
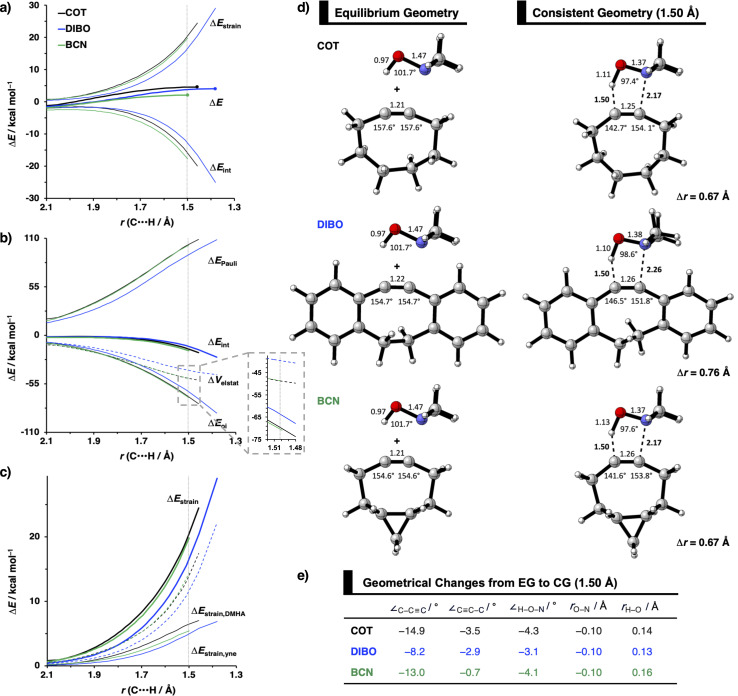
(a) Activation strain diagram, (b) energy decomposition analyses and (c) decomposition of strain energies for the retro-Cope elimination reactions of cyclooctyne (COT), dibenzocyclooctyne (DIBO), and bicyclononyne (BCN) with dimethylhydroxylamine (DMHA) along the *anti*-pathways. Transition states are indicated by dots. The energy terms along the IRC are projected on the length of the newly forming C⋯H bond and the vertical dotted line indicates the point at which the length of the C⋯H bond is 1.50 Å. (d) Structural information and (e) geometrical changes (in Å and deg.) for the retro-Cope elimination reactions of COT, DIBO, and BCN with DMHA along the *anti*-pathways at equilibrium geometries and consistent geometries where the lengths of the C⋯H bond are 1.50 Å.

The physical factors leading to the enhanced retro-Cope elimination reactivity of DIBO and BCN compared to COT are next analyzed using the ASM. In Fig. 2b, the ASM diagram shows that the retro-Cope elimination reaction of DIBO benefits from significantly less destabilizing strain energy (Δ*E*_strain_), as compared to COT. The retro-Cope elimination reaction of BCN with DMHA also benefits from less destabilizing strain but is paralleled by a more stabilizing interaction energy (Δ*E*_int_), as compared to COT (Fig. 2b). Decomposition of the strain energies reveals that DIBO benefits a reduced strain in both the DIBO and DMHA fragments, whereas the reduced strain for BCN at consistent geometry originates solely from the BCN fragment (Fig. 2d). Our structural analyses at the equilibrium geometry show that DIBO and BCN both have a more predistorted backbone than the parent COT (157.6°) in the equilibrium geometry, with internal C–C≡C alkyne bond angles of 154.7° and 154.6°, respectively. This predistortion results in a structurally less distorted alkyne fragment at consistent geometry (Fig. 2d), and, consequently, reduced activation strain. For DIBO, the lowering of the activation strain is more pronounced because also the O⋯H bond breaking distance and compression of the H–O–N angle are less progressed, which causes the strain of the DMHA fragment to be shifted to the right (Fig. 2a). As will be explained in more detail later on, this can be traced back to a more slanted reaction path, that is, the C⋯N bond is formed later than the C⋯H bond for DIBO (Δ*r* = 0.76 Å, where Δ*r* is the difference in length between the C⋯N and C⋯H bonds at the consistent geometry) than for COT (Δ*r* = 0.67 Å).

**Fig. 3:**
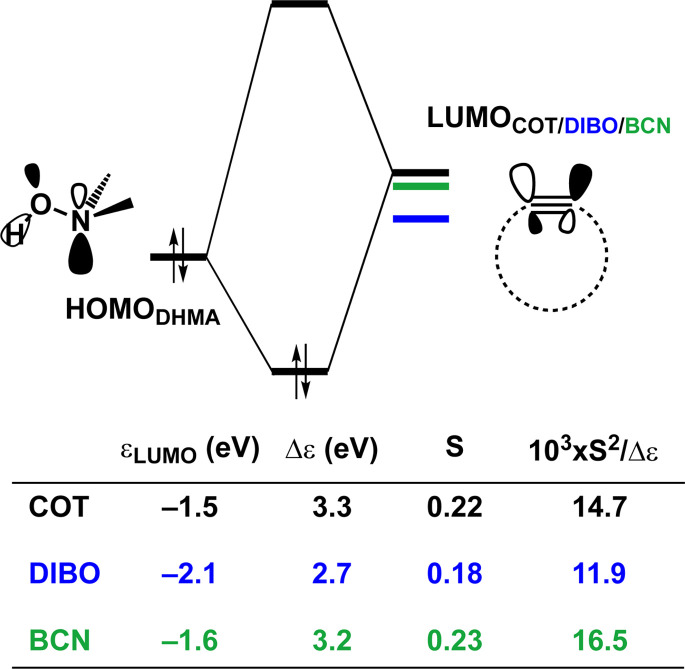
Schematic diagrams with LUMO energies, energy gaps Δε, overlaps S, and values of S^2^/Δε term associated with the key donor–acceptor interaction between HOMO_DHMA_|LUMO_alkyne_ along with the retro-Cope elimination reactions between cyclooctyne (COT), DIBO and BCN with DMHA. All were computed at the consistent geometries where the length of the C⋯H bond is 1.50 Å at ZORA-BP86/TZ2P.

Hamlin and coworkers have shown that, in addition to the reduction of the destabilizing strain, a more stabilizing interaction energy is at the root of the enhanced reactivity in 1,3-dipolar cycloaddition between azides and strained alkynes (SPAAC).^30^ Energy decomposition analyses in conjunction with KS-MO analyses illustrated that the bending of alkynes has a profound effect on the frontier molecular orbitals (FMOs), *i.e.*, the LUMO 
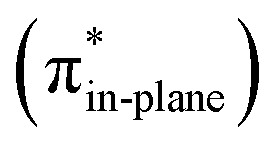
 is stabilized and the HOMO (π_in-plane_) is slightly destabilized because of mixing with the adjacent σ*- and σ-orbitals, respectively. Thus, a more bent alkyne has a lower LUMO_alkyne_, giving rise to a smaller HOMO_DMHA_–LUMO_COT_ gap. Therefore, the reaction of BCN, which has a 3.0° smaller internal C–C≡C angle at equilibrium geometry, goes with a smaller initial HOMO_DMHA_–LUMO_BCN_ gap than the reaction of COT (Fig. 3). It must be noted that, with the exception of DIBO, the 
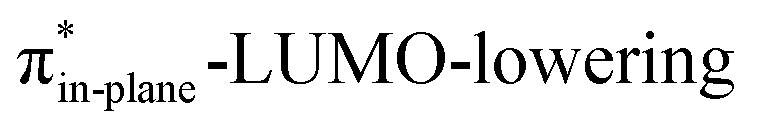
 along the reaction coordinate (the slope), relative to the energy at equilibrium geometry, is approximately equal (Fig. S4d†). As for BCN the Δ*r* remains equal, this leads to a larger S^2^/Δ*ε* term, *i.e.*, the more favorable HOMO_DMHA_–LUMO_BCN_ interaction and, consequently, more stabilizing orbital interactions associated with the retro-Cope elimination reaction between BCN and DMHA.

Despite DIBO having a lower 
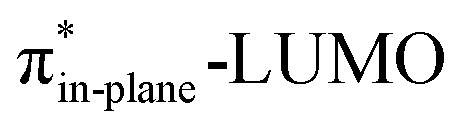
 energy than COT, the overall orbital interactions for the DIBO reaction are less stabilizing (Fig. 3). This can be rationalized by the reduced HOMO_DMHA_–LUMO_DIBO_ orbital overlap brought about by the C⋯N bond formation distance in the reaction of DIBO (Δ*r* = 0.76 Å) lagging substantially behind that of COT (Δ*r* = 0.67 Å). This geometric effect also brings about considerably less destabilizing Pauli repulsion. At a double consistent geometry (Table S4†), where Δ*r* is equal to that of COT at consistent geometry (Δ*r* = 0.67 Å), the Pauli repulsion exceeds the more stabilizing orbital and electrostatic interaction. This is due to the overlap of various filled in-plane orbitals of the DMHA fragment overlapping with the HOMO−4, HOMO−2 and, HOMO−1 of DIBO (Fig. S7†). It is the buildup of this Pauli repulsion that is effectively absorbed into the reaction path causing the more slanted reaction path (higher Δ*r*). As a result, the Pauli repulsion is lowered, and, concomitantly, the orbital overlaps between the two fragments are reduced, also decreasing the HOMO_DMHA_–LUMO_DIBO_ overlap. Similar to the retro-Cope elimination reaction with linear alkynes with added steric bulk,^28^ the Pauli lowering effect originates in more (not less) steric repulsion introduced by the fused aryls in DIBO.

We observe that the additional repulsive interaction occurs between the various in-plane orbitals of DMHA and the HOMO−1, HOMO−2, and HOMO−4 orbitals in DIBO. In contrast, the literature attributes this repulsive effect to “flagpole” hydrogens.^6*d*,32^ At double consistent geometry, the occupied–occupied orbital overlaps that actually increase are not in the vicinity of the “flagpole” hydrogen atoms. Instead, the three orbitals are combinations of the π_in-plane_-HOMO of COT and the π-HOMOs of the benzene substituents. The π_in-plane_-HOMO of COT is allowed to mix with the π-system benzene substituents due to the twisted nature of DIBO (Fig. S7†).

Thus, although the introduction of fused benzenes causes a substantially smaller HOMO_DMHA_–LUMO_DIBO_ gap (Fig. 3), the more stabilizing orbital interaction are hampered by lowered HOMO_DMHA_–LUMO_DIBO_ overlaps, due to the C⋯N bond forming distance in DIBO lagging behind that of COT. The greater C⋯N bond forming distance can be traced back to the increased two-center four-electron Pauli repulsion, which is absorbed into the geometry of the reaction system by forcing it into a slanted reaction path. As a result, the overlap between the filled FMOs decreases, ultimately reducing Pauli repulsion, which outweighs the concurrent decrease in stabilizing orbital and electrostatic interactions. The smaller HOMO_DMHA_–LUMO_DIBO_ gap in DIBO, together with the introduction of additional HOMO–LUMO interactions, limit the destabilization by the additional in-plane steric bulk between the two fragments.

Considering that orbital interactions play a key role in the enhanced reactivity of DIBO and BCN compared to COT, we performed an additional analysis on the key π*-LUMO_in-plane_ that engages with the π-HOMO_DHMA_. We sought to determine the factors that lead to the observed lowering of the π*-LUMO_in-plane_ orbital energies of DIBO and BCN relative to COT. To do so, we constrained COT in the equilibrium geometries of the respective substituted cyclic alkynes and reported the π-HOMO_in-plane_ and π*-LUMO_in-plane_ orbital energies in Fig. S4c.† The root of the π*-LUMO_in-plane_-lowering in the equilibrium geometry was further analyzed by decomposing the change in orbital energy, Δε_MO_, into substituent, Δε_sub_, and bending, Δε_deform_, contributions, which were defined as (Fig. S4a and b†):

4 Δε_MO_ = Δε_MO,predist_ + Δε_MO,sub_

5 Δε_MO,predist_ = ε_MO_(COT_constrained_) − ε_MO_ (COT_eq_)

6 Δε_MO,sub_ = ε_MO_ (sub) − ε_MO_ (COT_constrained_)

At the equilibrium geometry, the π*-LUMO_in-plane_-lowering of BCN is primarily caused by the additional bending of the alkyne enforced by the fused cyclopropane (0.14 eV (79%)) and only lowered by 0.04 eV (21%) because of the addition of the fused cyclopropane to the deformed cyclooctyne. In contrast, the π*-LUMO_in-plane_-lowering in DIBO primarily finds its origin in substituent effects instead of the additional bending of the alkyne. In equilibrium geometry, DIBO is stabilized by the bending of the alkyne enforced by the more rigid cyclic backbone by only 0.24 eV (23%), whereas the substitution by the dibenzo-substituents accounts for a stabilization of 0.78 eV (77%).

Additionally, the π*-LUMO_in-plane_ orbital energies along the reaction coordinate associated with the retro-Cope elimination reaction are shown in Fig. S4d.† The orbital energy profiles of the π*-LUMO_in-plane_ orbitals look alike with similar steepness along the reaction coordinate and differ primarily in the π*-LUMO_in-plane_ orbital energy in the equilibrium geometry. DIBO stands out in that the steepness is lower, *i.e.*, the rigid DIBO is less affected by deformation of the alkyne along the reaction coordinate. This is likely due to the smaller angle deformation needed for consistent geometry (Fig. 2a), resulting from the more slanted reaction path (larger Δ*r*).

### Effect of exocyclic propargylic substitution

Next, we discuss the modulation of COT at the exocyclic propargylic position. We studied the effect of appending a hydroxy group (HO-COT) and difluoro group (DIFO) at the exocyclic propargylic position of COT. Cyclooctynes activated by exocyclic propargylic substitution prefer the *anti*-Markovnikov adduct (*anti*-pathway) over the Markovnikov adduct (*syn*-pathway) in the retro-Cope elimination reaction with DMHA (Tables S1 and S2†), as such, we focus on the retro-Cope elimination reaction along the *anti*-pathways. The relative electronic energies for the reactant complex (Δ*E*_RC_), activation barriers (Δ*E*^‡^), and the reaction energies (Δ*E*_rxn_) for cyclooctynes substituted on the exocyclic propargylic position towards the retro-Cope elimination reaction along the *anti*-pathway are shown in Table 3. Replacing the exocyclic propargylic hydrogen with the more electronegative HO-COT and DIFO lowers the activation barrier from 4.6 to 2.3 and 0.2 kcal mol^−1^, respectively.

**Table 3:** Transition state structures for the retro-Cope elimination reactions, Δ*r* in the transition state (in Å), electronic energies relative to reactants of the reactant complex Δ*E*_RC_ (in kcal mol^−1^), transition state Δ*E*^‡^ (in kcal mol^−1^), product Δ*E*_rxn_ (in kcal mol^−1^), Gibbs free energies of activation Δ*G*^‡^ (in kcal mol^−1^) and the second-order rate constants relative to COT, *k*_rel_, for retro-Cope elimination reactions of X-COT with DMHA along *anti*-pathways^a^

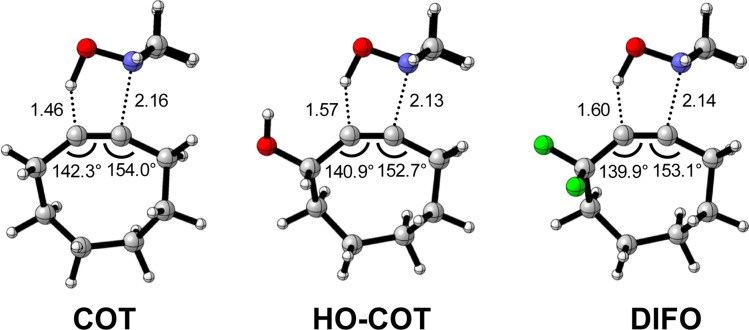			
	COT	HO-COT	DIFO
Δ*r*^b^	0.70	0.56	0.54
Δ*E*_RC_	−3.0	−4.9	−2.1
Δ*E*^‡^	4.6	2.3	0.2
Δ*E*_rxn_	−14.4	−15.3	−15.9
Δ*G*^‡^	15.8	14.6	13.0
*k*_rel_ c	1.0	8.7	1.3 × 10^2^

a Computed at ZORA-BP86/TZ2P.

b Δ*r* is the difference in length between the forming C⋯N and C⋯H bonds at the transition state.

c The relative second-order rate constants (*k*_rel_) were calculated relative to the parent propyne (*k*/*k*_COT_). Only the anti-pathway was considered.

**Fig. 4:**
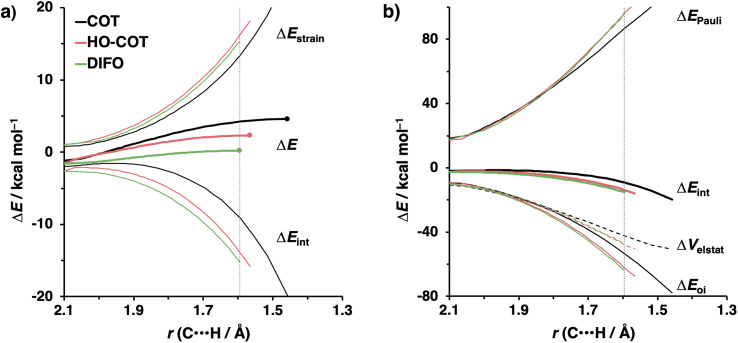
(a) Activation strain and (b) energy decomposition analyses for the retro-Cope elimination reactions of COT, HO-COT, and DIFO with DHMA along *anti*-pathways. Transition states are indicated by dots. The energy terms along the IRC are projected on the length of the newly forming C⋯H bond and the vertical dotted line indicates the point at which the length of C⋯H bond is 1.60 Å. Computed at ZORA-BP86/TZ2P.

Kang *et al.* suggested that the reaction does not benefit from a more stabilizing interaction energy upon the addition of electronegative substituents but, rather, from a decrease in strain energy.^10*a*^ Our analysis at the transition states also furnishes the same insight. However, performing our activation strain and energy decomposition analyses along the full reaction coordinate provides us with a more comprehensive picture of the factors dictating the reactivity.^22*a*^ In Fig. 4a, the ASM diagram reveals that, at consistent geometry, the activation strain follows a trend opposite to the activation barriers. This would indicate, at odds with Kang *et al.*^10*a*^ that the strain is not responsible for observed rate enhancements, but instead it is the progressively more stabilizing interaction energy going from COT to HO-COT to DIFO that is key for the enhanced reactivity. Because the HO-COT and DIFO benefit from more stabilizing interaction energy, the transition states shift to an early point on the reaction coordinate towards the reactants. This, in turn, causes the reactants to be less structurally deformed in their respective TSs.

Our energy decomposition analyses (EDA) (Fig. 4b) show that the more stabilizing Δ*E*_int_ term associated with the retro-Cope elimination reactions of HO-COT and DIFO arises from a more stabilizing Δ*E*_oi_, reinforced by a smaller contribution from the Δ*V*_elstat_. The reverse trend in Δ*E*_Pauli_ to that of Δ*E*_int_ shows that Δ*E*_Pauli_ is not responsible for the more stabilizing interaction energy.

**Fig. 5:**
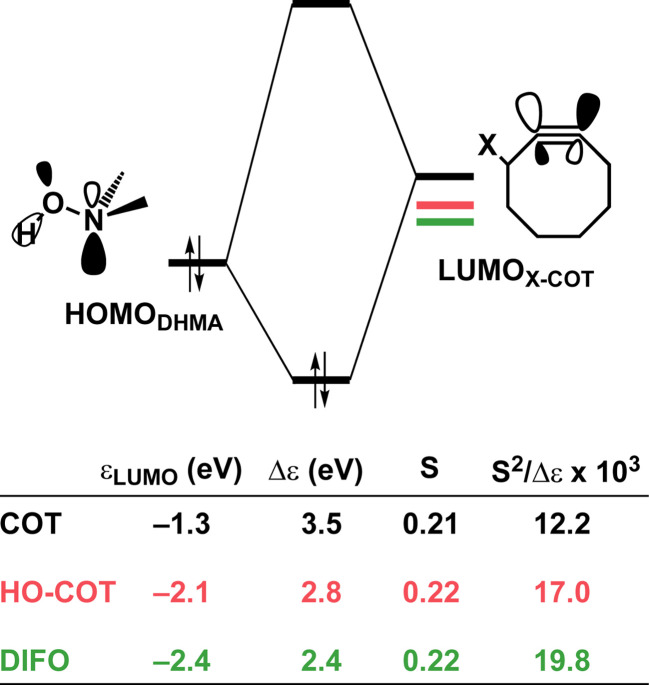
Schematic diagrams with LUMO energies, energy gaps Δε, overlaps S, and values of 10^3^ × S^2^/Δε term associated with the key donor–acceptor interaction between HOMO_DHMA_–LUMO_X-COT_ along with the retro-Cope elimination reactions between COT, HO-COT, and DIFO with DMHA. All were computed at the consistent geometries where the length of the C⋯H bond is 1.60 Å at ZORA-BP86/TZ2P.

To determine the origin of the enhanced orbital interactions, we quantified all donor–acceptor orbital interactions associated with the retro-Cope elimination reaction of COT, HO-COT, and DIFO at consistent geometry where C⋯H bond forming distance is 1.60 Å and confirmed the HOMO_DMHA_–LUMO_X-COT_ interaction as the most important contributor (Fig. S8†). Further KS-MO analyses reveal that the enhanced orbital interactions of HO-COT and DIFO compared to COT originate in their lowered LUMOs and a slightly more efficient orbital overlap. Especially the former brings about a larger orbital interaction term (∝S^2^/Δε term) as the energy gap reduces from 3.5 to 2.8 to 2.4 eV going from COT to HO-COT to DIFO (Fig. 5). The origin of the π*-LUMO_in-plane_-lowering in the equilibrium geometry was further analyzed by decomposing the change in orbital energy into, Δε_π*-LUMO_, into substituent, Δε_sub_, and bending, Δε_bend_, contributions. We confirmed that the LUMO-lowering effect principally stems from the substituent effect and not the deformation of the alkyne (Fig. S4c†).

### Effect of endocyclic propargylic substitution

Lastly, we analyzed the reactivity of a group of endocyclic heterocyclooctynes (Y-COT) toward the retro-Cope elimination reaction with DMHA. We substitute the endocyclic propargylic carbon with the nitrogen (N-COT), oxygen (O-COT), and sulfur (S-COT). Analogous to the cyclooctynes activated by exocyclic propargylic substitution (Tables S1 and S2†), we find that the cyclooctynes activated by endocyclic propargylic substitution prefer the *anti*-Markovnikov adduct (*anti*-pathway) over the Markovnikov adduct (*syn*-pathway) in the retro-Cope elimination reaction with DMHA (Tables S1 and S2†). Therefore, we center our analysis on the retro-Cope elimination reaction along the *anti*-pathways. Table 4 presents the relative electronic energies for the reactant complex (Δ*E*_RC_), activation barriers (Δ*E*^‡^), and the reaction energies (Δ*E*_rxn_) for Y-COT cyclooctynes towards the retro-Cope elimination reaction along the *anti*-pathway. Replacing the endocyclic propargylic carbon COT with the more electronegative N-COT and O-COT lowers the activation barrier from 4.6 kcal mol^−1^ to 2.9 and 1.1 kcal mol^−1^, respectively. Incorporation of a sulfur heteroatom (S-COT) at the endocyclic propargylic position raises the activation barrier to 5.9 kcal mol^−1^.

**Table 4:** Transition state structures for the retro-Cope elimination reactions, Δ*r* in the transition state (in Å), electronic energies relative to reactants of the reactant complex Δ*E*_RC_ (in kcal mol^−1^), transition state Δ*E*^‡^ (in kcal mol^−1^), product Δ*E*_rxn_ (in kcal mol^−1^), Gibbs free energies of activation Δ*G*^‡^ (in kcal mol^−1^) and the second-order rate constants relative to COT, *k*_rel_, for retro-Cope elimination reactions of Y-COT with DMHA along *anti*-pathways^a^

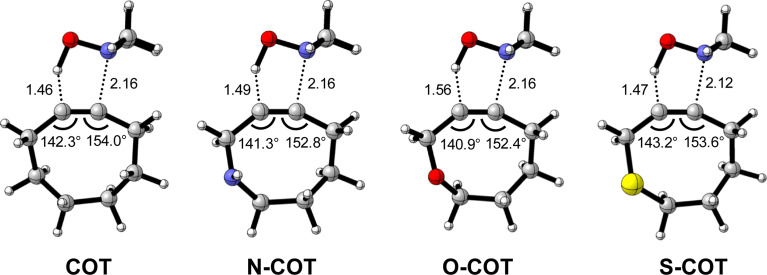				
	COT	N-COT	O-COT	S-COT
Δ*r*^b^	0.70	0.67	0.59	0.66
Δ*E*_RC_	−3.0	−2.9	−2.7	−2.7
Δ*E*^‡^	4.6	2.9	1.0	5.8
Δ*E*_rxn_	−14.4	−15.8	−19.5	−13.4
Δ*G*^‡^	15.8	14.3	13.1	17.0
*k*_rel_ c	1.0	13.7	95.3	0.1

a Computed at ZORA-BP86/TZ2P.

b Δ*r* is the difference in length between the forming C⋯N and C⋯H bonds at the transition state.

c The relative second-order rate constants (*k*_rel_) were calculated relative to COT (*k*/*k*_COT_). Only the *anti*-pathway was considered.

**Fig. 6:**
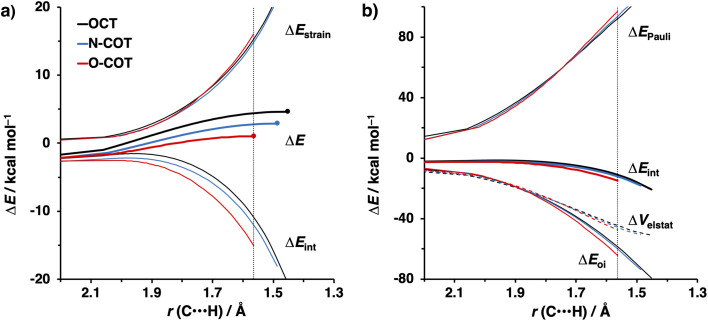
(a) Activation strain diagram and (b) energy decomposition analyses for the retro-Cope elimination reactions of the COT, N-COT, and O-COT with DHMA along *anti*-pathways. Transition states are indicated by dots. The energy terms along the IRC are projected on the length of the newly forming C⋯H bond and the vertical dotted line indicates the point at which the length of C⋯H bond is 1.56 Å. Computed at ZORA-BP86/TZ2P.

To gain a quantitative understanding of the physical factors that are at the root of the trends in reactivity, we again turned to ASM. In Fig. 6a, the activation strain diagram highlights that the reduction in activation barrier height from COT to N-COT to O-COT cyclooctyne is driven by an increasingly stabilizing Δ*E*_int_ term. Despite observing a more favorable interaction energy for the S-COT cyclooctyne at consistent geometry, this is offset by the considerable increase in activation strain (Fig. S9†). The larger atomic radius of the sulfur relative to oxygen allows the alkyne to be less bent *i.e.*, less predistorted, and consequently, a greater structural deformation of the Δ∠(C–C≡C) is necessary at consistent geometry (Table S6†). Consequently, the reduced reactivity of S-COT towards the retro-Cope elimination reaction is attributed to the higher activation strain.

Our canonical EDA analyses (Fig. 6b) reveal that the more stabilizing Δ*E*_oi_ is responsible for the progressively more stabilizing Δ*E*_int_ term associated with the retro-Cope elimination reaction going from COT to N-COT to O-COT. The differences in the Δ*V*_elstat_ curves are minimal, whereas the Δ*E*_Pauli_ shows a reverse trend to that of Δ*E*_int_ and is, thus, not responsible for the more stabilizing interaction energy.

**Fig. 7:**
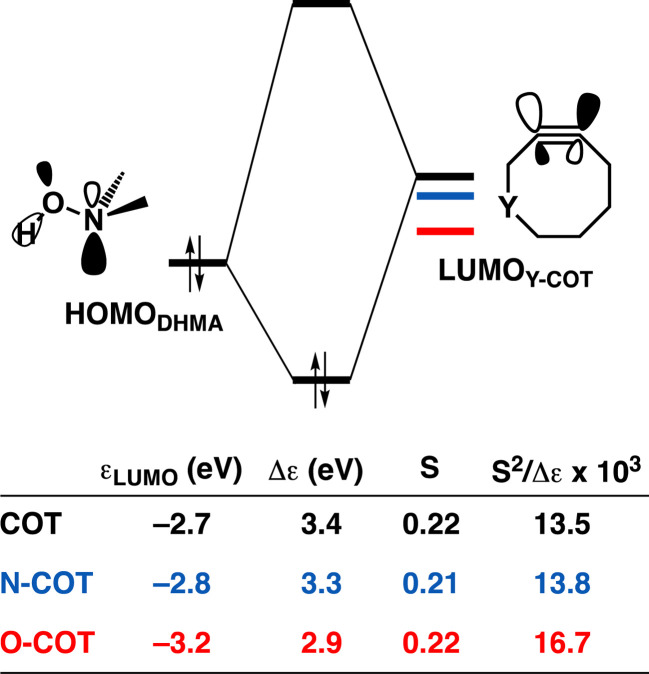
(a) Schematic orbital-interaction diagrams with energy gaps Δε, overlaps S, values of 10^3^ × S^2^/Δε of the individual HOMO_DHMA_ and LUMO_alkyne_ interactions associated with the retro-Cope elimination reactions between DMHA and the original cyclooctyne COT, N-COT and O-COT. All were computed at the consistent geometries where the length of the C⋯H bond is 1.56 Å at ZORA-BP86/TZ2P.

As the more stabilizing orbital interactions were identified as a key factor that dictated the enhanced reactivity of Y-COT, we screened all orbital donor–acceptor interactions associated with the retro-Cope elimination reactions of COT, N-COT, and O-COT at consistent geometry where the length of the forming C⋯H bond is 1.56 Å (Fig. S10†). The HOMO_DMHA_–LUMO_Y-COT_ interaction was confirmed as the most important contributor. The KS-MO analyses at the consistent geometries reveal that it is the consistent lowering of the π*-LUMO_alkyne_ of the Y-COT cyclooctyne that causes the more favorable orbital interactions between HOMO_DMHA_ and LUMO_Y-COT_ (Fig. 7). Because of the lowering of the 
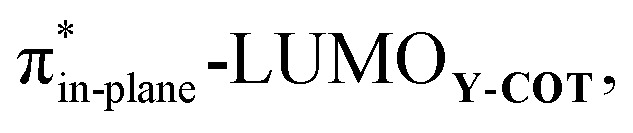
 the HOMO_DMHA_–LUMO_Y-COT_ energy gap is reduced from Δε = 2.6 to 2.4 to 2.1 eV. The orbital overlap is minimally affected by substitution on the endocyclic propargylic position. As the changes in orbital overlaps remain limited, we can conclude that the enhanced orbital interactions of N-COT and O-COT in retro-Cope elimination reactions can directly be ascribed to the LUMO_Y-COT_-lowering effect of the endocyclic propargylic substitution, which causes the smaller HOMO_DMHA_–LUMO_Y-COT_ energy gaps.

### Next-generation bioorthogonal reagents

Next, with our new design principles in hand, we sought to generate a suite of novel reagents that exhibit enhanced retro-Cope reactivity relative to current state-of-the-art reagents. Firstly, we propose the judicious combination of rigid cyclic substituents in tandem with both endo- and exocyclic heteroatoms. Based on this, we present 3,3-difluoro-BCN (DF-BCN) and a cyclooctyne annulated with single aryl substituent combined with difluoro and oxide substituents on the exo- and endocyclic propargylic position, respectively (DF-O-MOBO). Based on our computations, we predict a that the reactivity is further enhanced (relative to COT) by ΔΔ*E*^‡^ = 6.1 and 5.2 with *k*_rel_ values of 2.6 × 10^3^ and 5.2 × 10^2^, respectively. Fusion of aryl groups on the carbons 5 and 6 of the cyclooctyne with difluoro-substituents on the exocyclic propargylic position (DF-*p*-MOBO) furnished a reagent with a remarkable *k*_rel_ value of 4.7 × 10^4^.^32*b*^ Additionally, substitution by chloro (DCl-BCN, DF-*p*-MOBO) and trifluoromethyl (DCF_3_-BCN, DCF_3_-*p*-MOBO) substituents enhanced the *k*_rel_ values up to four orders of magnitude.

**Scheme 3:**
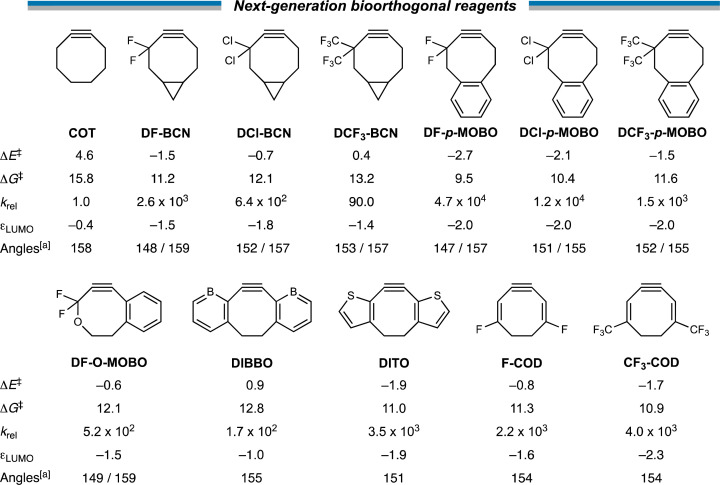
Our suite of novel reagents that exhibit enhanced retro-Cope reactivity with dimethylhydroxylamine (DMHA) relative to current state-of-the-art reagents. The π_in-plane_-LUMO energies in the equilibrium geometry (in eV), the internal angles related to the predistortion of the alkyne (in °), the electronic (Δ*E*^‡^) and Gibbs free energies Δ*G*^‡^ of activation (in kcal mol^−1^) and the second-order rate constants relative to unsubstituted parent COT for retro-Cope elimination reactions with DMHA along anti-pathways. Computed at ZORA-BP86/TZ2P. ^*a*^ A single internal angle is given for the cyclic alkynes that are *C*_2_-symmetric.

Additionally, we propose modulations of the DIBO to employ the strong LUMO-lowering effect that is observed in DIBO. By replacing the two aryl rings by borabenzenes (DIBBO) and thiophenes (DITO) the reactivity was enhanced (relative to COT) by ΔΔ*E*^‡^ = −3.7 and −6.5 kcal mol^−1^ with *k*_rel_ values of 1.7 × 10^2^ and 3.5 × 10^3^, respectively. Lastly, we added strongly electron withdrawing groups *i.e.*, fluoro (F-COD) and trifluoromethyl (CF_3_-COD) substituents on a cycloocta-1,5-dien-3-yne (COD), significantly lowering the π_in-plane_-LUMO, without adding additional bulk in the vicinity of the triple bond. This resulted *k*_rel_ values of 2.2 × 10^3^ and 4.0 × 10^3^, respectively (Scheme 3).

## Conclusions

Our theoretical study reveals that the retro-Cope elimination reaction between dimethylhydroxylamine (DMHA) and cyclic alkynes can be accelerated by judicious functionalization of the alkyne. We explored three distinct activation modes, (i) additional predistortion of the alkyne by fused cycles and decorating the cycloalkyne with (ii) exocyclic heteroatom substituents, and (iii) endocyclic heteroatom substitution on the cycloalkyne. Our design principles and judicious combination of these activation modes were used to rationally design a suite of novel bioorthogonal reagents that feature second-order rate constants that outperform all other current reagents in these retro-Cope elimination reactions.

Our activation strain and Kohn–Sham molecular orbital analyses identified the enhanced retro-Cope elimination reactivity upon predistortion of the alkyne (linear to cyclic) arises from a reduction in activation strain and a strengthening of stabilizing orbital interactions. Predistortion of the alkyne *via* cyclization induces a stabilization of the propyne π*-LUMO_alkyne_, which results in a smaller, more favorable HOMO_DMHA_–LUMO_alkyne_ gap and thus more stabilizing inverse electron demand (IED) orbital interactions. Appending the COT with fused cycles imparts additional predistortion of the alkyne, leading to even less activation strain and even more stabilizing IED orbital interactions. Decorating COT with exo- and endocyclic heteroatom substituents also stabilizes the π*-LUMO_alkyne_, and consequently further enhances the stabilizing IED orbital interactions.

A secondary effect is observed in the case of DIBO, a dibenzo-annulated cyclooctyne, which exhibits a more pronounced decrease in the activation strain that occurs at the consistent geometry, that is, a consistent point along the reaction coordinate (*r*(C⋯H)), relative to BCN (bicyclononyne). This can be related to the more slanted reaction path, where the formation of the C⋯N bond lags behind that of the C⋯H bond because of the following: Due to the twisted nature of DIBO, the π_in-plane_-HOMO of COT mixes in with the π-system of the benzene substituents, heightening the steric Pauli repulsion. The strong repulsive interaction is absorbed by adapting the reaction system to a more slanted reaction path. As a consequence, the overlaps are reduced, thereby lowering both the repulsive Pauli repulsion and the favorable orbital interactions. Notably, because of this more slanted reaction path, both reactants are less distorted at consistent geometry, causing a more pronounced decrease in the activation strain.

Our rational design principles allowed us to construct *a priori* a suite of novel bioorthogonal reagents that are highly reactive towards the retro-Cope elimination reaction with DMHA. Based on the judicious substitution of the parent COT, our novel reagents exhibit reactivities that are two to four orders of magnitude greater than COT.

## Data availability

The datasets supporting this article have been uploaded as part of the ESI.†

## Author contributions

TAH conceived the project. SEB and SY carried out the quantum chemical computations and bonding analyses. SEB, SY, and TAH drafted the manuscript. All authors discussed the results and reviewed the manuscript.

## Conflicts of interest

The authors declare no conflict of interest.

## Supplementary Material

SC-OLF-D4SC04211E-s001
