# Supplementary material for: Retro-Cope elimination of cyclic alkynes: reactivity trends and rational design of next-generation bioorthogonal reagents
Source: Chem Sci. 2024 Aug 27;15(37):15178–91. doi: 10.1039/d4sc04211e (PMC11369967; doi:10.1039/d4sc04211e)
Supplement: SC-015-D4SC04211E-s001 [file SC-015-D4SC04211E-s001.pdf]

Supplementary Information (SI) for Chemical Science.
This journal is © The Royal Society of Chemistry 2024

Retro-Cope Elimination of Cyclic Alkynes: Reactivity Trends and Rational Design of Next-Generation Bioorthogonal Reagents

Steven E. Beutick,[+,a,b] Song Yu,[+,a] Laura Orian,[b] F. M. Bickelhaupt,[a,c,d] Trevor A. Hamlin*[a]

[a] *S. E. Beutick, Dr. S. Yu, Prof. Dr. F. M. Bickelhaupt, Dr. T. A. Hamlin,*
Department of Chemistry and Pharmaceutical Sciences, Amsterdam Institute of Molecular and Life Sciences (AIMMS), Vrije Universiteit Amsterdam, De Boelelaan 1108, 1081 HZ Amsterdam, The Netherlands.
E-mail: t.a.hamlin@vu.nl

[b] *S. E. Beutick, Prof. Dr. L. Orian*
Dipartimento di Scienze Chimiche, Università degli Studi di Padova, Via Marzolo 1, 35129 Padova, Italy.

[c] *Prof. Dr. F. M. Bickelhaupt*
Institute of Molecules and Materials, Radboud University, Heyendaalseweg 135, 6525 AJ Nijmegen, The Netherlands.

[d] *Prof. Dr. F. M. Bickelhaupt*
Department of Chemical Sciences, University of Johannesburg, Auckland Park, Johannesburg 2006, South Africa.

[+] *These authors contributed equally to this work*

Contents

Example input file

Fig. S1. The optimized structures of (cyclo)alkynes and **DMHA** which are the studied reagents in the retro-Cope reaction computed at ZORA-BP86/TZ2P.

Fig. S2. Transition state structures for the retro-Cope reactions of 2-butyne and the studied cyclic octynes with **DMHA** along *anti*-pathways, computed at ZORA-BP86/TZ2P.

Table S1. Energies relative to reactants of the reactant complex ΔE_{RC} (kcal mol^{-1}), transition state $\Delta E^{\ddagger}$ (kcal mol^{-1}), and product ΔE_{rxn} (kcal mol^{-1}) for the retro-Cope reactions of propyne, 2-butyne and the original cyclooctyne **OCT** and functionalized cyclooctynes with along *anti*- and *syn*-pathways.[a]

Table S2. Gibbs free energies relative to reactants of the reactant complex ΔG_{RC} (kcal mol^{-1}), transition state $\Delta G^{\ddagger}$ (kcal mol^{-1}), and product ΔG_{rxn} (kcal mol^{-1}) for the retro-Cope reactions of propyne, 2-butyne and the original cyclooctyne **OCT** and functionalized cyclooctynes with along *anti*- and *syn*-pathways.[a]

Table S3. Activation energies $\Delta E^{\ddagger}$ (kcal mol^{-1}) for the retro-Cope reactions of propyne, 2-butyne and the original cyclooctyne **OCT** and functionalized cyclooctynes with **DHMA** along *anti*-pathways, computed at various levels of theory.

Fig. S3. a) Activation strain model and b) decomposition of strain energies for the retro-Cope reactions of 2-butyne and **COT** with **DMHA**. Transition states are indicated by dots. The energy terms along the IRC are projected on the length of the newly forming C···H bond and the vertical dotted line indicates the point at which the length of the C···H bond is 1.45 Å. c) Schematic diagrams with LUMO energies, energy gaps Δε, overlaps S, and values of $S^2/\Delta\varepsilon$ term associated with the key donor–acceptor interaction between $HOMO_{\mathbf{DHMA}}$–$LUMO_{alkyne}$ along with the retro-Cope reactions between 2-butyne and **COT** with **DMHA**. All were computed at ZORA-BP86/TZ2P.

Fig. S4. A schematic representation of the formula that decomposes the change in orbital energies into a) a deformation ($\Delta\varepsilon_{deform}$) and b) a substituent ($\Delta\varepsilon_{sub}$) contribution. c) The π-HOMO and π*-LUMO energies (eV) for the cyclic alkynes in their equilibrium geometry (ε_{alkyne}) and the π-HOMO and π*-LUMO energies for cyclooctyne constrained in the equilibrium geometry of the alkynes. d) The π*-LUMO energies (eV) of the alkyne fragment associated with the retro-Cope reaction between strained alkynes and **DMHA** projected on the length of the newly forming C···H bond. The TSs are indicated by dots. Computed at ZORA-BP86/TZ2P.

Fig. S5. Heatmaps for computed values of $S^2/\Delta\varepsilon$ associated with the donor–acceptor interactions along with the retro-Cope reactions of a) **COT**, b) **DIBO**, and c) **BCN** with **DMHA** at the consistent geometries where the length of C···H bond is 1.50 Å. All were computed at ZORA-BP86/TZ2P.

Table S4. The energy decomposition analysis (in kcal mol^{-1}) of the retro-Cope reactions of the original **COT** and **DIBO** with **DMHA** at various consistent and double consistent geometries.

Fig. S6. Heatmaps for closed-shell orbital overlaps, S, between the filled FMOs of **DMHA** with the filled FMOs of a) **DIBO** at consistent geometry where C···H bond is 1.50 Å and b) **DIBO** in a double consistent geometry associated with the retro-Cope reactions along the anti-pathways. Computed at ZORA-BP86/TZ2P.

Fig. S7. The HOMO orbitals in **DIBO** that increase in Pauli repulsive overlap between the in-plane orbitals of **DMHA** going from CG to DCG at a C···H bond distance of 1.50 Å.

Fig. S8. Heatmaps for computed values of $S^2/\Delta\varepsilon$ associated with the donor–acceptor interactions along with the retro-Cope reactions of a) **COT**, b) **HO-COT**, and c) **DIFO** with **DMHA** along the *anti*-pathways at the consistent geometries where the length of C···H bond is 1.56 Å. All were computed at ZORA-BP86/TZ2P.

Table S5. The energy decomposition analysis (in kcal mol^{-1}) of the retro-Cope reactions of the original **COT, HO-COT** and **DIFO** with **DMHA** at various consistent and double consistent geometries.

Table S5. The energy decomposition analysis (in kcal mol^{-1}) of the retro-Cope reactions of the original **COT, HO-COT** and **DIFO** with **DMHA** at various consistent and double consistent geometries.

Fig. S9. a) Activation strain model, b) energy decomposition analyses and c) decomposition of strain energies for the retro-Cope reactions of **COT**, **N-COT**, **O-COT** and **S-COT** with **DMHA**. Transition states are indicated by dots. The energy terms along the IRC are projected on the length of the newly forming C···H bond and the vertical dotted line indicates the point at which the length of the C···H bond is 1.56 Å. All were computed at ZORA-BP86/TZ2P.

Table S6. The structural deformation of key angles (in °) and bonds distances (in Å) from equilibrium geometries to consistent geometries, where the lengths of the C···H bond are 1.56 Å, associated with the retro-Cope reactions of **COT**, **N-COT**, **O-COT** and **S-COT** with **DMHA** along the *anti*-pathways. Computed at ZORA-BP86/TZ2P.

Fig. S10. Heatmaps for computed values of $S^2/\Delta\varepsilon$ associated with the donor–acceptor interactions along with the retro-Cope reactions of a) **COT**, b) **N-COT**, c) **O-COT** and d) **S-COT** with **DMHA** along the anti-pathways at the consistent geometries where the length of C···H bond is 1.56 Å. All were computed at ZORA-BP86/TZ2P.

Table S7. The energy decomposition analysis (in kcal mol^{-1}) of the retro-Cope reactions of the original **COT, N-COT** and **O-COT** with **DMHA** at various consistent and double consistent geometries.

Table S8. The total electronic energies (kcal mol^{-1}), enthalpies (kcal mol^{-1}), Gibbs free energies (kcal mol^{-1}) relative to the spherical average-of-configuration atoms, the number of imaginary vibrational frequencies (and their wavelength), and cartesian coordinates (Å), for all stationary points (reactant complex RC, transition state TS, product P), computed at ZORA-BP86/TZ2P using AMS2021.

Example input file

```
Task GeometryOptimization

System
    Atoms
        [cartesian coordinates (Å)]
    End
End

GeometryOptimization
    MaxIterations 199
    Convergence
        Gradients 1e-05
        Energy 1e-05
    End
End

Properties
    NormalModes Yes
End
NormalModes
    Hessian Analytical
    ReScanFreqRange -1000 10.0
End

Engine ADF
    Basis
        Type TZ2P
        Core None
    End
    XC
        GGA BP86
    End
    NumericalQuality VeryGood
    SCF
        Iterations 199
        Converge 1e-05
    End
EndEngine
```

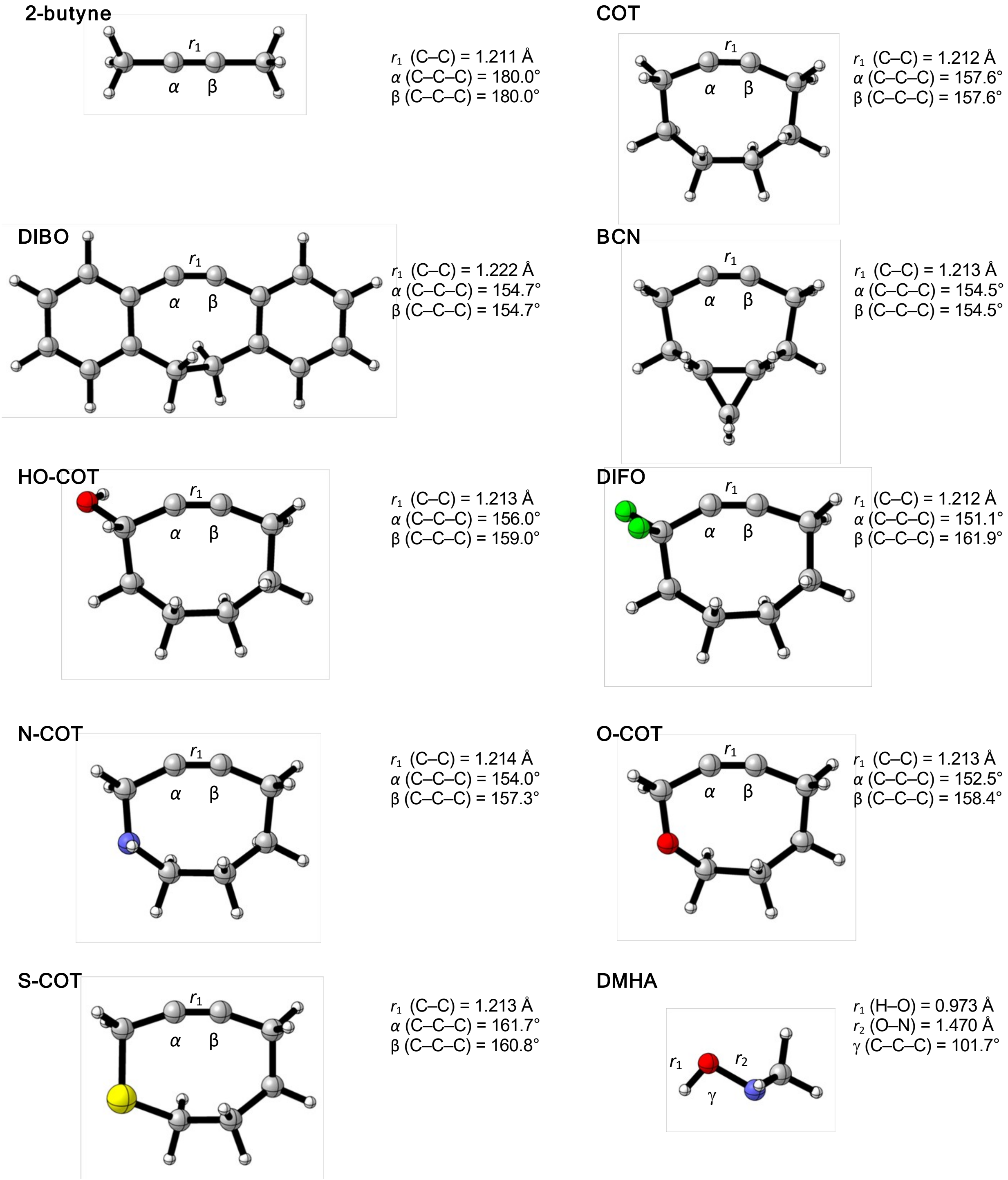

Fig. S1. The optimized structures of (cyclo)alkynes and **DMHA** which are the studied reagents in the retro-Cope reaction computed at ZORA-BP86/TZ2P.

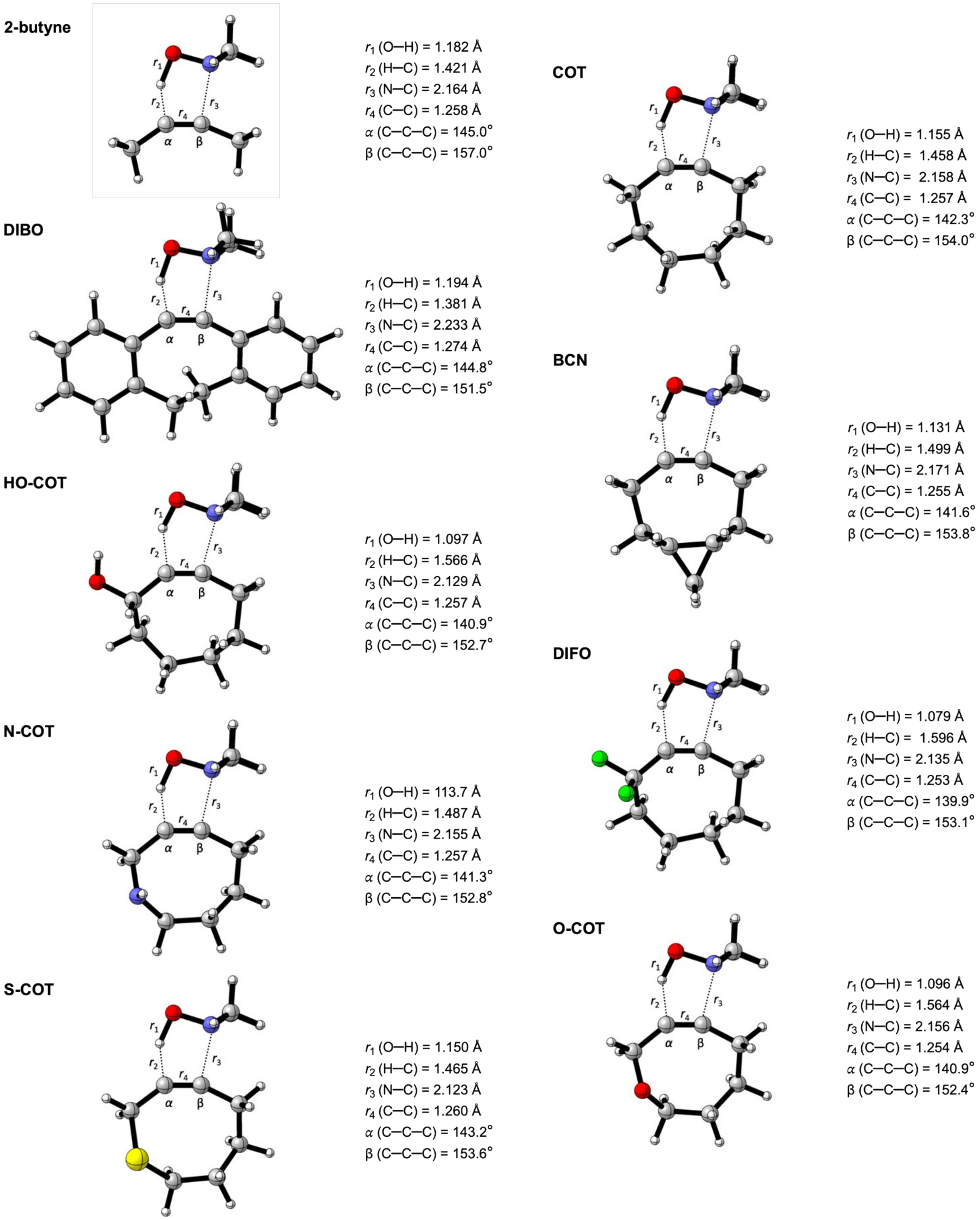

Fig. S2. Transition state structures for the retro-Cope reactions of 2-butyne and the studied cyclic octynes with **DMHA** along *anti*-pathways, computed at ZORA-BP86/TZ2P.

Table S1. Energies relative to reactants of the reactant complex ΔE_{RC} (kcal mol^{-1}), transition state $\Delta E^{\ddagger}$ (kcal mol^{-1}), and product ΔE_{rxn} (kcal mol^{-1}) for the retro-Cope reactions of propyne, 2-butyne and the original cyclooctyne **OCT** and functionalized cyclooctynes with along *anti*- and *syn*-pathways.[a]

Alkyne	*anti*			*syn*		
	ΔE_{RC}	$\Delta E^{\ddagger}$	ΔE_{rxn}	ΔE_{RC}	$\Delta E^{\ddagger}$	ΔE_{rxn}
**Propyne**	–2.83	13.42	–12.94	–2.75	11.79	–10.92
**2-butyne**	–3.01	15.81	–6.93	–	–	–
**COT**	–3.01	4.59	–14.33	–	–	–
**DIBO**	–2.65	4.06	–9.37	–	–	–
**BCN**	–3.10	2.11	–12.73	–	–	–
**HO-COT**	–4.93	2.31	–15.31	–8.86	6.82	–10.97
**DIFO**	–2.11	0.21	–15.94	–1.59	1.65	–14.43
**N-COT**	–2.94	2.89	–15.77	–4.34	3.30	–15.63
**O-COT**	–2.66	0.99	–19.52	–2.76	2.79	–18.09
**S-COT**	–2.72	5.84	–13.37	–2.93	6.02	–16.05

[a] Computed at ZORA-BP86/TZ2P.

Table S2. Gibbs free energies relative to reactants of the reactant complex ΔG_{RC} (kcal mol^{-1}), transition state $\Delta G^{\ddagger}$ (kcal mol^{-1}), and product ΔG_{rxn} (kcal mol^{-1}) for the retro-Cope reactions of propyne, 2-butyne and the original cyclooctyne **OCT** and functionalized cyclooctynes with along *anti*- and *syn*-pathways.[a]

Alkyne	*anti*			*syn*		
	ΔG_{RC}	$\Delta G^{\ddagger}$	ΔG_{rxn}	ΔG_{RC}	$\Delta G^{\ddagger}$	ΔG_{rxn}
**Propyne**	5.69	24.73	2.73	7.80	22.97	4.67
**2-butyne**	7.93	26.67	8.33	–	–	–
**COT**	8.66	15.84	2.17	–	–	–
**DIBO**	9.54	15.62	6.26	–	–	–
**BCN**	8.65	13.89	3.00	–	–	–
**HO-COT**	7.03	14.56	1.30	4.02	19.41	5.89
**DIFO**	9.80	12.96	0.54	9.62	13.85	2.69
**N-COT**	8.72	14.29	0.27	6.66	14.60	0.71
**O-COT**	8.98	13.14	–3.01	9.03	14.32	–1.52
**S-COT**	9.10	16.99	2.84	9.20	17.04	0.54

[a] Computed at ZORA-BP86/TZ2P.

Table S3. Activation energies $\Delta E^{\ddagger}$ (kcal mol^{-1}) for the retro-Cope reactions of propyne, 2-butyne and the original cyclooctyne **OCT** and functionalized cyclooctynes with **DHMA** along *anti*-pathways, computed at various levels of theory.

Alkyne	BP86/TZ2P[a]	BP86-D3(BJ)/TZ2P//BP86/TZ2P[b]	M06-2X/TZ2P//BP86/TZ2P[c]	COSMO(acetonitrile)-BP86/TZ2P//BP86/TZ2P[d]	DLPNO-CCSD(T)/def2-QZVPP//BP86/TZ2P[e]
**Propyne**	13.42	8.58	19.4	15.38	21.34
**2-butyne**	15.81	9.42	22.14	17.13	23.00
**COT**	4.59	–1.88	10.02	6.09	11.24
**DIBO**	4.06	–4.52	9.55	5.25	9.57
**BCN**	2.11	–4.53	6.03	3.86	7.65
**HO-COT**	2.31	–4.58	5.51	3.6	6.75
**DIFO**	0.21	–6.58	2.86	0.53	4.31
**N-COT**	2.89	–3.57	7.49	4.38	9.11
**O-COT**	0.99	–5.45	4.4	2.28	5.96
**S-COT**	5.84	–0.75	11.38	6.61	12.13

[a] Computed at ZORA-BP86/TZ2P.
[b] Energies computed at ZORA-BP86-D3(BJ)/TZ2P, with geometries computed at ZORA-BP86/TZ2P.
[c] Energies computed at ZORA-M06-2X/TZ2P, with geometries computed at ZORA-BP86/TZ2P.
[d] Energies computed at COSMO(acetonitrile)-ZORA-BP86/TZ2P, with geometries computed at ZORA-BP86/TZ2P.
[e] Energies computed at DLPNO-CCSD(T)/def2-QZVPP where the SCF convergence tolerance was set to tight, with geometries computed at ZORA-BP86/TZ2P.

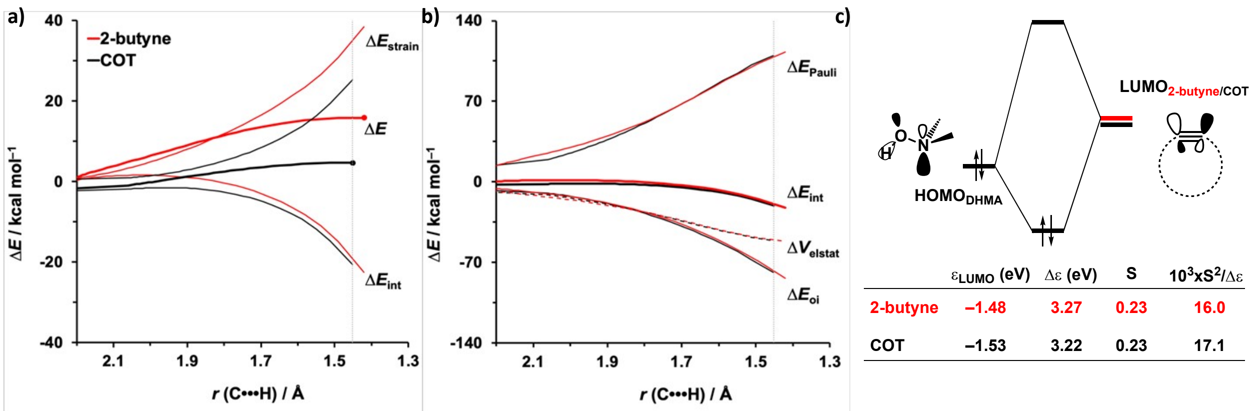

Fig. S3. a) Activation strain model and b) decomposition of strain energies for the retro-Cope reactions of 2-butyne and **COT** with **DMHA**. Transition states are indicated by dots. The energy terms along the IRC are projected on the length of the newly forming C···H bond and the vertical dotted line indicates the point at which the length of the C···H bond is 1.45 Å. c) Schematic diagrams with LUMO energies, energy gaps Δε, overlaps S, and values of $S^2/\Delta\varepsilon$ term associated with the key donor–acceptor interaction between $\text{HOMO}_{\textbf{DHMA}}$–$\text{LUMO}_{\text{alkyne}}$ along with the retro-Cope reactions between 2-butyne and **COT** with **DMHA**. All were computed at ZORA-BP86/TZ2P.

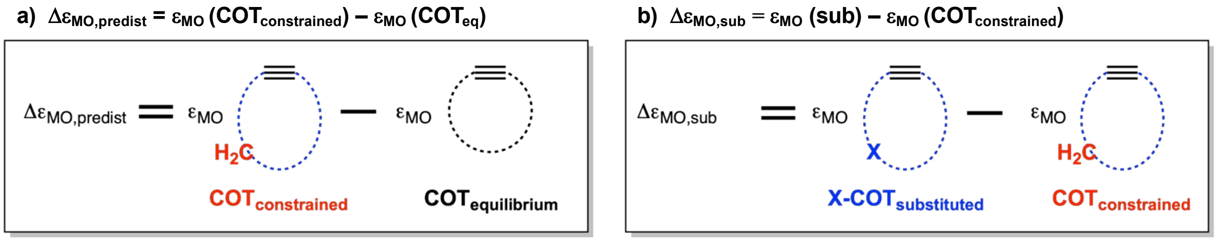

c)

Alkyne	HOMO / eV ε_{alkyne}	$\varepsilon_{constrained}$	$\Delta\varepsilon_{sub}$	$\Delta\varepsilon_{predist}$	LUMO / eV ε_{alkyne}	$\varepsilon_{constrained}$	$\Delta\varepsilon_{sub}$	$\Delta\varepsilon_{predist}$
**COT**	–5.91	–5.91	0.00	0.00	–0.42	–0.42	0.00	0.00
**DIBO**	–5.48	–5.69	0.21	0.22	–1.44	–0.66	–0.78	–0.24
**BCN**	–5.84	–5.81	–0.03	0.09	–0.61	–0.57	–0.04	–0.14
**HO-COT**	–6.09	–5.90	–0.18	0.00	–0.96	–0.40	–0.56	0.02
**DIFO**	–6.80	–5.91	–0.88	0.00	–1.40	–0.52	–0.88	–0.10
**N-COT**	–5.23	–5.97	0.74	–0.06	–0.72	–0.61	–0.11	–0.19
**O-COT**	–5.92	–5.95	0.03	–0.05	–1.05	–0.56	–0.49	–0.14
**S-COT**	–5.26	–5.80	0.53	0.11	–1.17	–0.78	–0.39	–0.36

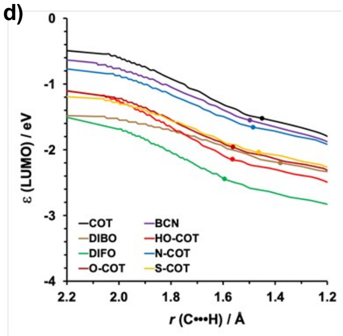

Fig. S4. A schematic representation of the formula that decomposes the change in orbital energies into a) a deformation ($\Delta\varepsilon_{deform}$) and b) a substituent ($\Delta\varepsilon_{sub}$) contribution. c) The π-HOMO and π*-LUMO energies (eV) for the cyclic alkynes in their equilibrium geometry (ε_{alkyne}) and the π-HOMO and π*-LUMO energies for cyclooctyne constrained in the equilibrium geometry of the alkynes. d) The π*-LUMO energies (eV) of the alkyne fragment associated with the retro-Cope reaction between strained alkynes and **DMHA** projected on the length of the newly forming C···H bond. The TSs are indicated by dots. Computed at ZORA-BP86/TZ2P.

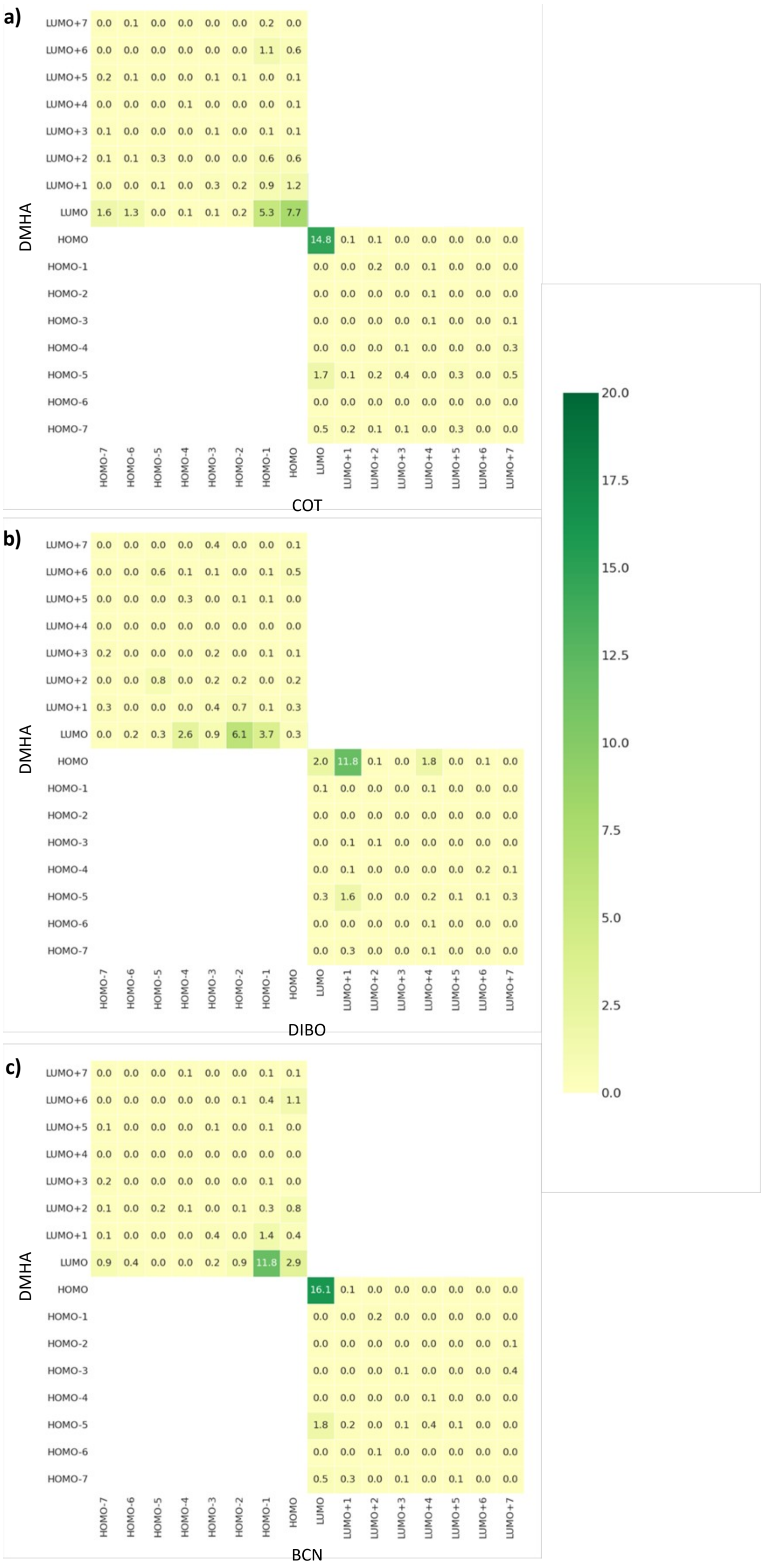

Fig. S5. Heatmaps for computed values of $S^2/\Delta\varepsilon$ associated with the donor–acceptor interactions along with the retro-Cope reactions of a) **COT**, b) **DIBO**, and c) **BCN** with **DMHA** at the consistent geometries where the length of C···H bond is 1.50 Å. All were computed at ZORA-BP86/TZ2P.

Electrostatic interaction of Cyclic Alkynes with Additional Predistortion

Electrostatic interactions are approximately equal for **COT** and **BCN**, whereas the **DIBO** shows a far less stabilizing ΔV_{elstat} term (Fig. 2c). Again, this is a direct consequence of the C···N bond forming distance in **DIBO** that lags behind that of **COT**. At a double consistent geometry, the ΔV_{elstat} terms are within a $\Delta\Delta V_{\text{elstat}} < 1.0$ kcal mol^{-1} for **COT**, **DIBO**, and **BCN** (Table S4). Therefore, we conclude that the deviation in Δr is decisive in setting the trend in electrostatic interactions. Specifically, it is the shorter bond-forming distance between the carbon in **COT** and **BCN**, and the nitrogen atom in **DMHA**, as compared to **DIBO**, gives rise to a more stabilizing ΔV_{elstat}.

Table S4. The energy decomposition analysis (in kcal mol^{-1}) of the retro-Cope reactions of the original **COT** and **DIBO** with **DMHA** at various consistent and double consistent geometries.[a]

		**Consistent Geometry (CG)**				**Double Consistent Geometry (DCG)**				**Difference between CG and DCG**			
**reactant**	r (C···H)	ΔE_{Pauli}[b]	ΔE_{elstat}[b]	ΔE_{oi}[b]	ΔE_{int}[b]	ΔE_{Pauli}[c]	ΔE_{elstat}[c]	ΔE_{oi}[c]	ΔE_{int}[c]	$\Delta\Delta E_{\text{Pauli}}$	$\Delta\Delta E_{\text{elstat}}$	$\Delta\Delta E_{\text{oi}}$	$\Delta\Delta E_{\text{int}}$
COT	1.85	42.7	–22.4	–22.3	–2.0	42.7	–22.4	–22.3	–2.0	0.0	0.0	0.0	0.0
COT	1.65	77.2	–38.3	–45.7	–6.7	77.2	–38.3	–45.7	–6.7	0.0	0.0	0.0	0.0
COT	1.50	101.4	–48.4	–67.8	–14.8	101.4	–48.4	–67.8	–14.8	0.0	0.0	0.0	0.0
COT	1.36	122.0	–54.5	–99.5	–32.1	122.0	–54.5	–99.5	–32.1	0.0	0.0	0.0	0.0
COT	1.18	164.6	–68.8	–160.5	–64.7	164.6	–68.8	–160.5	–64.7	0.0	0.0	0.0	0.0
DIBO	1.85	34.1	–16.5	–19.5	–1.9	46.5	–22.4	–24.4	–0.3	12.4	–5.9	–4.9	1.6
DIBO	1.65	66.1	–30.2	–40.9	–5.0	87.8	–39.3	–50.9	–2.5	21.8	–9.2	–10.0	2.6
DIBO	1.50	89.6	–39.4	–62.1	–11.9	109.2	–49.0	–70.8	–10.6	19.6	–9.7	–8.7	1.3
DIBO	1.36	111.8	–45.8	–94.0	–28.0	132.7	–55.1	–105.9	–28.3	20.9	–9.3	–11.9	–0.3
DIBO	1.18	154.9	–60.8	–156.4	–62.2	168.9	–68.1	–163.6	–62.8	14.0	–7.3	–7.3	–0.6

[a] Computed at ZORA-BP86/TZ2P.
[b] The energy terms at consistent geometries where the length of the C···H bond is equal to the r (C···H) given.
[c] The energy terms at double consistent geometry where both C···H and C···N bonds are equal to the archetypal **COT** at consistent geometry (r (C···H)).

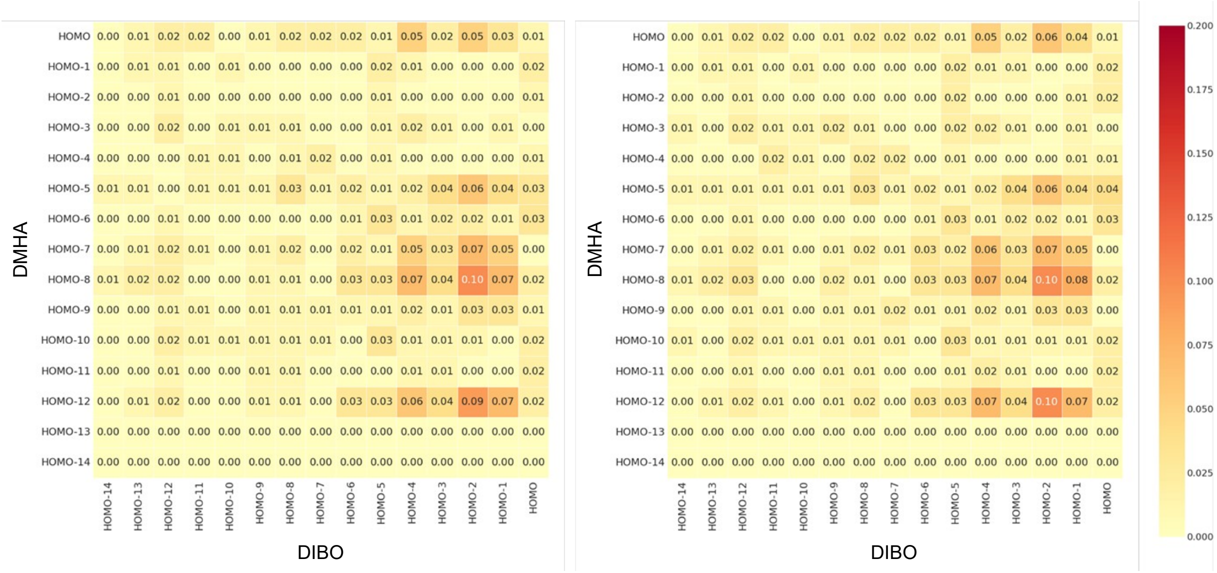

Fig. S6. Heatmaps for closed-shell orbital overlaps, S, between the filled FMOs of **DMHA** with the filled FMOs of a) **DIBO** at consistent geometry where C···H bond is 1.50 Å and b) **DIBO** in a double consistent geometry associated with the retro-Cope reactions along the anti-pathways. Computed at ZORA-BP86/TZ2P.

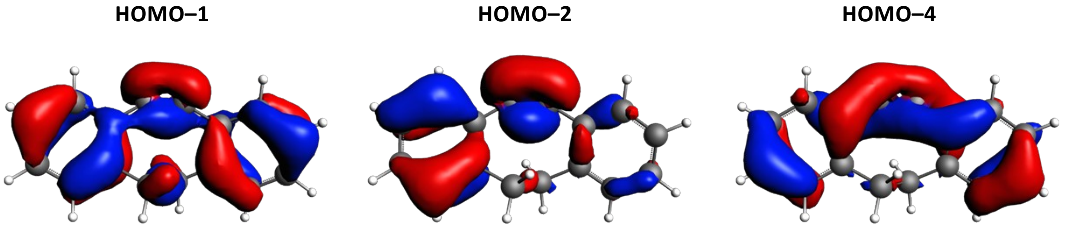

Fig. S7. The HOMO orbitals in **DIBO** that increase in Pauli repulsive overlap between the in-plane orbitals of **DMHA** going from CG to DCG at a C···H bond distance of 1.50 Å.

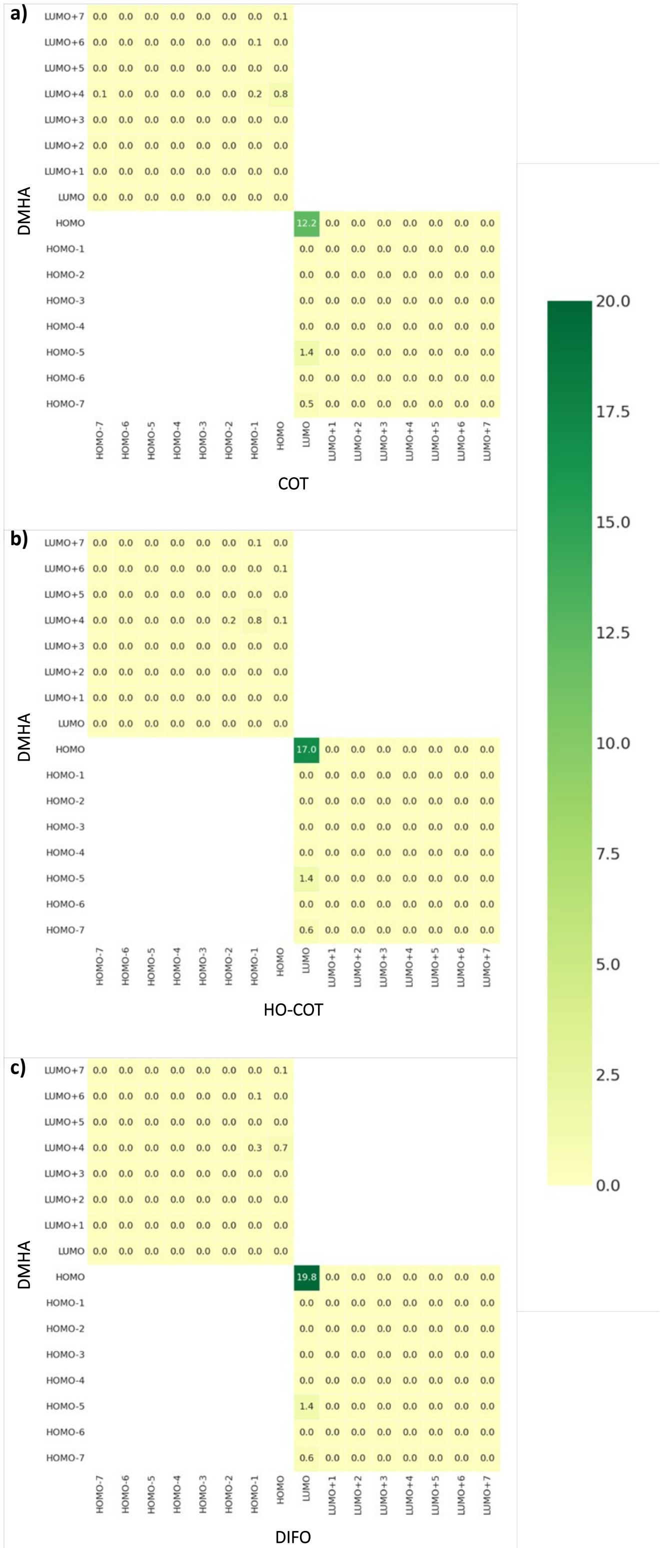

Fig. S8. Heatmaps for computed values of $S^2/\Delta\varepsilon$ associated with the donor–acceptor interactions along with the retro-Cope reactions of a) **COT**, b) **HO-COT**, and c) **DIFO** with **DMHA** along the *anti*-pathways at the consistent geometries where the length of C···H bond is 1.56 Å. All were computed at ZORA-BP86/TZ2P.

Electrostatic interaction of Cyclic Alkynes with Additional Predistortion

Comparing the electrostatic interactions terms at consistent geometry, we see that these are more stabilizing for **HO-COT** and **DIFO** than for **COT** (Fig. 4b). To understand if this is due to the more advanced (shorter) C···N bond forming distances than in **COT**, we determined the EDA terms at double consistent geometry (Table S5). At consistent geometry where the C···H and C···N bond forming distances are equal to the archetypal **COT**, we find that the ΔV_{elstat} terms **HO-COT** and **DIFO** are actually less stabilized, relative to **COT**. Therefore, we conclude that solely the deviation in Δr is decisive in setting the trend in electrostatic interactions. Specifically, it is the shorter bond forming distance between the carbon in **HO-COT** and **DIFO**, and the nitrogen atom in **DMHA**, as compared to **COT**, gives rise to a more stabilizing ΔV_{elstat}.

Table S5. The energy decomposition analysis (in kcal mol^{-1}) of the retro-Cope reactions of the original **COT, HO-COT** and **DIFO** with **DMHA** at various consistent and double consistent geometries.[a]

		**Consistent Geometry (CG)**				**Double Consistent Geometry (DCG)**				**Difference between CG and DCG**			
**reactant**	r (C···H)	ΔE_{Pauli}[b]	ΔE_{elstat}[b]	ΔE_{oi}[b]	ΔE_{int}[b]	ΔE_{Pauli}[c]	ΔE_{elstat}[c]	ΔE_{oi}[c]	ΔE_{int}[c]	$\Delta\Delta E_{\text{Pauli}}$	$\Delta\Delta E_{\text{elstat}}$	$\Delta\Delta E_{\text{oi}}$	$\Delta\Delta E_{\text{int}}$
COT	1.85	42.7	–22.4	–22.3	–2.0	42.7	–22.4	–22.3	–2.0	0.0	0.0	0.0	0.0
COT	1.65	77.2	–38.3	–45.7	–6.7	77.2	–38.3	–45.7	–6.7	0.0	0.0	0.0	0.0
COT	1.50	101.4	–48.4	–67.8	–14.8	101.4	–48.4	–67.8	–14.8	0.0	0.0	0.0	0.0
COT	1.36	122.0	–54.5	–99.5	–32.1	122.0	–54.5	–99.5	–32.1	0.0	0.0	0.0	0.0
COT	1.18	164.6	–68.8	–160.5	–64.7	164.6	–68.8	–160.5	–64.7	0.0	0.0	0.0	0.0
HO-COT	1.85	43.8	–23.0	–24.7	–3.8	41.8	–22.0	–23.6	–3.8	2.0	–1.0	–1.0	0.0
HO-COT	1.65	83.4	–41.9	–52.2	–10.6	75.6	–37.6	–48.4	–10.5	7.8	–4.2	–3.8	–0.2
HO-COT	1.50	114.5	–55.4	–80.4	–21.3	99.4	–47.6	–71.1	–19.3	15.0	–7.8	–9.3	–2.1
HO-COT	1.36	135.1	–62.1	–112.1	–39.1	120.2	–54.1	–103.0	–36.8	14.9	–8.0	–9.1	–2.3
HO-COT	1.18	177.2	–76.9	–172.2	–72.0	163.6	–69.2	–162.3	–67.9	13.5	–7.7	–9.9	–4.0
DIFO	1.85	42.4	–22.0	–25.3	–4.9	40.4	–21.1	–24.3	–5.0	2.0	–0.9	–1.0	0.1
DIFO	1.65	83.3	–41.4	–53.9	–12.1	73.1	–36.0	–49.2	–12.1	10.2	–5.4	–4.7	0.0
DIFO	1.50	115.9	–55.5	–82.9	–22.5	96.3	–45.3	–72.1	–21.1	19.6	–10.2	–10.8	–1.5
DIFO	1.36	136.5	–61.8	–114.7	–40.0	116.5	–51.0	–103.9	–38.4	20.0	–10.8	–10.8	–1.6
DIFO	1.18	176.8	–75.0	–174.8	–73.0	158.7	–64.8	–163.4	–69.5	18.1	–10.2	–11.4	–3.5

[a] Computed at ZORA-BP86/TZ2P.
[b] The energy terms at consistent geometries where the length of the C···H bond is equal to the r (C···H) given.
[c] The energy terms at double consistent geometry where both C···H and C···N bonds are equal to the archetypal **COT** at consistent geometry (r (C···H)).

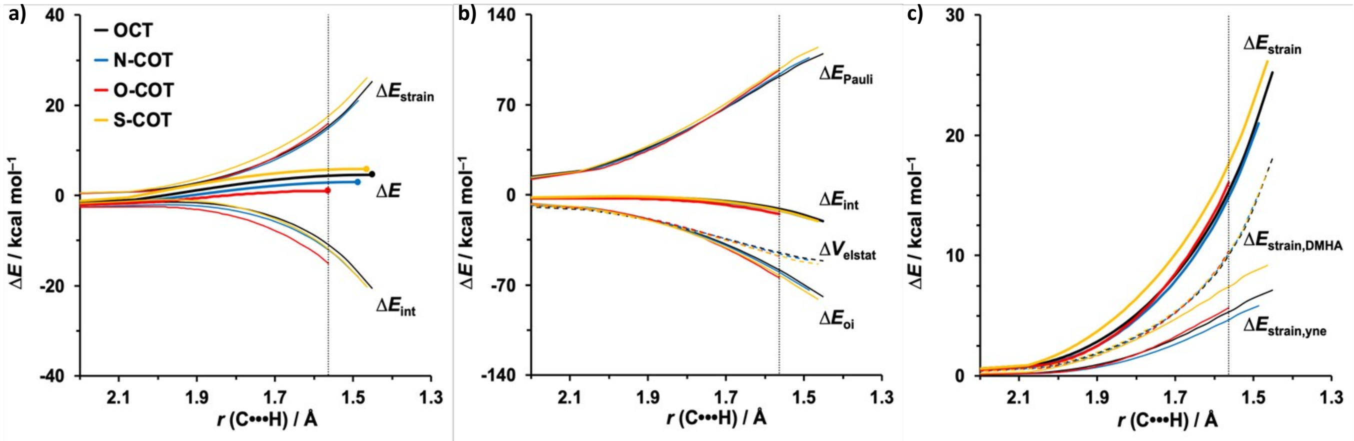

Fig. S9. a) Activation strain model, b) energy decomposition analyses and c) decomposition of strain energies for the retro-Cope reactions of **COT**, **N-COT**, **O-COT** and **S-COT** with **DMHA**. Transition states are indicated by dots. The energy terms along the IRC are projected on the length of the newly forming C···H bond and the vertical dotted line indicates the point at which the length of the C···H bond is 1.56 Å. All were computed at ZORA-BP86/TZ2P.

Table S6. The structural deformation of key angles (in °) and bonds distances (in Å) from equilibrium geometries to consistent geometries, where the lengths of the C···H bond are 1.56 Å, associated with the retro-Cope reactions of **COT**, **N-COT**, **O-COT** and **S-COT** with **DMHA** along the *anti*-pathways. Computed at ZORA-BP86/TZ2P.

	**DMHA**			**Y-COT**		
**Y**	Δ*r* (C···H)	Δ*r* (N···O)	Δ∠ (H–O–N)	Δ*r* (C–C)	Δ∠ (C–C≡C)	Δ∠ (C≡C–C)
**CH_2**	0.12	–0.09	–3.72	0.04	–13.92	–2.89
**NH**	0.12	–0.09	–3.43	0.04	–11.86	–3.85
**O**	0.12	–0.09	–3.40	0.04	–11.60	–6.00
**S**	0.12	–0.09	–3.72	0.04	–17.19	–6.39

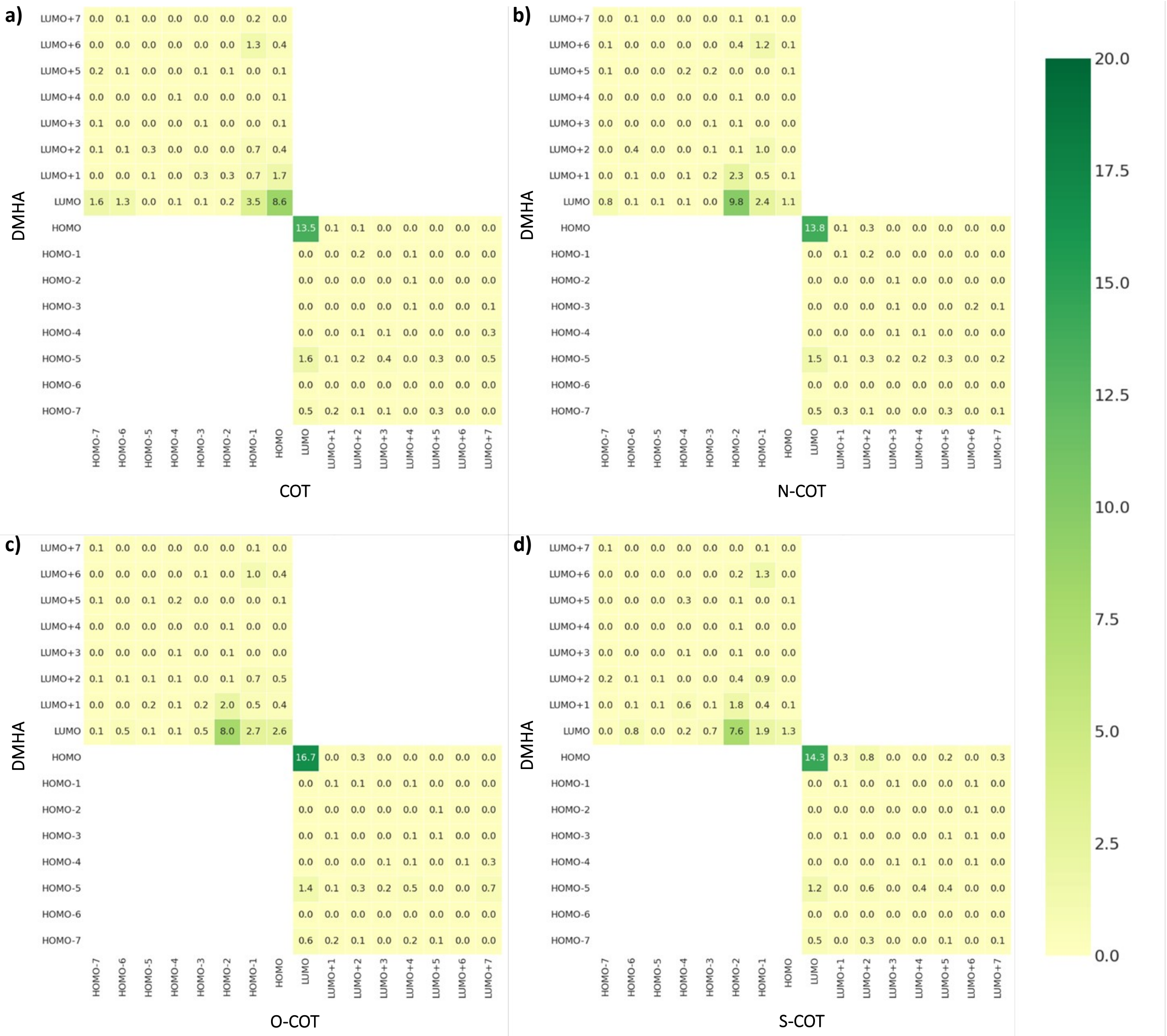

Fig. S10. Heatmaps for computed values of $S^2/\Delta\varepsilon$ associated with the donor–acceptor interactions along with the retro-Cope reactions of a) **COT**, b) **N-COT**, c) **O-COT** and d) **S-COT** with **DMHA** along the *anti*-pathways at the consistent geometries where the length of C···H bond is 1.56 Å. All were computed at ZORA-BP86/TZ2P.

Effect of Endocyclic Propargylic Substitution on the Electrostatic Interaction

Similar to the trends in the systems presented above, we suggest that the decrease in Δr in **Y-COT** establishes a more stabilizing electrostatic interaction between the two isolated deformed fragments. Specifically, from our EDA at double consistent geometry we see that the ΔV_{elstat} terms are within a $\Delta\Delta V_{elstat} < 1.0$ kcal mol^{-1} for **Y-COT** (Table S7). Therefore, we say that the more stabilizing electrostatic interaction is principally caused by the decreased distance between the terminal carbon of **Y-COT** and the nitrogen atom in **DMHA**, going from **COT** to **N-COT** to **O-COT**, resulting in a more stabilizing ΔV_{elstat}. Additionally, the VDD charges of key atoms of the isolated deformed fragments at a consistent geometry, where the C···H bond length is 1.71 Å (Fig. 7c), suggest that the attractive electrostatic interaction between the positively charged hydrogen of **DMHA** (+162 millielectrons) with the negatively charged carbon (C_2) closest to the endocyclic propargylic position in cyclooctyne (–115 millielectrons). Here, the electronegative substituents at the endocyclic propargylic position cause an accumulation of the electron density at the C_2 with the VDD charges going from –115 to –135 and –132 millielectrons for **COT**, **N-COT**, and **O-COT**.

Table S7. The energy decomposition analysis (in kcal mol^{-1}) of the retro-Cope reactions of the original **COT, N-COT** and **O-COT** with **DMHA** at various consistent and double consistent geometries.[a]

		Consistent Geometry (CG)				Double Consistent Geometry (DCG)				Difference between CG and DCG			
**reactant**	r (C···H)	ΔE_{Pauli}[b]	ΔE_{elstat}[b]	ΔE_{oi}[b]	ΔE_{int}[b]	ΔE_{Pauli}[c]	ΔE_{elstat}[c]	ΔE_{oi}[c]	ΔE_{int}[c]	$\Delta\Delta E_{Pauli}$	$\Delta\Delta E_{elstat}$	$\Delta\Delta E_{oi}$	$\Delta\Delta E_{int}$
COT	1.85	42.7	–22.4	–22.3	–2.0	42.7	–22.4	–22.3	–2.0	0.0	0.0	0.0	0.0
COT	1.65	77.2	–38.3	–45.7	–6.7	77.2	–38.3	–45.7	–6.7	0.0	0.0	0.0	0.0
COT	1.50	101.4	–48.4	–67.8	–14.8	101.4	–48.4	–67.8	–14.8	0.0	0.0	0.0	0.0
COT	1.36	122.0	–54.5	–99.5	–32.1	122.0	–54.5	–99.5	–32.1	0.0	0.0	0.0	0.0
COT	1.18	164.6	–68.8	–160.5	–64.7	164.6	–68.8	–160.5	–64.7	0.0	0.0	0.0	0.0
N-COT	1.85	42.5	–22.4	–22.9	–2.8	42.7	–22.6	–22.9	–2.8	–0.2	0.2	0.0	0.0
N-COT	1.65	77.3	–38.6	–46.3	–7.6	77.2	–38.5	–46.3	–7.6	0.1	–0.1	0.0	0.0
N-COT	1.50	104.3	–49.9	–71.4	–17.0	99.1	–47.5	–70.1	–18.5	5.3	–2.5	–1.3	1.6
N-COT	1.36	124.6	–56.3	–102.2	–33.8	122.0	–54.8	–99.6	–32.4	2.6	–1.4	–2.6	–1.5
N-COT	1.18	168.1	–71.4	–163.1	–66.4	165.3	–69.6	–160.2	–64.5	2.8	–1.8	–2.9	–1.9
O-COT	1.85	41.5	–21.8	–23.4	–3.7	41.9	–22.1	–23.5	–3.7	–0.4	0.3	0.0	0.0
O-COT	1.65	78.9	–39.5	–49.4	–10.0	75.4	–37.7	–47.9	–10.2	3.6	–1.9	–1.4	0.2
O-COT	1.50	108.5	–52.2	–76.5	–20.2	99.1	–47.5	–70.1	–18.5	9.5	–4.8	–6.4	–1.7
O-COT	1.36	129.1	–58.8	–107.7	–37.4	132.4	–57.0	–110.5	–35.2	–3.3	–1.8	2.8	–2.3
O-COT	1.18	171.0	–73.0	–168.2	–70.2	159.1	–65.7	–170.6	–77.3	11.9	–7.3	2.4	7.1

[a] Computed at ZORA-BP86/TZ2P.
[b] The energy terms at consistent geometries where the length of the C···H bond is equal to the r (C···H) given.
[c] The energy terms at double consistent geometry where both C···H and C···N bonds are equal to the archetypal **COT** at consistent geometry (r (C···H)).

Table S8. The total electronic energies (kcal mol^{-1}), enthalpies (kcal mol^{-1}), Gibbs free energies (kcal mol^{-1}) relative to the spherical average-of-configuration atoms, the number of imaginary vibrational frequencies (and their wavelength), and cartesian coordinates (Å), for all stationary points (reactant complex RC, transition state TS, product P), computed at ZORA-BP86/TZ2P using AMS2021.

DMHA (*reactant*)
E = –1304.97
H = –1242.81
G = –1263.95
N_{imag} = 0

N	-1.33165200	0.84445200	-0.34305000
C	-2.62488600	1.48989700	-0.55845700
O	-1.61334300	-0.33846500	0.48370400
C	-0.44835400	1.68515500	0.46224200
H	-2.45754200	2.40070000	-1.14884700
H	-3.12650700	1.76120300	0.39004800
H	-3.27505400	0.81694300	-1.12812800
H	-1.28350600	-1.05846900	-0.08175600
H	0.49381100	1.15501300	0.63940800
H	-0.89711400	1.96134500	1.43549000
H	-0.23562000	2.59994100	-0.10698700

Propyne (*reactant*)
E = –906.52
H = –869.60
G = –887.94
N_{imag} = 0

C	-1.45412379	0.00017528	0.00008524
C	0.00000000	0.00000000	0.00000000
C	1.20883600	-0.00000000	-0.00000000
H	-1.84876703	0.51310530	0.88842930
H	-1.84908846	-1.02548520	0.00010645
H	-1.84889850	0.51311555	-0.88819678
H	2.27773821	0.00002691	0.00001317

2-butyne (*reactant*)
E = –1288.53
H = –1233.75
G = –1254.20
N_{imag} = 0

C	-1.45624505	-0.00006439	0.00000000
C	0.00000000	0.00000000	0.00000000
C	1.21137154	0.00000000	0.00000000
C	2.66761658	0.00006439	0.00000000
H	-1.85559181	0.88521718	0.51590000
H	-1.85554219	-0.88942588	0.50880000

H	-1.85556935	0.00404748	-1.02460000
H	3.06691282	0.88542463	-0.51590000
H	3.06696491	-0.88931840	-0.50880000
H	3.06693775	0.00415496	1.02460000

COT (*reactant*)

E = –2600.02

H = –2485.07

G = –2510.79

N_{imag} = 0

C	-0.69354500	1.36871400	-0.35985700
C	0.69384400	1.36862900	0.35978100
C	-0.60522300	-1.48288400	-0.03639600
C	1.85526100	0.56859500	-0.28119500
C	0.60487800	-1.48301400	0.03689700
C	1.94700200	-0.92776800	0.13400400
H	-0.56471100	1.04644500	-1.40552800
H	0.56493800	1.04660000	1.40551700
H	1.76441500	0.62087000	-1.37691000
H	-1.02957200	2.41472600	-0.41400700
H	1.03010900	2.41457600	0.41372000
H	2.81177500	1.04915000	-0.02331100
H	2.31751600	-1.00956800	1.16910600
C	-1.85514300	0.56907300	0.28128000
H	-2.81154800	1.04979300	0.02329700
H	-1.76428700	0.62155100	1.37698500
C	-1.94722300	-0.92735400	-0.13361300
H	-2.31775900	-1.00928200	-1.16869800
H	-2.67680900	-1.45201100	0.50149700
H	2.67647000	-1.45272000	-0.50099700

DIBO (*reactant*)

E = –4225.85

H = –4079.11

G = –4111.01

N_{imag} = 0

C	-0.55729400	1.06963000	-0.90635700
C	0.70845400	1.36931600	-0.00803600
C	-0.44433400	-1.35710000	0.40684600
C	1.97012000	0.59852400	-0.33485300
C	0.75639500	-1.38534300	0.18014600
C	2.00628600	-0.82239900	-0.17690600
H	-0.26188400	0.35349400	-1.68732300
H	-3.04073100	2.20630600	-0.63220300
C	-1.77790000	0.52199300	-0.19721600
H	-0.84602500	1.99394400	-1.42391900

H	-5.05538500	1.24770100	0.44857300
C	-1.73007600	-0.76293600	0.42882600
C	3.19376200	-1.53387500	-0.41509900
C	4.34298700	-0.86038500	-0.82204500
C	4.31401600	0.52511600	-0.99888200
C	3.13681600	1.24010000	-0.75689400
H	3.19991900	-2.61537700	-0.28456300
H	5.26187500	-1.41685100	-1.00621700
H	5.21158000	1.05344600	-1.32070600
H	3.12736400	2.32382300	-0.88706000
C	-2.87846700	-1.30343800	1.03053900
C	-4.07074500	-0.58342700	1.03448600
C	-4.12401100	0.68127200	0.44337700
C	-2.98603200	1.22213600	-0.16323600
H	-2.82054100	-2.28568900	1.49805400
H	-4.95906400	-1.00668900	1.50315200
H	0.93514800	2.44191100	-0.06920400
H	0.44158200	1.16564200	1.03949500

BCN (*reactant*)

E = –2783.35

H = –2664.85

G = –2690.81

N_{imag} = 0

C	0.00557600	-0.98076900	-0.82309000
C	0.38789500	0.49460900	-0.92810000
C	-2.54086200	-0.08202500	0.25386000
C	0.12266600	1.58073600	0.10132000
C	-2.23693900	1.08958100	0.17033000
C	-1.30569100	2.20097200	0.01022000
H	0.25645300	1.18364100	1.11794000
H	0.86964400	2.38172000	-0.02741000
H	-1.42942800	2.97425000	0.78318000
H	-1.44541000	2.70125500	-0.96156000
C	-0.71589300	-1.65594100	0.33159000
H	-0.37230300	-1.24184500	1.29053000
H	-0.45355500	-2.72705500	0.33616000
C	-2.26828400	-1.51503700	0.27432000
H	-2.72367100	-2.02233800	1.13804000
H	-2.66536600	-2.00915100	-0.62699000
C	1.45422100	-0.55713800	-0.70960000
H	2.14989200	-0.79461200	-1.51342000
H	-0.26385800	-1.41182800	-1.79126000
H	0.32724500	0.86934400	-1.95360000
H	1.89447000	-0.60021200	0.28791000

HO-COT (*reactant*)

E = –2746.95

H = –2628.70

G = –2656.35

N_{imag} = 0

C	-1.44668300	1.21288600	-0.41825000
C	-0.19087200	1.66085700	0.39669600
C	-0.47149600	-1.44781200	0.03467300
C	1.20776000	1.25203500	-0.12611600
C	0.67451000	-1.08162900	0.19174800
C	1.75271100	-0.11322800	0.38073600
H	-1.14341800	0.94437200	-1.44291600
H	-0.29739100	1.33562300	1.44407300
H	1.19356000	1.21314300	-1.22732200
H	-2.09355400	2.09568900	-0.52595900
H	-0.20193000	2.75985300	0.43119600
H	1.95040100	2.01403100	0.15310700
H	1.99218500	-0.02983000	1.45282800
C	-2.33794100	0.08777300	0.16275200
H	-3.37176400	0.22417900	-0.18966300
H	-2.36615700	0.18103000	1.25891000
C	-1.90413600	-1.36275200	-0.19663600
H	-2.12607700	-1.57003400	-1.25623500
O	3.00485900	-0.44810600	-0.23770200
H	-2.47952700	-2.09061700	0.39445200
H	2.80705000	-0.75608600	-1.13952000

DIFO (*reactant*)

E = –2618.13

H = –2512.13

G = –2540.37

N_{imag} = 0

C	-1.68968700	1.19939800	-0.40153700
C	-0.33470800	1.60705500	0.25936800
C	-0.75998200	-1.47812700	-0.05702200
C	0.97633800	1.15767000	-0.42430600
C	0.42007600	-1.20185900	-0.05253200
C	1.50109600	-0.22685300	-0.01669000
H	-1.51928600	0.90867200	-1.45048600
H	-0.31897600	1.28890900	1.31254700
H	0.85357400	1.14218700	-1.51658900
H	-2.31277200	2.10390800	-0.44638100
H	-0.30093100	2.70517500	0.28687400
H	1.78750900	1.86431600	-0.19608100
C	-2.54309000	0.11492200	0.30081600
H	-3.60620500	0.28020400	0.07126000

H	-2.43635300	0.22154300	1.39036900
C	-2.20407700	-1.35505400	-0.08628600
H	-2.57424800	-1.57637400	-1.10018000
H	-2.70350200	-2.05837300	0.59581500
F	2.56131400	-0.57316700	-0.84406200
F	2.04049700	-0.15842900	1.26833500

N-COT (*reactant*)

E = –2486.78

H = –2378.84

G = –2404.45

N_{imag} = 0

C	0.14306700	1.90343100	-0.61209200
H	-0.48024500	2.66834800	-0.12833900
C	0.03306500	0.58466000	0.02312800
C	-0.05126900	-0.62666600	0.01649700
C	-0.14798600	-1.96204600	-0.55185100
C	0.29707000	-1.84599900	-2.03727900
C	-0.35396300	-0.68942800	-2.83503300
C	0.32730600	0.72451400	-2.82625900
N	-0.24874400	1.82698100	-2.03238300
H	1.18267300	2.26440000	-0.55085200
H	-1.18564500	-2.32987200	-0.49437800
H	0.47611200	-2.70042600	-0.02647600
H	0.07747000	-2.80311100	-2.53610400
H	1.39113900	-1.72597500	-2.06126900
H	-1.40912300	-0.58020600	-2.53568700
H	-0.37972100	-1.00897100	-3.88797800
H	0.32706400	1.08352800	-3.86653000
H	1.38439300	0.62590600	-2.53721000
H	-1.26780200	1.79162500	-2.09406000

O-COT (*reactant*)

E = –2362.77

H = –2262.78

G = –2288.17

N_{imag} = 0

C	0.13556300	1.89269100	-0.64907700
H	-0.53741300	2.63826800	-0.20544800
C	0.07665700	0.59188100	0.01097300
C	-0.00504500	-0.61844400	-0.00212400
C	-0.12832600	-1.95825600	-0.55052800
C	0.27158800	-1.85417000	-2.05188000
C	-0.35130900	-0.67082800	-2.83028700
C	0.35986900	0.71457800	-2.77147900
O	-0.30143700	1.74802400	-2.01420900

H	1.16399400	2.30059600	-0.61474500
H	-1.16646800	-2.31540100	-0.45593900
H	0.50892300	-2.69496900	-0.03958400
H	-0.00713100	-2.79897400	-2.54316000
H	1.36856000	-1.78331200	-2.11212500
H	-1.40232900	-0.52548600	-2.53935300
H	-0.37205700	-0.96502600	-3.89068000
H	0.38975200	1.12876300	-3.78858700
H	1.40246100	0.59963000	-2.42825100

S-COT (*reactant*)
E = –2307.75
H = –2209.43
G = –2235.97
N_{imag} = 0

C	0.12139500	2.01610900	-0.41985300
H	-0.60154000	2.65954900	0.09954100
C	0.06431000	0.63179200	-0.01435700
C	0.02817600	-0.57957500	-0.05697200
C	-0.08197700	-1.93315000	-0.57900100
C	0.26118300	-1.89965500	-2.09624800
C	-0.38987800	-0.73774000	-2.88309800
C	0.41030800	0.59763500	-2.92403400
S	-0.36344500	2.11217900	-2.22181400
H	1.12364600	2.44724300	-0.28561700
H	-1.11095100	-2.30157300	-0.43689300
H	0.57844800	-2.64282800	-0.05857300
H	-0.04773600	-2.86053900	-2.53464900
H	1.35473900	-1.84464200	-2.20862300
H	-1.40066300	-0.54447900	-2.49726000
H	-0.52120200	-1.07274100	-3.92247200
H	0.57808000	0.89782400	-3.96736600
H	1.40296100	0.48415700	-2.46919300

Propyne (*anti*-RC)
E = –2214.32
H = –2113.94
G = –2146.20
N_{imag} = 0

C	0.00000000	0.00000000	0.00000000
C	1.21099427	0.00000000	0.00000000
C	-1.45414488	-0.01700837	0.00000000
N	3.82784822	1.77471811	-0.00000000
C	4.59963848	2.06355739	1.20845300
H	5.46797707	1.39151828	1.23156700
H	4.95178950	3.11143516	1.24092400

C	4.59963848	2.06355739	-1.20845300
H	5.46797707	1.39151828	-1.23156700
H	4.95178950	3.11143516	-1.24092400
O	2.71438848	2.72137495	-0.00000000
H	1.93371480	2.12640660	-0.00000000
H	3.97750409	1.86774524	-2.08854100
H	3.97750409	1.86774524	2.08854100
H	2.28928870	0.04366731	0.00000000
H	-1.85631911	0.48916016	0.88879100
H	-1.83277891	-1.04885528	0.00000000
H	-1.85631911	0.48916016	-0.88879100

2-Butyne (RC)

E = –2596.51

H = –2478.85

G = –2510.22

N_{imag} = 0

C	0.00000000	0.00000000	0.00000000
C	1.21335235	-0.00000000	-0.00000000
C	-1.45680384	-0.01738965	0.00140900
N	2.15140949	3.54924823	-0.16454501
C	2.31241519	4.40283120	1.01161825
H	3.36712670	4.70197387	1.08057328
H	1.68179983	5.31112555	0.96171500
C	2.44635816	4.28585908	-1.39241542
H	3.50349494	4.58379040	-1.37197040
H	1.81829509	5.19063621	-1.50142853
O	0.72463382	3.22685490	-0.23005909
H	0.72319836	2.24646473	-0.16472721
H	2.28036858	3.63103665	-2.25492144
H	2.04735871	3.83387553	1.90950702
C	2.67088241	0.00048705	0.00139364
H	-1.86309694	0.63300534	0.78897412
H	-1.83731832	-1.03375263	0.17681588
H	-1.86086503	0.33054383	-0.95984964
H	3.05352086	1.03183523	-0.02962573
H	3.06689159	-0.53929995	-0.87066653
H	3.06474646	-0.48533717	0.90553958

COT (RC)

E = –3908.00

H = –3730.79

G = –3766.08

N_{imag} = 0

C	-2.76373300	-1.07648400	0.65851300
C	-2.84829500	0.31132000	1.37303100

C	-0.46704200	0.00303600	-0.67025700
C	-3.00443700	1.58102000	0.49937800
C	-0.84628900	1.14400300	-0.50459000
C	-1.67604600	2.22518300	0.00714300
H	-3.46782100	-1.74732900	1.17217300
H	-3.72619900	0.28130500	2.03486400
H	-3.55619200	2.34620200	1.06667600
H	-3.14494900	-0.98375000	-0.37110500
H	-1.98090800	0.43430000	2.04108800
H	-3.62046700	1.33864000	-0.37986200
H	-1.88415100	2.99944100	-0.74599300
C	-1.39905500	-1.80960100	0.63495800
H	-0.86466000	-1.60348400	1.57466700
H	-1.57214200	-2.89632200	0.60578800
C	-0.46110900	-1.44845300	-0.55247300
H	0.54994600	-1.84483300	-0.38126900
H	-0.83381600	-1.90434900	-1.48401600
H	-1.16621200	2.72893200	0.84414600
H	1.59085000	0.97619400	-0.44067900
N	3.08202300	-0.20335200	-0.10817500
C	4.03705300	-0.43486300	-1.19048300
H	4.48948500	-1.42583800	-1.04775300
H	4.83895100	0.32803800	-1.21544800
H	3.50581800	-0.42380800	-2.14852300
C	3.75805900	-0.15737500	1.18663800
H	4.55171200	0.61391100	1.22090600
H	4.20699100	-1.14150800	1.37872200
H	3.02249000	0.05433100	1.97047500
O	2.55260000	1.14297300	-0.32628900

DIBO (RC)

E = –5533.47

H = –5324.45

G = –5365.42

$\boldsymbol{N}_{imag}$ = 0

C	-0.69118600	1.69821500	0.19890500
C	0.30327400	1.95075800	1.40189200
C	-0.72258900	-0.85255700	1.25006100
C	1.65710400	1.27715900	1.31695100
C	0.49786900	-0.80047900	1.31772200
C	1.75615700	-0.14858100	1.33597600
H	-0.16981800	1.09100200	-0.55578000
H	-3.24290400	2.68400900	-0.02539900
C	-2.00328600	1.01863700	0.53095600
H	-0.91518900	2.66155100	-0.27770500
H	-5.38328000	1.51790100	0.42570200

C	-2.01725600	-0.32655600	1.01609800
C	3.00779900	-0.78486400	1.32407600
C	4.17084600	-0.01968500	1.27923600
C	4.09041700	1.37485200	1.24040600
C	2.84462300	2.01018800	1.25834600
H	3.03757000	-1.87574100	1.34339300
H	5.14375300	-0.51109900	1.26974200
H	5.00088700	1.97328400	1.20165800
H	2.79385000	3.10038200	1.23903400
C	-3.23317500	-0.98502500	1.26056100
C	-4.43965300	-0.32353300	1.04493100
C	-4.43972600	0.99770300	0.59116800
C	-3.23163600	1.65558900	0.34013000
H	-3.21886600	-2.01144900	1.62488600
H	-5.38165800	-0.83737500	1.23542300
H	0.46318700	3.03232900	1.50198500
H	-0.18871100	1.62171500	2.32919000
H	0.27171000	-3.17335300	1.42725500
N	1.87163600	-4.25225700	1.43364800
C	2.16982600	-5.05919100	0.25126000
H	3.25767400	-5.20132200	0.19456900
H	1.67738100	-6.04967100	0.28312200
H	1.83678400	-4.52341800	-0.64428900
C	2.25516400	-4.95021500	2.65975300
H	1.76379100	-5.93739300	2.75281400
H	3.34449000	-5.09175300	2.65111000
H	1.98587300	-4.33430400	3.52467100
O	0.41323000	-4.14199400	1.48165200

BCN (RC)

E = –4091.42

H = –3910.65

G = –3946.11

N_{imag} = 0

C	2.45774200	1.15818900	0.72766800
C	2.24805600	-0.31184700	1.09025400
C	-0.40288100	1.49230800	1.08051100
C	1.12283700	-1.21639600	0.61518700
C	-0.56490500	0.32408600	1.37104600
C	-0.18985900	-1.08426700	1.44711900
H	0.88075400	-1.00557300	-0.43634300
H	1.46632200	-2.26318300	0.65728700
H	-0.97178500	-1.73785000	1.03483200
H	-0.02069500	-1.39464700	2.49031700
C	1.58394900	2.00718900	-0.18087800
H	1.22585100	1.41168600	-1.03287800

H	2.19425500	2.82459800	-0.59918000
C	0.34104600	2.62250700	0.53368700
H	-0.25161800	3.21339600	-0.17979200
H	0.65636200	3.31239400	1.33243600
C	3.37733200	0.09422400	0.16834500
H	4.40698000	0.03419500	0.51838400
H	2.88449700	1.73493900	1.55283600
H	2.56010400	-0.53711800	2.11375400
H	3.25082400	-0.14846100	-0.88794300
H	-4.89229900	-2.28506000	0.32866100
H	-5.64750500	-0.67250500	0.09172400
O	-3.42679300	0.62976600	-0.15260300
H	-4.58905000	-0.97887200	1.50554600
H	-2.56009000	0.72398400	0.30150500
N	-3.54606800	-0.82035600	-0.30520500
C	-3.72846800	-1.05224500	-1.73639900
H	-3.86159300	-2.13055000	-1.89901300
H	-4.60780000	-0.51530500	-2.14192400
H	-2.83205800	-0.72205300	-2.27281800
C	-4.74188600	-1.20282500	0.44411400

HO-COT (*anti*-RC)

E = –4056.85

H = –3875.66

G = –3913.27

$\boldsymbol{N}_{\text{imag}}$ = 0

C	3.26639900	-0.17734200	-0.07117700
C	0.50506400	-1.62741800	-0.49163400
C	2.25974200	0.90005900	0.39566900
C	0.43449400	-0.41501300	-0.48029200
C	0.92812500	0.96413500	-0.39232100
H	2.57985200	-1.55506500	1.49002500
H	3.23507300	-0.25728500	-1.16984600
H	2.00006200	0.74565500	1.45341300
H	4.18135500	-1.89498300	0.86636100
H	4.26861200	0.20969100	0.16226000
H	2.71868700	1.89726800	0.32380100
H	1.14070700	1.32136700	-1.41979200
C	2.63125000	-2.76076700	-0.32238800
H	3.08077600	-3.70971500	0.00749500
H	2.95823700	-2.60339900	-1.36123000
C	1.08509500	-2.94916900	-0.31717900
H	0.76030200	-3.37847400	0.64427900
O	0.05954000	1.88421900	0.27048800
H	0.78498300	-3.65971900	-1.10124400
H	-0.85684400	1.68079200	-0.02375800

H	-1.83531700	-0.18533300	-0.60192200
N	-3.50658100	0.29261500	0.26491600
C	-3.44238700	1.23430000	1.38097200
H	-3.50491500	2.28948100	1.05202200
H	-4.28478500	1.02028800	2.05199500
H	-2.50794200	1.08223400	1.93186100
C	-4.70760200	0.50214600	-0.54019900
H	-5.57926700	0.27895600	0.08928100
H	-4.79103900	1.53906200	-0.91788200
H	-4.69994600	-0.19041300	-1.38873900
O	-2.38157500	0.63075000	-0.62251600
C	3.16619800	-1.60476300	0.55893400

DIFO (*anti*-RC)

E = –3925.21

H = –3757.00

G = –3794.52

$\boldsymbol{N}_{\text{imag}}$ = 0

C	-2.19337900	-2.08904700	0.32872200
C	-1.52094700	-2.67579300	-0.95222000
C	-0.19889300	-0.05529200	0.12021900
C	-0.02351400	-3.05044900	-0.88680900
C	0.62396900	-0.70864200	-0.49035500
C	0.96244400	-1.92459400	-1.22555900
H	-3.13865500	-2.63067100	0.47475000
H	-2.05379100	-3.60279400	-1.20572300
H	0.19757900	-3.86216500	-1.59503800
H	-1.58050200	-2.32590200	1.21317500
H	-1.68708200	-2.00256400	-1.80660500
H	0.23901500	-3.41262500	0.11730200
F	2.25411700	-2.35849300	-0.96253300
C	-2.54662500	-0.58198300	0.33448300
H	-2.86600900	-0.28271800	-0.67464300
H	-3.40547300	-0.41011800	0.99990200
C	-1.40533000	0.37230000	0.79554600
H	-1.65649000	1.41881800	0.57533100
H	-1.26123800	0.30152100	1.88487900
F	0.92627700	-1.67282200	-2.59639600
H	2.08158500	0.95021500	0.03584100
N	1.56204500	2.46942800	1.07947600
C	2.06741600	2.61123500	2.44399100
H	1.33754600	3.18968400	3.02673500
H	3.04601400	3.12685900	2.47854800
H	2.17374100	1.61795600	2.89366000
C	1.44950200	3.76825600	0.41718800
H	2.41150200	4.31480100	0.39745900

H	0.70661200	4.37097600	0.95746100
H	1.10484900	3.61883600	-0.61187900
O	2.58507200	1.73359900	0.34895100

N-COT (*anti*-RC)
E = –3794.69
H = –3624.49
G = –3659.68
N_{imag} = 0

N	2.40059068	2.09909294	-2.66733896
C	2.36525430	1.79219761	-4.09593312
O	1.00803203	2.31918061	-2.27551526
C	3.08704697	3.36614507	-2.42041315
H	3.39719566	1.65480414	-4.44684611
H	1.88696021	2.59611737	-4.68807543
H	1.81216113	0.85934136	-4.25123608
H	0.87953802	1.65131654	-1.56656805
H	3.06141332	3.58710477	-1.34769471
H	2.62737739	4.20877857	-2.97185387
H	4.13423969	3.26039456	-2.73549608
C	-1.31773640	-0.65174468	0.00000000
H	-2.04259558	-0.15464064	0.65895143
C	0.00000000	0.00000000	0.00000000
C	1.21586686	-0.00000000	0.00000000
C	2.56403981	-0.54693170	0.00601694
C	2.43058561	-2.04692534	-0.38191693
C	1.33362882	-2.83538968	0.37525907
C	-0.12606672	-2.85862265	-0.20084085
N	-1.19076017	-2.05486844	0.43025191
H	-1.74702271	-0.62584300	-1.01433621
H	3.01276764	-0.44859127	1.00771520
H	3.22520199	-0.02420269	-0.69995088
H	3.40689880	-2.52874279	-0.21694256
H	2.23316242	-2.10612152	-1.46309915
H	1.29857313	-2.50759836	1.42710405
H	1.66496419	-3.88438309	0.40717909
H	-0.47477115	-3.90058330	-0.14204168
H	-0.10834036	-2.60238193	-1.27068899
H	-1.08428962	-2.08530696	1.44562212

O-COT (*anti*-RC)
E = –3670.40
H = –3508.14
G = –3543.14
N_{imag} = 0

N	2.28195927	2.07347919	-2.73714879

C	2.36212123	1.71311913	-4.15132807
O	0.85256208	2.18357212	-2.44045880
C	2.84136229	3.40438373	-2.50302872
H	3.42187738	1.65517624	-4.43455247
H	1.85461190	2.44985682	-4.80332927
H	1.90192394	0.73018497	-4.30053756
H	0.74620573	1.56111205	-1.68904449
H	2.72847240	3.66202024	-1.44424047
H	2.34844363	4.18224769	-3.11694532
H	3.91188510	3.37828912	-2.74868300
C	-1.28888936	-0.69140361	0.00000000
H	-1.99300430	-0.29667258	0.74392042
C	0.00000000	0.00000000	0.00000000
C	1.21471689	0.00000000	0.00000000
C	2.57312168	-0.51452126	-0.01693260
C	2.46686676	-1.99613733	-0.48541174
C	1.35071339	-2.83545341	0.18127259
C	-0.08655133	-2.77075392	-0.41688237
O	-1.07739247	-2.06719971	0.36121113
H	-1.76751666	-0.61797981	-0.99484260
H	3.01430925	-0.45925152	0.99087058
H	3.22374833	0.05585576	-0.69473546
H	3.44002456	-2.47705037	-0.30397891
H	2.31543727	-2.00071481	-1.57553647
H	1.28228892	-2.60837186	1.25569422
H	1.66550948	-3.88813196	0.11820522
H	-0.48532990	-3.79312697	-0.46507661
H	-0.06091018	-2.37474995	-1.44650725

S-COT (*anti*-RC)
E = –3615.44
H = –3454.87
G = –3490.82
N_{imag} = 0

N	2.58454291	2.04837421	-2.63224271
C	2.65272362	1.69913497	-4.05012004
O	1.16316768	2.24750179	-2.34270799
C	3.22540938	3.33816034	-2.37871411
H	3.70886224	1.57588300	-4.32598671
H	2.19899581	2.47415694	-4.69717962
H	2.13162122	0.74946613	-4.21384944
H	1.00142745	1.60337528	-1.61966392
H	3.12203152	3.59011759	-1.31758979
H	2.78679076	4.15281816	-2.98604222
H	4.29360616	3.24715937	-2.61869150
C	-1.37036892	-0.45638009	-0.00000000

H	-2.01301192	0.07490143	0.71467397
C	0.00000000	0.00000000	0.00000000
C	1.21418466	-0.00000000	0.00000000
C	2.59167955	-0.46512453	0.07415057
C	2.59937479	-1.99485013	-0.20808834
C	1.49392424	-2.79887351	0.51643920
C	0.13066336	-2.91959696	-0.22683810
S	-1.37695737	-2.23219810	0.57451107
H	-1.82976153	-0.38583332	-0.99590574
H	2.99413523	-0.26511453	1.08015858
H	3.23650522	0.06240377	-0.64383213
H	3.58691931	-2.38497060	0.08021713
H	2.50919398	-2.15169270	-1.29362085
H	1.32582268	-2.38507025	1.52076883
H	1.87277381	-3.81988526	0.67008653
H	-0.14066488	-3.97868237	-0.33367531
H	0.18938704	-2.51005070	-1.24371785

Propyne (*anti*-TS)
E = –2198.07
H = –2100.00
G = –2127.16
N_{imag} = 1, ν = –433.649 i cm^{-1}

C	0.00000000	-0.00000000	-0.00000000
C	1.25439729	0.00000000	-0.00000000
C	-1.25302161	-0.79015769	0.00003235
N	1.72224032	2.01173737	-0.00010215
C	2.43778278	2.35260977	1.23145607
H	3.39574449	1.81495370	1.25192902
H	2.62582730	3.43621070	1.29325024
C	2.43765458	2.35250971	-1.23176501
H	3.39559761	1.81482312	-1.25231809
H	2.62572384	3.43610078	-1.29364598
O	0.44902284	2.51094349	-0.00006351
H	-0.08908716	1.51196064	-0.00002931
H	1.82403687	2.03855952	-2.08098509
H	1.82427817	2.03869189	2.08076937
H	2.24979206	-0.41396723	0.00001051
H	-1.86519521	-0.54078416	0.87893583
H	-1.07666919	-1.87554145	0.00005119
H	-1.86522030	-0.54081561	-0.87886269

2-butyne (TS)
E = –2577.69
H = –2461.83
G = –2491.48

N_{imag} = 1, ν = –642.9501 i cm^{-1}

C	0.00000000	0.00000000	0.00000000
C	1.25806856	-0.00000000	0.00000000
C	-1.21661784	-0.85326113	-0.01286097
N	1.56707094	2.14212454	0.03251139
C	2.23795225	2.55060091	1.27020514
H	3.26532408	2.16325402	1.27776922
H	2.26524733	3.64871940	1.35926368
C	2.23744801	2.58844079	-1.19241693
H	3.26478587	2.20149592	-1.21235298
H	2.26463300	3.68877429	-1.24785937
O	0.25349838	2.50520742	0.03828783
H	-0.18684918	1.40810118	0.02147135
H	1.67939308	2.19795727	-2.04885826
H	1.68024453	2.13418297	2.11447903
C	2.60800038	-0.57224931	-0.00859550
H	-1.84112781	-0.65161362	0.86970348
H	-0.98306977	-1.92787957	-0.02885847
H	-1.84082065	-0.62543159	-0.88931483
H	3.18667958	-0.26344484	-0.89173995
H	2.54574647	-1.67231992	-0.02531429
H	3.18628773	-0.29043558	0.88387893

COT (TS)

E = –3900.40

H = –3724.73

G = –3758.90

N_{imag} = 1, ν = –501.381 i cm^{-1}

C	-2.52141400	-1.17294300	0.28396700
C	-2.98288600	0.21386200	0.79813500
C	0.35911900	-0.03103600	0.25495500
C	-2.47573700	1.48226500	0.08234300
C	0.03530100	1.17903500	0.36000200
C	-1.13689600	2.06924700	0.56606200
H	-3.35158300	-1.87066400	0.47108200
H	-4.08029000	0.22725600	0.71521800
H	-3.23687000	2.27001100	0.20324700
H	-2.40779700	-1.13886200	-0.81263000
H	-2.77015500	0.29045700	1.87808600
H	-2.40893000	1.28671100	-1.00009100
H	-0.97328000	3.03710700	0.06631500
C	-1.26516700	-1.80626200	0.91562700
H	-1.21196800	-1.52684500	1.97947800
H	-1.37080800	-2.90220100	0.88796800
C	0.08743500	-1.47218000	0.24675900
H	0.87946000	-2.02931500	0.77272800

H	0.08406500	-1.85379100	-0.78956900
H	-1.20969300	2.30866200	1.64167900
H	1.36670600	1.73303700	0.14331300
N	2.46787600	0.21527400	-0.13043700
C	2.80683300	-0.27162300	-1.47044800
H	2.68121000	-1.36184200	-1.50551600
H	3.84653100	-0.01650900	-1.73310900
H	2.12732400	0.19977300	-2.18673800
C	3.27739100	-0.36353100	0.94476500
H	4.34152300	-0.10476400	0.82091700
H	3.17070100	-1.45633900	0.93811300
H	2.91701300	0.03600100	1.89751300
O	2.49001700	1.58157300	-0.08035700

DIBO (*anti*-TS)

E = –5526.76

H = –5319.37

G = –5359.34

N_{imag} = 1, ν = –635.6285 i cm^{-1}

C	-0.49754700	1.48729600	0.46596500
C	0.42192700	1.43572200	1.74043500
C	-0.53170700	-1.38986600	0.97076300
C	1.78870600	0.87813500	1.45675700
C	0.73731400	-1.27915000	0.99515900
C	1.93083000	-0.48935200	1.06590900
H	0.06522200	1.06683700	-0.38064300
H	-3.03445300	2.47016800	0.34560100
C	-1.79209800	0.73495800	0.60377800
H	-0.71121100	2.53358200	0.21072400
H	-5.17583500	1.23171100	0.53125800
C	-1.77796200	-0.67064000	0.82418100
C	3.21887000	-0.96670800	0.75199300
C	4.33750300	-0.14171200	0.82666900
C	4.19674200	1.18999900	1.22083800
C	2.92718800	1.68549300	1.52398200
H	3.33568700	-1.99780400	0.42967600
H	5.32058100	-0.53935500	0.57410100
H	5.06852000	1.84078900	1.28813700
H	2.81106300	2.72869900	1.82381000
C	-3.00428600	-1.35783300	0.92305200
C	-4.21890700	-0.68602800	0.81837700
C	-4.23013200	0.69549600	0.61402000
C	-3.02307400	1.39110000	0.51104300
H	-2.98672300	-2.43537900	1.08795100
H	-5.15552900	-1.23804700	0.89781700
H	0.52105300	2.44425900	2.16243000

H	-0.07303700	0.81663000	2.50233000
H	-0.64700600	-2.73896100	1.24489600
N	1.13364800	-3.43744100	1.40981200
C	1.77070300	-4.17012300	0.30474900
H	2.83379300	-3.91042900	0.24130500
H	1.67502600	-5.25485100	0.46824900
H	1.26698300	-3.89827700	-0.62764400
C	1.82494500	-3.55467800	2.70016500
H	1.90062200	-4.61015100	3.00717200
H	2.82833400	-3.11944700	2.61900300
H	1.24658100	-3.00168200	3.44550300
O	-0.16956600	-3.80058500	1.50847200

BCN (*anti*-TS)

E = –4086.21

H = –3906.79

G = –3940.87

$\boldsymbol{N}_{\text{imag}}$ = 1, ν = –359.8539 i cm^{-1}

C	1.89815200	1.02354700	0.81597800
C	1.71023400	-0.47942800	0.93963200
C	-1.02096400	1.20436000	-0.18082600
C	1.03183600	-1.33375900	-0.11120300
C	-1.08762500	-0.04556900	-0.08419400
C	-0.51056900	-1.39075300	0.03473700
H	1.27720500	-0.96633900	-1.11803000
H	1.42393700	-2.36241400	-0.05042700
H	-0.91731700	-2.06985700	-0.73189500
H	-0.76720400	-1.83792600	1.01049100
C	1.42810100	1.84361500	-0.36841000
H	1.54584600	1.26919200	-1.29962100
H	2.08034200	2.72790200	-0.46746100
C	-0.03771500	2.31653500	-0.25705100
H	-0.27699500	2.97110200	-1.11048500
H	-0.15365700	2.95671400	0.63448700
C	3.08913100	0.09635900	0.70319800
H	3.82167200	0.06921300	1.50867700
H	1.81976300	1.55816200	1.76637400
H	1.52133300	-0.82249900	1.96034200
H	3.51400200	-0.03769100	-0.29270700
H	-3.35230700	-1.87660500	1.33516100
H	-4.78786700	-0.80112800	1.33542300
O	-3.55856300	1.04628900	-0.13168500
H	-3.24500000	-0.23719500	2.04924800
H	-2.50088400	1.44519900	-0.17841200
N	-3.24465600	-0.28624000	-0.03491100
C	-3.72718300	-1.02154300	-1.20585000

H	-3.39223000	-2.06544900	-1.14484100
H	-4.82777000	-0.99757100	-1.26418000
H	-3.30792700	-0.55305900	-2.10148000
C	-3.68945200	-0.83530900	1.24789800

HO-COT (*anti*-TS)
E = –4049.61
H = –3869.99
G = –3905.74
N_{imag} = 1, ν = –187.669 i cm^{-1}

C	3.06716100	-0.59006800	0.08088900
C	-0.36180100	-0.34019100	-0.16898400
C	2.57262100	0.79216000	0.53993900
C	0.16219600	0.80149500	-0.19761400
C	1.48656900	1.45679500	-0.31985100
H	1.82338400	-1.74023700	1.45221600
H	3.24612100	-0.57387100	-1.00803900
H	2.21461200	0.74706300	1.57985100
H	2.95797300	-2.68360500	0.50469400
H	4.06019100	-0.72523200	0.53545500
H	3.42694300	1.48594400	0.53288500
H	1.79664900	1.38989500	-1.38245600
C	1.12970900	-2.29052700	-0.53868400
H	1.09211800	-3.39032100	-0.56995200
H	1.38232800	-1.96124400	-1.55831200
C	-0.29682300	-1.80512200	-0.19718500
H	-0.59487000	-2.22167000	0.78068900
O	1.47166200	2.85175700	0.06362300
H	-1.00025200	-2.22125900	-0.93671600
H	0.59248100	3.19635900	-0.16670700
H	-1.18826800	1.56842500	0.00148000
N	-2.41156400	0.15208700	0.12709900
C	-2.89128700	-0.32908200	1.42619200
H	-3.89535000	0.06675700	1.64654700
H	-2.92801900	-1.42592600	1.41550100
H	-2.19148300	0.00892900	2.19643000
C	-3.23443100	-0.26463100	-1.01186700
H	-3.28145400	-1.36063800	-1.04446700
H	-4.25560600	0.13986300	-0.92865200
H	-2.76888100	0.11158400	-1.92786300
O	-2.27553100	1.52426500	0.14392400
C	2.24244600	-1.85057300	0.43835100

DIFO (*anti*-TS)
E = –3922.89
H = –3755.12

G = –3791.36

$\boldsymbol{N}_{\text{imag}}$ = 1, ν = –121.3245 i cm^{-1}

C	-2.44088700	-1.15245900	0.15776400
C	-3.04465000	0.19231600	0.62719700
C	0.39213400	-0.01850900	0.38981300
C	-2.45050900	1.51022000	0.10285900
C	0.04497300	1.18336200	0.46531200
C	-1.14899800	1.99198900	0.74264700
H	-3.26112000	-1.88481800	0.17531600
H	-4.09806200	0.19937000	0.30996300
H	-3.17659600	2.31856700	0.27398200
H	-2.13726200	-1.07620000	-0.89955800
H	-3.06819100	0.22019600	1.72720300
H	-2.28043300	1.45557400	-0.98227900
F	-0.95176600	3.31364300	0.31955600
C	-1.28478600	-1.74835500	0.99262700
H	-1.35821800	-1.39177000	2.03076900
H	-1.39286200	-2.84284500	1.03114300
C	0.13869600	-1.45924400	0.46698200
H	0.87253000	-1.95302700	1.12388800
H	0.25971800	-1.91678700	-0.52987700
F	-1.33110700	2.08909300	2.12948300
H	1.46517000	1.78330300	0.05096600
N	2.43409600	0.19004600	-0.19692700
C	2.67655300	-0.37048600	-1.52979000
H	2.57764400	-1.46263800	-1.48699800
H	3.68485300	-0.11065700	-1.88872300
H	1.93154300	0.04175200	-2.21719800
C	3.33980200	-0.31010400	0.84242500
H	4.38329700	-0.03999600	0.61678800
H	3.25407700	-1.40249100	0.90381600
H	3.04788000	0.13798000	1.79707800
O	2.48009500	1.56912000	-0.24453500

N-COT (*anti*-TS)

E = –3788.86

H = –3620.14

G = –3654.11

$\boldsymbol{N}_{\text{imag}}$ = 1, ν = –411.14437 i cm^{-1}

N	1.60985056	2.12292626	0.10787807
C	2.28362907	2.61491815	-1.09586670
O	0.29595128	2.51249289	0.12676128
C	2.28110454	2.47224575	1.36223928
H	3.30671885	2.21786059	-1.13167320
H	2.32425747	3.71625491	-1.10451020
H	1.72294483	2.26712121	-1.96875233

H	-0.16797954	1.47631864	0.06651520
H	1.71222678	2.03405288	2.18773214
H	2.33089722	3.56542213	1.49466501
H	3.29914270	2.06095510	1.35714683
C	-1.16678604	-0.93624941	0.00000000
H	-1.99843749	-0.50490878	0.57656617
C	0.00000000	0.00000000	0.00000000
C	1.25686272	-0.00000000	0.00000000
C	2.56155383	-0.66849092	-0.03741852
C	2.44087668	-2.10552964	-0.59282422
C	1.53474849	-3.07067127	0.19620444
C	0.02158858	-3.14830883	-0.16115822
N	-0.94887581	-2.28798670	0.51639288
H	-1.54073250	-1.02173506	-1.03467957
H	3.00602504	-0.70298579	0.97301846
H	3.27724257	-0.11380744	-0.66514156
H	3.45913125	-2.52412993	-0.62872210
H	2.09221650	-2.05037393	-1.63622004
H	1.63868673	-2.88518565	1.27887046
H	1.93041404	-4.08600677	0.03494180
H	-0.29131708	-4.18459239	0.04578950
H	-0.10680730	-3.00381967	-1.24596787
H	-0.75239632	-2.25063796	1.51747949

O-COT (*anti*-TS)
E = –3666.75
H = –3505.39
G = –3538.98
N_{imag} = 1, ν = –189.76681 i cm^{-1}

N	1.64243108	2.11706296	0.11820876
C	2.31835918	2.61989044	-1.07955137
O	0.32913687	2.53685114	0.14084610
C	2.31324832	2.45353921	1.37640801
H	3.33759091	2.21396704	-1.12083423
H	2.36989609	3.72073795	-1.07338535
H	1.75483720	2.28879204	-1.95706419
H	-0.15187935	1.55414679	0.07997503
H	1.73963305	2.01498535	2.19832382
H	2.37389824	3.54526674	1.51505204
H	3.32682910	2.03189502	1.36972787
C	-1.14665005	-0.93073659	-0.00000000
H	-1.88683463	-0.64435823	0.76025229
C	0.00000000	-0.00000000	-0.00000000
C	1.25413913	0.00000000	-0.00000000
C	2.55560859	-0.67884129	-0.03237430
C	2.47929421	-2.01723642	-0.80302673

C	1.50384796	-3.07924278	-0.26495524
C	0.00905973	-2.96607647	-0.63726020
O	-0.82055529	-2.29287042	0.31495124
H	-1.65750999	-0.88197538	-0.98243500
H	2.90740521	-0.86668752	0.99699525
H	3.32628886	-0.05360270	-0.50908345
H	3.49410445	-2.44542878	-0.80525491
H	2.23206983	-1.80101856	-1.85465281
H	1.57705787	-3.15587760	0.83121827
H	1.84268902	-4.05108043	-0.65821775
H	-0.40211634	-3.98541930	-0.69209594
H	-0.10569916	-2.50920593	-1.63715815

S-COT (*anti*-TS)
E = –3606.88
H = –3447.81
G = –3482.93
$\boldsymbol{N}_{\text{imag}}$ = 1, ν = –438.34745 i cm^{-1}

N	1.60480584	2.09299373	0.07367551
C	2.29139894	2.56219162	-1.13424516
O	0.30018164	2.50057263	0.08263602
C	2.27920696	2.44610226	1.32795409
H	3.30778404	2.14858324	-1.16587164
H	2.35007812	3.66192842	-1.14771105
H	1.72473856	2.21859323	-2.00461047
H	-0.17474296	1.45437787	0.03786130
H	1.69750709	2.03043755	2.15578005
H	2.34729684	3.53964267	1.44158913
H	3.28928943	2.01636235	1.33323868
C	-1.17973976	-0.88381786	-0.00000000
H	-1.99866531	-0.47250773	0.60771068
C	-0.00000000	0.00000000	0.00000000
C	1.25973474	0.00000000	0.00000000
C	2.57635374	-0.65382891	0.00738442
C	2.59381838	-2.02687533	-0.70367791
C	1.80176834	-3.17621021	-0.05454779
C	0.30569541	-3.31306305	-0.40879845
S	-0.93732958	-2.56851496	0.71135096
H	-1.57706070	-0.98947200	-1.02190580
H	2.92339659	-0.78052283	1.04777464
H	3.32936803	-0.01895423	-0.48547528
H	3.65010296	-2.33317883	-0.75888540
H	2.25874670	-1.89186634	-1.74426734
H	1.91376694	-3.14526100	1.03998378
H	2.28150500	-4.11314040	-0.38063322
H	0.03742413	-4.37815948	-0.38536141

H	0.10391544	-2.96298166	-1.43215946

Propyne (*anti*-P)
E = –2224.43
H = –2122.96
G = –2149.16
N_{imag} = 0

C	0.00000000	-0.00000000	-0.00000000
C	1.32535282	-0.00000000	-0.00000000
C	-0.86212487	-1.22109441	-0.00000000
N	2.06351211	1.31592188	-0.00001639
C	2.94467295	1.33734705	1.23294895
H	3.61904709	0.47031400	1.25880680
H	3.50612719	2.27513953	1.19695283
C	2.94462341	1.33734290	-1.23301753
H	3.61899035	0.47030469	-1.25890769
H	3.50608425	2.27513133	-1.19704364
O	1.25160995	2.39504618	-0.00000350
H	-0.45436393	0.99685773	-0.00000283
H	2.26927382	1.33696915	-2.09164303
H	2.26935926	1.33696805	2.09160278
H	1.97099955	-0.87909252	0.00000108
H	-1.52192294	-1.22808933	0.88057920
H	-0.27746900	-2.14998305	-0.00000850
H	-1.52193199	-1.22807885	-0.88057115

2-butyne (P)
E = –2600.43
H = –2480.99
G = –2509.82
N_{imag} = 0

C	0.00000000	0.00000000	0.00000000
C	1.33136785	0.00000000	0.00000000
C	-0.90970903	-1.18488116	-0.00989076
N	1.96588880	1.41707439	0.01184244
C	2.82892135	1.54401616	1.24773000
H	3.63269138	0.79710022	1.26869608
H	3.23855600	2.55741020	1.22792012
C	2.83042620	1.56397321	-1.22083558
H	3.63443454	0.81768505	-1.25267811
H	3.23955805	2.57702680	-1.18433293
O	1.05392642	2.41308053	0.01940486
H	-0.43272153	1.00710677	0.00829975
H	2.16244191	1.46388713	-2.07980539
H	2.15988025	1.43033434	2.10404480
C	2.25973211	-1.17484977	-0.01013483

H	-1.57060422	-1.16717292	0.87007218
H	-0.38364176	-2.14815830	-0.01760120
H	-1.57020163	-1.15279185	-0.88970789
H	2.90971828	-1.19474725	-0.89694616
H	1.67747506	-2.10419168	-0.01645501
H	2.91214391	-1.20830431	0.87449547

COT (P)
E = –3919.32
H = –3740.07
G = –3772.57
$\boldsymbol{N}_{\text{imag}}$ = 0

C	-2.34866600	-1.35009300	0.80270800
C	-2.71945800	0.11293700	1.08940900
C	0.61941100	0.29132900	-0.02909700
C	-2.46907100	1.11407500	-0.06453800
C	0.12523000	1.50332400	0.20461200
C	-1.26853200	2.04930400	0.12883400
H	-2.79436100	-1.97881900	1.58963600
H	-3.78931900	0.13185600	1.34376200
H	-3.35378300	1.75893100	-0.17169300
H	-2.83406700	-1.65888400	-0.13923300
H	-2.19125900	0.45534600	1.99445300
H	-2.38782100	0.57581400	-1.02063700
H	-1.27299900	2.80438200	-0.67781800
C	-0.85699500	-1.69645800	0.72887800
H	-0.38045100	-1.47182900	1.69498900
H	-0.77711600	-2.78579200	0.59100300
C	-0.05690300	-1.00166400	-0.40756300
H	0.69434400	-1.71089300	-0.77710700
H	-0.72225200	-0.82944500	-1.26623400
H	-1.43336300	2.63959900	1.04794000
H	0.91618700	2.21133900	0.49057700
N	2.18229100	0.23431200	0.14905300
C	2.78952900	-0.10593200	-1.19490700
H	2.41003600	-1.05095700	-1.60289600
H	3.87054500	-0.15771600	-1.03772700
H	2.54939400	0.73127000	-1.85409900
C	2.51382000	-0.84447800	1.15360300
H	3.60492800	-0.87729800	1.21986400
H	2.11093700	-1.82320800	0.86589800
H	2.09670500	-0.51013600	2.10631900
O	2.72404100	1.39405100	0.57312800

DIBO (P)
E = –5540.19

H = –5329.38
G = –5368.70
N_{imag} = 0

C	-0.67747200	1.31547900	2.24969900
C	0.66294800	0.68688800	2.68715300
C	-0.74231400	-1.77250800	1.40815400
C	1.58795100	0.39351300	1.53341700
C	0.58509300	-1.87375300	1.35358400
C	1.53320000	-0.85258900	0.86197200
H	-0.48092700	2.35285800	1.94019700
H	-2.30542500	2.61833300	0.67668500
C	-1.50817400	0.67625200	1.14291800
H	-1.31586600	1.39172400	3.14680300
H	-3.92741200	1.82745600	-1.00703900
C	-1.58404700	-0.70344100	0.83542200
C	2.34063400	-1.04779400	-0.27415400
C	3.21428800	-0.05755000	-0.71835100
C	3.29647600	1.15470600	-0.03093500
C	2.48046800	1.37266300	1.07818600
H	2.25917000	-1.97530500	-0.83852500
H	3.82274900	-0.23004400	-1.60614900
H	3.98368600	1.93198900	-0.36555000
H	2.52987500	2.32737100	1.60546900
C	-2.54833600	-1.14065300	-0.09762400
C	-3.38649700	-0.25495200	-0.76424500
C	-3.28636100	1.11073800	-0.49343300
C	-2.36455400	1.55170600	0.45091000
H	-2.61644700	-2.20949600	-0.30425700
H	-4.11036800	-0.62747100	-1.48910200
H	1.14817900	1.39724700	3.37248800
H	0.46730500	-0.22715400	3.25923200
H	-1.21788600	-2.66141700	1.85041000
N	1.12567900	-3.20432000	2.01542500
C	1.81983500	-4.03559200	0.96151400
H	2.69239100	-3.51825400	0.54875200
H	2.11573000	-4.96196600	1.46184700
H	1.07752900	-4.25116900	0.18931600
C	2.12745600	-2.81299500	3.07675300
H	2.52409200	-3.74614700	3.48659800
H	2.92556500	-2.18753900	2.65948800
H	1.56925400	-2.28090200	3.85038600
O	0.14643600	-3.93875200	2.57115000

BCN (P)
E = –4101.05
H = –3918.31

G = –3951.76
N$_{imag}$ = 0

C	1.95527600	1.05430400	0.82046400
C	1.69060400	-0.42597200	0.97043500
C	-1.18827700	1.35215700	-0.17076200
C	0.89437500	-1.18588600	-0.06573900
C	-1.42251300	0.05152800	-0.02808300
C	-0.62091500	-1.20272000	0.19942200
H	1.09610300	-0.80088800	-1.07469100
H	1.23384800	-2.23441100	-0.06987600
H	-1.04598100	-2.01375100	-0.41112000
H	-0.76538000	-1.52811500	1.24467100
C	1.42989900	1.80975400	-0.37770100
H	1.53255400	1.20220900	-1.28872200
H	2.06295000	2.69706000	-0.54157300
C	-0.01782500	2.29250600	-0.23389800
H	-0.23215300	2.98321200	-1.06683600
H	-0.08885000	2.93372400	0.66445100
C	3.08852400	0.06505500	0.66670800
H	3.84428800	0.00418600	1.44814200
H	1.94942100	1.63130000	1.74824200
H	1.52703000	-0.77632800	1.99264200
H	3.47006500	-0.10168900	-0.34147500
H	-2.90037800	-1.96444900	1.23398300
H	-4.48734300	-1.11715700	1.13264900
O	-3.75566800	0.90145300	-0.11123000
H	-3.13838000	-0.36157400	2.03085300
H	-2.16436500	1.86855100	-0.24480500
N	-3.01723800	-0.22252700	-0.06152200
C	-3.32621800	-1.03788000	-1.29463800
H	-2.79427400	-1.99701100	-1.30949800
H	-4.40796600	-1.19921000	-1.29281400
H	-3.04519900	-0.41917900	-2.14984300
C	-3.40203600	-0.99124500	1.17725200

HO-COT (*anti*-P)
E = –4067.23
H = –3884.74
G = –3919.00
N$_{imag}$ = 0

C	2.95495800	-0.64060000	0.58502600
C	-0.68553000	-0.13330400	-0.23371400
C	2.46777800	0.81346200	0.62010600
C	0.05870100	0.96515900	-0.15271800
C	1.49801200	1.22233700	-0.50548900
H	1.16832100	-1.55511500	1.41518400

H	3.59036700	-0.78592300	-0.30489800
H	1.99597000	1.03867200	1.58860100
H	2.37960200	-2.69143600	0.84443800
H	3.61670100	-0.78659800	1.45228900
H	3.34017000	1.47755400	0.53265800
H	1.78203800	0.69498600	-1.43288500
C	1.11788000	-1.91788500	-0.73975800
H	1.15565200	-2.97228700	-1.05032600
H	1.63240800	-1.36442400	-1.53688100
C	-0.37029600	-1.52301700	-0.71278800
H	-0.89881400	-2.25355900	-0.07971600
O	1.70118900	2.63765100	-0.71124000
H	-0.78437800	-1.66786000	-1.72534300
H	0.93306900	2.97765700	-1.20162600
H	-0.50484500	1.81324800	0.26799800
N	-2.15704900	0.10904200	0.27065700
C	-2.37555100	-0.70746100	1.52551700
H	-3.40510000	-0.51328300	1.83805300
H	-2.21533400	-1.78035000	1.36171200
H	-1.68166200	-0.31309100	2.27131800
C	-3.10633200	-0.35086800	-0.81104000
H	-2.97718700	-1.41397400	-1.04833100
H	-4.11402200	-0.15357200	-0.43568100
H	-2.90751600	0.27590100	-1.68396400
O	-2.42006800	1.39973900	0.55751200
C	1.88125900	-1.74327000	0.59682100

DIFO (*anti*-P)
E = –3939.04
H = –3768.82
G = –3803.78
N_{imag} = 0

C	-1.77502800	-1.59030900	0.62506100
C	-1.23269200	-2.74916800	-0.22763400
C	0.41450700	0.61356700	0.08940000
C	0.25492900	-2.72240300	-0.60361100
C	1.14452000	-0.30458600	-0.53073100
C	0.77078600	-1.49479300	-1.36004000
H	-2.73039500	-1.92082400	1.05664700
H	-1.39651600	-3.69298700	0.31376300
H	0.47296100	-3.58584500	-1.24822700
H	-1.10573800	-1.41008900	1.48190000
H	-1.83249900	-2.81923200	-1.14749200
H	0.88130400	-2.82751500	0.29421500
F	1.93241300	-1.89010400	-2.02785500
C	-2.01353700	-0.27675100	-0.15768400

H	-1.98055000	-0.47877700	-1.23456900
H	-3.02793400	0.09364500	0.04882200
C	-1.05905900	0.88175000	0.17656900
H	-1.30581900	1.72895700	-0.48513300
H	-1.29683200	1.23156800	1.19322300
F	-0.12222200	-1.15558800	-2.38320800
H	2.22336800	-0.14869600	-0.36762400
N	1.34951500	1.64121900	0.85914900
C	1.05403000	1.54209200	2.33942300
H	0.01045000	1.77994400	2.57776700
H	1.73180900	2.24744700	2.82722100
H	1.30636200	0.52169500	2.63746200
C	1.02427100	3.02887300	0.35706800
H	1.69367600	3.70959800	0.88977300
H	-0.02526800	3.29350500	0.53333400
H	1.26085100	3.03587800	-0.70981500
O	2.66109300	1.40736700	0.67752500

N-COT (*anti*-P)
E = –3807.52
H = –3635.52
G = –3668.13
N_{imag} = 0

N	1.93486470	1.44967999	0.03933676
C	2.85106639	1.61810896	-1.15075650
O	1.00539160	2.42548265	0.00581950
C	2.72260056	1.59064346	1.32398868
H	3.64091673	0.85758619	-1.18257670
H	3.28014298	2.62065881	-1.06876938
H	2.21208193	1.56162494	-2.03504225
H	-0.41479718	1.01871610	0.01920771
H	1.99543277	1.50151826	2.13420963
H	3.14638092	2.59868929	1.31374041
H	3.51411098	0.83687351	1.41878899
C	-0.99225239	-1.13338396	0.00000000
H	-1.54577673	-1.08152763	0.95427341
C	-0.00000000	0.00000000	0.00000000
C	1.32861260	-0.00000000	0.00000000
C	2.31376190	-1.14144381	-0.04526897
C	2.40810762	-1.89868709	-1.39769354
C	1.49270861	-3.11901738	-1.54641463
C	-0.02315165	-2.87938169	-1.50278073
N	-0.57993581	-2.51490001	-0.19538579
H	-1.75364484	-0.90141910	-0.76514129
H	2.08108699	-1.85980249	0.75593835
H	3.31459182	-0.76555416	0.20228421

H	3.44464884	-2.24683518	-1.52412115
H	2.21704797	-1.19287358	-2.22016514
H	1.74879486	-3.86007291	-0.76825926
H	1.72638171	-3.60731453	-2.50639526
H	-0.51417587	-3.80395696	-1.85350612
H	-0.29693227	-2.09354978	-2.22402757
H	-0.00248997	-2.85575376	0.56993874

O-COT (*anti*-P)
E = –3687.26
H = –3523.05
G = –3555.13
N_{imag} = 0

N	1.93120069	1.44541005	0.04435916
C	2.87570072	1.61146163	-1.12315655
O	0.99981486	2.41978106	-0.01223044
C	2.69087569	1.59101898	1.34611842
H	3.66681128	0.85174295	-1.12988723
H	3.30005218	2.61533792	-1.03392838
H	2.26011564	1.55065647	-2.02364791
H	-0.43096322	1.01118583	0.01323635
H	1.94766380	1.49928443	2.14127231
H	3.11001779	2.60093626	1.34252526
H	3.48284456	0.84018166	1.45704020
C	-0.94413408	-1.16062147	-0.00000000
H	-1.43198813	-1.22491067	0.98675956
C	0.00000000	-0.00000000	-0.00000000
C	1.32942680	-0.00000000	-0.00000000
C	2.30343798	-1.15207882	-0.03332189
C	2.46640162	-1.85626712	-1.40974049
C	1.50415958	-3.02146319	-1.66982302
C	0.01959604	-2.68437553	-1.59012513
O	-0.37507683	-2.44532539	-0.22812681
H	-1.75487115	-0.96161152	-0.72888884
H	1.99323994	-1.89739904	0.71195951
H	3.29255136	-0.80146053	0.28835152
H	3.49267904	-2.24751619	-1.47816266
H	2.36653271	-1.11158781	-2.21387140
H	1.70416037	-3.83535798	-0.95484711
H	1.70715895	-3.43129190	-2.67240303
H	-0.57608783	-3.53251913	-1.96676422
H	-0.21411948	-1.80653964	-2.21536012

S-COT (*anti*-P)
E = –3626.09
H = –3463.71

G = –3497.08
N_{imag} = 0

N	1.92226608	1.45250168	0.01909414
C	2.85942309	1.60534175	-1.15585675
O	0.98560660	2.42158084	-0.04872375
C	2.68674713	1.62026875	1.31461372
H	3.65443085	0.84984425	-1.15534277
H	3.27913024	2.61251427	-1.08434794
H	2.23995788	1.52742128	-2.05235473
H	-0.40773263	1.02370486	-0.01068070
H	1.94746981	1.53833231	2.11445162
H	3.10409214	2.63080778	1.29431732
H	3.48060823	0.87259873	1.43286146
C	-1.00515304	-1.10543170	-0.00000000
H	-1.67565474	-0.96853899	0.86370541
C	0.00000000	-0.00000000	-0.00000000
C	1.32877070	-0.00000000	-0.00000000
C	2.31131467	-1.13909886	-0.04516869
C	2.46047456	-1.82678950	-1.43673723
C	1.76332870	-3.18509053	-1.57347339
C	0.23326113	-3.19175748	-1.51307858
S	-0.51306527	-2.86243555	0.13504585
H	-1.65508202	-1.00248615	-0.88483436
H	2.00438684	-1.89130288	0.69341306
H	3.29925282	-0.78859317	0.27895020
H	3.52998506	-1.98482249	-1.64011029
H	2.09645293	-1.14862065	-2.22292472
H	2.15080233	-3.87403197	-0.80625236
H	2.05166536	-3.61758552	-2.54643267
H	-0.13521648	-4.18782259	-1.79271570
H	-0.20252574	-2.47571508	-2.22504154

Propyne *(syn*-RC*)*
E = –2214.24
H = –2114.46
G = –2144.09
N_{imag} = 0

C	1.21055967	0.00000000	0.00000000
C	0.00000000	0.00000000	0.00000000
H	2.27977020	-0.01835717	0.00019798
N	-0.92911529	3.52745873	0.01860353
C	-1.21766634	4.31319307	-1.18065988
H	-2.29093297	4.54704961	-1.19124170
H	-0.64192312	5.25755155	-1.21272909
C	-1.18885554	4.30391330	1.23050932
H	-2.26167026	4.53701701	1.26889842

H	-0.61286344	5.24831229	1.25579828
O	0.51702834	3.29465699	0.00000000
H	0.57628166	2.31575574	-0.00024207
H	-0.92598443	3.70266650	2.10773525
H	-0.97642776	3.71859809	-2.06857679
C	-1.45412202	0.02252434	-0.00046923
H	-1.85414355	-0.49139797	0.88459948
H	-1.85329392	-0.47642922	-0.89444040
H	-1.81031271	1.06408942	0.00813575

HO-COT (*syn*-RC)

E = –4060.78

H = –3879.51

G = –3916.28

N_{imag} = 0

C	3.19089800	-0.73754700	-0.19337400
C	0.06479300	-1.03747200	-0.26558600
C	2.71949000	0.61060800	0.40014200
C	0.45058500	0.11451000	-0.24853900
C	1.42935200	1.20956400	-0.21641200
H	2.23335500	-1.84012600	1.44174000
H	3.00254600	-0.74650100	-1.27926600
H	2.54041000	0.51014600	1.48086200
H	3.50456600	-2.71676200	0.61418500
H	4.28522100	-0.76013300	-0.08968600
H	3.50695500	1.36923300	0.27877900
H	1.64708700	1.50349900	-1.26413600
C	1.61280100	-2.88734000	-0.36346700
H	1.70810900	-3.94973700	-0.09213500
H	1.85023200	-2.81488000	-1.43549700
C	0.12100000	-2.48656800	-0.16252000
H	-0.22517600	-2.80689900	0.83329800
O	1.04632400	2.34753700	0.54456500
H	-0.51408700	-2.99714400	-0.90110900
H	0.06935800	2.50430800	0.39130100
H	-1.64264300	0.79335700	-0.09477200
N	-1.74058200	2.69714900	0.23393300
C	-2.13377500	3.58670000	-0.86256600
H	-3.23033500	3.69708400	-0.92659700
H	-1.67749600	4.57031200	-0.69066000
H	-1.75717500	3.17970300	-1.80668800
C	-2.25705300	3.17103800	1.52161900
H	-1.80838000	4.14995300	1.73526100
H	-3.35677200	3.26559100	1.51037200
H	-1.96050100	2.46655800	2.30527100
O	-2.39863800	1.42178700	-0.02173900

C	2.64216100	-2.05650000	0.44205000

DIFO (*syn*-RC)
E = –3924.69
H = –3756.47
G = –3794.70
N_{imag} = 0

C	-1.77502800	-1.59030900	0.62506100
C	-1.23269200	-2.74916800	-0.22763400
C	0.41450700	0.61356700	0.08940000
C	0.25492900	-2.72240300	-0.60361100
C	1.14452000	-0.30458600	-0.53073100
C	0.77078600	-1.49479300	-1.36004000
H	-2.73039500	-1.92082400	1.05664700
H	-1.39651600	-3.69298700	0.31376300
H	0.47296100	-3.58584500	-1.24822700
H	-1.10573800	-1.41008900	1.48190000
H	-1.83249900	-2.81923200	-1.14749200
H	0.88130400	-2.82751500	0.29421500
F	1.93241300	-1.89010400	-2.02785500
C	-2.01353700	-0.27675100	-0.15768400
H	-1.98055000	-0.47877700	-1.23456900
H	-3.02793400	0.09364500	0.04882200
C	-1.05905900	0.88175000	0.17656900
H	-1.30581900	1.72895700	-0.48513300
H	-1.29683200	1.23156800	1.19322300
F	-0.12222200	-1.15558800	-2.38320800
H	2.22336800	-0.14869600	-0.36762400
N	1.34951500	1.64121900	0.85914900
C	1.05403000	1.54209200	2.33942300
H	0.01045000	1.77994400	2.57776700
H	1.73180900	2.24744700	2.82722100
H	1.30636200	0.52169500	2.63746200
C	1.02427100	3.02887300	0.35706800
H	1.69367600	3.70959800	0.88977300
H	-0.02526800	3.29350500	0.53333400
H	1.26085100	3.03587800	-0.70981500
O	2.66109300	1.40736700	0.67752500

N-COT (*syn*-RC)
E = –3796.09
H = –3624.62
G = –3661.74
N_{imag} = 0

N	0.57340000	-0.46990000	2.16490000
C	1.91440000	-0.07350000	2.59440000

O	-0.25170000	0.72870000	2.30940000
C	0.00730000	-1.46600000	3.07800000
H	2.56800000	-0.95450000	2.54650000
H	1.92030000	0.32620000	3.62540000
H	2.30180000	0.69290000	1.91400000
H	-0.69430000	0.80410000	1.43310000
H	-1.01460000	-1.70490000	2.76430000
H	-0.01290000	-1.10820000	4.12380000
H	0.61760000	-2.37720000	3.01820000
C	-1.39280000	-0.96120000	-0.84260000
H	-1.75960000	-1.61160000	-0.03700000
C	-1.69340000	0.46280000	-0.61260000
C	-1.42630000	1.64950000	-0.65280000
C	-0.63480000	2.83360000	-0.95270000
C	0.47180000	2.38430000	-1.94840000
C	1.27460000	1.12940000	-1.52630000
C	0.76140000	-0.29680000	-1.93700000
N	0.05770000	-1.15360000	-0.96600000
H	-1.88960000	-1.29630000	-1.76740000
H	-0.17980000	3.23530000	-0.03310000
H	-1.23390000	3.64660000	-1.38920000
H	1.16350000	3.22970000	-2.09000000
H	0.00080000	2.19970000	-2.92630000
H	1.44640000	1.14680000	-0.43830000
H	2.27190000	1.23000000	-1.98140000
H	1.65150000	-0.86910000	-2.24000000
H	0.13160000	-0.21710000	-2.83700000
H	0.46960000	-1.04260000	-0.02920000

O-COT (*syn*-RC)
E = –3670.50
H = –3508.27
G = –3543.09
N_{imag} = 0

N	-0.12500000	-0.71680000	3.02900000
C	1.17910000	-0.87710000	3.67010000
O	-0.34350000	0.72740000	2.92760000
C	-1.19770000	-1.21970000	3.88530000
H	1.36990000	-1.95080000	3.80210000
H	1.22740000	-0.37760000	4.65670000
H	1.95480000	-0.45890000	3.01950000
H	-0.44280000	0.85780000	1.95970000
H	-2.16180000	-1.04930000	3.39370000
H	-1.20870000	-0.73030000	4.87820000
H	-1.05480000	-2.30050000	4.02020000
C	-0.23850000	-0.85000000	-0.59590000

H	0.01360000	-1.43810000	0.29570000
C	-0.57560000	0.53670000	-0.27160000
C	-0.48290000	1.73550000	-0.44440000
C	-0.03370000	2.98720000	-1.02860000
C	0.54350000	2.62500000	-2.42970000
C	1.49380000	1.40460000	-2.46910000
C	0.87380000	-0.01940000	-2.59970000
O	0.93490000	-0.85740000	-1.42830000
H	-1.08450000	-1.34220000	-1.11200000
H	0.74680000	3.44280000	-0.39880000
H	-0.83810000	3.73020000	-1.12940000
H	1.07240000	3.51040000	-2.81400000
H	-0.30020000	2.44440000	-3.11330000
H	2.16240000	1.40490000	-1.59540000
H	2.14630000	1.54090000	-3.34480000
H	1.45710000	-0.58030000	-3.34260000
H	-0.16410000	0.04230000	-2.97010000

S-COT (*syn*-RC)
E = –3615.65
H = –3455.07
G = –3490.72
N_{imag} = 0

N	-0.10910000	-1.53290000	3.01110000
C	1.27250000	-1.70540000	3.45940000
O	-0.30920000	-0.08790000	2.88060000
C	-1.05400000	-1.96860000	4.03820000
H	1.45600000	-2.77810000	3.60900000
H	1.47720000	-1.16810000	4.40490000
H	1.95210000	-1.33700000	2.68340000
H	-0.51740000	0.01340000	1.92690000
H	-2.07640000	-1.79120000	3.68690000
H	-0.90620000	-1.43760000	4.99780000
H	-0.91610000	-3.04630000	4.20020000
C	-0.81150000	-1.85090000	-0.41610000
H	-0.52720000	-2.34160000	0.52620000
C	-0.92960000	-0.41730000	-0.27390000
C	-0.81760000	0.77630000	-0.46600000
C	-0.37390000	2.03810000	-1.03920000
C	0.16520000	1.75350000	-2.47090000
C	1.09270000	0.52050000	-2.58410000
C	0.39500000	-0.84710000	-2.84760000
S	0.58640000	-2.18960000	-1.60290000
H	-1.73790000	-2.30720000	-0.79200000
H	0.42700000	2.46570000	-0.41500000
H	-1.17630000	2.78960000	-1.07860000

H	0.70200000	2.65120000	-2.81240000
H	-0.69120000	1.62660000	-3.15040000
H	1.72170000	0.44100000	-1.68620000
H	1.78270000	0.70180000	-3.42120000
H	0.82340000	-1.31350000	-3.74510000
H	-0.67730000	-0.71920000	-3.04600000

Propyne (*syn*-TS)
E = –2199.70
H = –2102.13
G = –2128.92
N_{imag} = 1, ν = –550.07084

C	1.25556409	0.00000000	0.00000000
C	0.00000000	0.00000000	0.00000000
H	2.08858173	-0.68863387	-0.00018361
N	-0.28754016	2.14354815	0.00058595
C	-0.95053424	2.58412256	-1.23052427
H	-1.98471133	2.21567215	-1.24335833
H	-0.95807100	3.68366543	-1.30161051
C	-0.94912435	2.58330542	1.23276238
H	-1.98338849	2.21513484	1.24643742
H	-0.95627242	3.68279050	1.30475629
O	1.03251398	2.50085878	0.00000000
H	1.47604634	1.43889023	-0.00007668
H	-0.39841192	2.16989827	2.08315480
H	-0.40064292	2.17156948	-2.08185426
C	-1.35937028	-0.54746520	-0.00008565
H	-1.31562384	-1.64739495	-0.00040710
H	-1.92910593	-0.23766800	-0.88811314
H	-1.92899753	-0.23818144	0.88819709

HO-COT (*syn*-TS)
E = –4045.10
H = –3866.92
G = –3900.89
N_{imag} = 1, ν = –558.2332 i cm^{-1}

C	3.13119300	-0.57904300	0.15977400
C	-0.39279400	-0.47192800	-0.20497700
C	2.56482900	0.79926700	0.54137900
C	0.20309800	0.63567900	-0.23122500
C	1.47566400	1.38243900	-0.37687000
H	1.82301200	-1.73987700	1.45732300
H	3.39833000	-0.58004100	-0.91097600
H	2.18134200	0.78324900	1.57272300
H	3.04853400	-2.66468900	0.60993200
H	4.08747400	-0.66792500	0.69744600

H	3.38893500	1.52833400	0.51733800
H	1.81394000	1.26664700	-1.42520100
C	1.28321800	-2.33425900	-0.56905700
H	1.32230600	-3.43306600	-0.63351200
H	1.56703000	-1.96007900	-1.56554200
C	-0.17553700	-1.93845500	-0.28327400
H	-0.50347100	-2.39762100	0.66546900
O	1.41142400	2.79375200	-0.07413800
H	-0.83517800	-2.37095800	-1.05307800
H	0.82581500	3.19791500	-0.73565700
H	-1.70530400	0.01675300	0.02827400
N	-1.55027100	1.91795200	0.16619300
C	-1.87351400	2.82117000	-0.94394600
H	-2.88033100	3.25004700	-0.81474000
H	-1.14768400	3.64551100	-0.97899100
H	-1.84049000	2.25259500	-1.87821300
C	-1.42401800	2.57920700	1.47258500
H	-0.60199600	3.30222300	1.43337600
H	-2.36763000	3.07889200	1.74583200
H	-1.19427200	1.81072600	2.21598800
O	-2.44980200	0.89879800	0.21834000
C	2.31586000	-1.85486800	0.47789800

DIFO (*syn*-TS)
E = –3921.45
H = –3754.26
G = –3790.47
N_{imag} = 1, ν = –343.0553 i cm^{-1}

C	-2.54974600	-1.15838200	0.27869200
C	-2.99427900	0.21808700	0.82597300
C	0.41252800	-0.03924600	0.25431800
C	-2.49156400	1.48578100	0.10845600
C	0.02435000	1.15380000	0.35341600
C	-1.13472900	2.05002100	0.56805900
H	-3.36737700	-1.86510400	0.48148600
H	-4.09266300	0.23794400	0.76712300
H	-3.23576300	2.28467000	0.25415900
H	-2.46358900	-1.11725400	-0.81769900
H	-2.75750700	0.28204700	1.90159300
H	-2.45252000	1.29937900	-0.97629200
H	-0.96345700	3.01753400	0.07081600
C	-1.28340600	-1.80361000	0.86913600
H	-1.18795900	-1.56409300	1.93789100
H	-1.35683700	-2.89810100	0.78848300
C	0.04823100	-1.45735800	0.20818000
F	1.02752700	-2.25877300	0.81640000

F	0.01850200	-1.90213000	-1.12442700
H	-1.18095000	2.28507400	1.64621200
H	1.37708700	1.73319500	0.10144300
N	2.47027400	0.20539800	-0.11904900
C	2.82793200	-0.33101100	-1.44016600
H	2.71503500	-1.42074400	-1.42635500
H	3.86544800	-0.06278400	-1.69287100
H	2.14738600	0.09814700	-2.18018100
C	3.30969500	-0.29692300	0.97990900
H	4.34714300	0.04457200	0.84433900
H	3.26980500	-1.39015900	0.99541400
H	2.91632700	0.09931200	1.92054000
O	2.47085400	1.57221900	-0.12571700

N-COT (*syn*-TS)
E = –3788.45
H = –3619.78
G = –3653.80
N_{imag} = 1, ν = –452.9115 i cm^{-1}

N	-0.36282889	2.12716802	-0.00205705
C	-1.03803693	2.56098774	1.22381524
O	0.95014925	2.51467470	-0.00000000
C	-1.03153126	2.54125365	-1.23901329
H	-2.06452458	2.17149568	1.23506776
H	-1.06818697	3.66079642	1.28943501
H	-0.48366171	2.16381633	2.07948083
H	1.41355497	1.46569129	0.00856013
H	-0.46746715	2.13631675	-2.08449365
H	-1.06867776	3.64007727	-1.31928450
H	-2.05398014	2.14134223	-1.25242886
C	-1.30857111	-0.68508699	0.01054298
H	-2.01228718	-0.19145838	0.69841849
C	-0.00000000	-0.00000000	-0.00000000
C	1.25775073	0.00000000	-0.00000000
C	2.41791651	-0.92572031	-0.05563571
C	2.12133672	-2.25450491	-0.77619153
C	1.08449847	-3.18838847	-0.12067598
C	-0.42476433	-2.99818403	-0.42784869
N	-1.23095268	-2.09488245	0.40553718
H	-1.77519729	-0.63721714	-0.98810930
H	2.75518168	-1.13356868	0.97528894
H	3.27525596	-0.43928729	-0.54692047
H	3.07306054	-2.80436178	-0.85629478
H	1.80884978	-2.03451718	-1.80995959
H	1.22579074	-3.19472168	0.97432989
H	1.32516918	-4.21232384	-0.44883849

H	-0.89156550	-3.99273967	-0.34032557
H	-0.55637456	-2.69094787	-1.47730368
H	-0.92990555	-2.15958300	1.37950827

O-COT (*syn*-TS)
E = –3664.95
H = –3504.05
G = –3537.80
N_{imag} = 1, ν = –387.2463 i cm^{-1}

N	-0.40128574	2.10846369	-0.00019814
C	-1.07778205	2.52297806	1.23273523
O	0.90561651	2.51505164	0.00000000
C	-1.07831702	2.52311746	-1.23306065
H	-2.10078365	2.12475977	1.24018165
H	-1.11561653	3.62135269	1.30982830
H	-0.51949219	2.11897379	2.08258244
H	1.38755674	1.48760589	0.00713332
H	-0.51412803	2.12719345	-2.08269568
H	-1.12450903	3.62164573	-1.30650343
H	-2.09728046	2.11459331	-1.24407967
C	-1.29450372	-0.69559507	0.01665496
H	-1.96381130	-0.29966519	0.79444088
C	0.00000000	0.00000000	0.00000000
C	1.25506389	0.00000000	0.00000000
C	2.42161664	-0.91791643	-0.03104213
C	2.16168045	-2.21949056	-0.81416372
C	1.06292942	-3.15403952	-0.27411974
C	-0.40915633	-2.86148708	-0.62657041
O	-1.13810206	-2.08117014	0.33730673
H	-1.81504408	-0.58627126	-0.95644027
H	2.69813056	-1.16345996	1.00902908
H	3.30356386	-0.41655083	-0.45871881
H	3.10785743	-2.78377448	-0.83839773
H	1.93377581	-1.96250775	-1.86151715
H	1.13757582	-3.24894597	0.82079139
H	1.27127541	-4.15860402	-0.67665035
H	-0.95000759	-3.81876007	-0.66262294
H	-0.48603161	-2.39757120	-1.62620550

S-COT (*syn*-TS)
E = –3606.70
H = –3447.82
G = –3482.88
N_{imag} = 1, ν = –593.6325 i cm^{-1}

N	-0.33079414	2.14493017	-0.00664950
C	-1.00597342	2.57049940	1.22296871

O	0.98363800	2.50549420	0.00000000
C	-0.99425856	2.57187918	-1.24240221
H	-2.03519000	2.18814278	1.22889659
H	-1.02924752	3.66965047	1.29828989
H	-0.45522732	2.16128431	2.07523243
H	1.42986304	1.42321406	0.00567862
H	-0.43229874	2.16740036	-2.08955342
H	-1.02006647	3.67125365	-1.31563631
H	-2.02115783	2.18364944	-1.26025849
C	-1.32052812	-0.61439549	-0.01719122
H	-1.98023792	-0.22562088	0.77274864
C	0.00000000	0.00000000	0.00000000
C	1.25786874	-0.00000000	0.00000000
C	2.44768709	-0.89323233	-0.02143577
C	2.34205881	-2.09173362	-0.98663594
C	1.36840181	-3.22631009	-0.62052482
C	-0.12591492	-3.05430114	-0.96191875
S	-1.24365042	-2.42154302	0.34989689
H	-1.83653554	-0.46681716	-0.97866030
H	2.62224410	-1.26499904	1.00288298
H	3.34877400	-0.31927983	-0.28579521
H	3.34874493	-2.53380815	-1.05629174
H	2.10559656	-1.71798523	-1.99607134
H	1.46656164	-3.47838500	0.44661405
H	1.70508298	-4.11916019	-1.17244233
H	-0.55429368	-4.04108727	-1.18473554
H	-0.26869019	-2.43883272	-1.86177734

Propyne (*syn*-P)
E = –2222.41
H = –2121.03
G = –2147.22
N_{imag} = 0

C	1.32710387	0.00000000	0.00000000
C	0.00000000	0.00000000	0.00000000
H	1.88118102	-0.93641386	-0.00020857
N	-0.65412775	1.39850780	0.00051526
C	-1.52237666	1.51877769	-1.23479058
H	-2.31605373	0.76121361	-1.25867306
H	-1.94417240	2.52676651	-1.20674030
C	-1.52047508	1.51849203	1.23721933
H	-2.31397195	0.76077699	1.26234869
H	-1.94251570	2.52639779	1.20995065
O	0.24459126	2.40391673	0.00000000
H	1.83781836	0.96258278	0.00015198
H	-0.84942807	1.42006486	2.09400433

H	-0.85272704	1.42028836	-2.09267385
C	-0.89306818	-1.20441846	-0.00023786
H	-1.54289984	-1.24847022	-0.88597667
H	-1.54354512	-1.24837972	0.88504223
H	-0.27367462	-2.10926764	0.00007176

HO-COT (*syn*-P)

E = –4062.89

H = –3880.40

G = –3914.41

N_{imag} = 0

C	3.02141000	-0.38183000	0.55216500
C	-0.66589400	-0.56690700	-0.24885300
C	2.17035100	0.88654700	0.69604800
C	-0.22054000	0.68472600	-0.20493100
C	1.19322000	1.16138700	-0.48254600
H	1.61613900	-1.81007300	1.38795100
H	3.64097300	-0.29594600	-0.35752200
H	1.61301700	0.86033700	1.64280200
H	3.04150200	-2.51543000	0.64336500
H	3.72834900	-0.39994700	1.39598900
H	2.85424800	1.74827700	0.76606200
H	1.54721000	0.59501300	-1.35862300
C	1.48136100	-2.02699100	-0.77647500
H	1.66271600	-3.07092100	-1.07270300
H	1.87102000	-1.42322600	-1.60976800
C	-0.04158300	-1.87108600	-0.65182900
H	-0.39875000	-2.62321700	0.07290800
O	1.20087000	2.56409800	-0.84590800
H	-0.50949600	-2.17525700	-1.60545400
H	2.12184300	2.79376300	-1.05452100
H	-1.71250400	-0.61197300	0.09307600
N	-1.34288300	1.69056800	0.29844200
C	-1.72441200	2.58701400	-0.86063600
H	-2.50427200	3.25272500	-0.47889800
H	-0.86031100	3.13853400	-1.23997200
H	-2.14554800	1.92742600	-1.62297300
C	-0.79208700	2.51345800	1.43603200
H	0.10870400	3.05689300	1.13198400
H	-1.59282100	3.19835900	1.73004900
H	-0.59535800	1.81559300	2.25357000
O	-2.45517300	1.05920800	0.72251900
C	2.28282300	-1.72985900	0.51406600

DIFO (*syn*-P)

E = –3937.53

H = –3767.11
G = –3801.63
N_{imag} = 0

C	-2.10189100	-1.13811900	-0.05089900
C	-3.01792400	0.08483700	0.11833400
C	0.75034500	0.07147300	0.48218100
C	-2.34648900	1.46534000	0.10085900
C	0.14784600	1.25327200	0.58404000
C	-1.19322800	1.66665800	1.11976200
H	-2.73551800	-2.01117500	-0.25707300
H	-3.77466000	0.06865700	-0.68090600
H	-3.12135600	2.21947800	0.30264700
H	-1.46691700	-1.01464500	-0.94070400
H	-3.57662600	-0.02976900	1.06256300
H	-1.96562000	1.68171000	-0.90905600
H	-1.12823900	2.73675000	1.35790500
C	-1.22880700	-1.45325100	1.18460600
H	-1.54973000	-0.86593000	2.05350500
H	-1.32836900	-2.50772700	1.47597600
C	0.26991400	-1.25079500	1.01578100
F	0.86401600	-1.48992400	2.26581800
F	0.73592000	-2.30618900	0.21015200
H	-1.43865500	1.16005100	2.06399200
H	0.80014500	2.03664200	0.16161400
N	2.17648900	0.18666400	-0.20891600
C	2.11767100	-0.48038500	-1.56788100
H	1.86593900	-1.54106300	-1.49212800
H	3.10588600	-0.33030200	-2.01139600
H	1.37034000	0.06869800	-2.14546500
C	3.20314500	-0.49798800	0.66144900
H	4.16136800	-0.36460200	0.15191400
H	2.96809300	-1.55773500	0.80088200
H	3.21111300	0.03380000	1.61499800
O	2.54160100	1.46889600	-0.39348200

N-COT (*syn*-P)
E = –3807.38
H = –3635.26
G = –3667.69
N_{imag} = 0

N	0.37320000	-0.03850000	2.22450000
C	1.54330000	-0.99630000	2.20380000
O	0.68290000	1.00030000	3.02660000
C	-0.84960000	-0.72880000	2.78990000
H	1.35930000	-1.87130000	1.56810000
H	1.71160000	-1.29900000	3.24110000

H	2.39980000	-0.41760000	1.84890000
H	0.34460000	2.22490000	1.49110000
H	-1.63830000	0.02660000	2.81240000
H	-0.58940000	-1.02600000	3.80980000
H	-1.15260000	-1.59970000	2.19550000
C	-0.19620000	-0.75160000	-0.22780000
H	-0.38370000	-1.68200000	0.32860000
C	0.04700000	0.37860000	0.74720000
C	0.07990000	1.69630000	0.56390000
C	-0.18010000	2.54600000	-0.64170000
C	-0.41550000	1.91790000	-2.02170000
C	0.78940000	1.15730000	-2.61930000
C	0.75070000	-0.38010000	-2.51230000
N	0.87720000	-1.01760000	-1.20320000
H	-1.12030000	-0.56540000	-0.79140000
H	0.66440000	3.25410000	-0.72340000
H	-1.04310000	3.18990000	-0.39480000
H	-0.67820000	2.74530000	-2.69720000
H	-1.30360000	1.26730000	-2.00480000
H	1.72500000	1.53310000	-2.17250000
H	0.85690000	1.38710000	-3.69450000
H	1.56050000	-0.78840000	-3.13640000
H	-0.19050000	-0.74050000	-2.96070000
H	1.78430000	-0.79700000	-0.79020000

O-COT (*syn*-P)
E = –3685.83
H = –3521.53
G = –3553.64
N_{imag} = 0

N	-0.17080000	-0.06030000	2.17990000
C	0.82560000	-0.81610000	3.02990000
O	-0.63660000	0.99210000	2.88170000
C	-1.33950000	-0.96200000	1.84450000
H	1.20430000	-1.71120000	2.51840000
H	0.30240000	-1.08470000	3.95180000
H	1.63420000	-0.11550000	3.25180000
H	-0.07640000	2.20130000	1.33310000
H	-2.03030000	-0.35800000	1.25170000
H	-1.80310000	-1.22430000	2.79930000
H	-1.03000000	-1.86350000	1.30030000
C	1.21710000	-0.76090000	0.07360000
H	2.22060000	-0.96920000	0.47720000
C	0.54580000	0.34330000	0.85760000
C	0.48910000	1.64230000	0.57660000
C	1.05370000	2.38680000	-0.60470000

C	0.13350000	2.35200000	-1.85510000
C	0.31380000	1.12960000	-2.76690000
C	0.24650000	-0.22760000	-2.07550000
O	1.43840000	-0.42990000	-1.28930000
H	0.64370000	-1.70530000	0.15460000
H	2.04710000	2.00880000	-0.87940000
H	1.17820000	3.43550000	-0.30110000
H	0.32520000	3.25340000	-2.45590000
H	-0.91540000	2.42070000	-1.52680000
H	1.28640000	1.18910000	-3.28060000
H	-0.45550000	1.15260000	-3.55520000
H	0.19870000	-1.03590000	-2.82380000
H	-0.65030000	-0.29930000	-1.43990000

S-COT (*syn*-P)
E = –3628.77
H = –3466.22
G = –3499.38
$\boldsymbol{N}_{\text{imag}}$ = 0

N	-0.32840000	-0.99390000	2.09330000
C	0.70600000	-1.61420000	3.01040000
O	-0.84860000	0.09120000	2.70560000
C	-1.43370000	-1.99320000	1.83660000
H	1.14870000	-2.52320000	2.58310000
H	0.18530000	-1.83850000	3.94520000
H	1.46400000	-0.84630000	3.18020000
H	-0.08720000	1.28980000	1.18580000
H	-2.17530000	-1.48030000	1.22060000
H	-1.86250000	-2.22790000	2.81490000
H	-1.06650000	-2.89190000	1.32790000
C	0.93340000	-1.73960000	-0.09220000
H	1.84930000	-1.38220000	-0.58170000
C	0.37770000	-0.65230000	0.76120000
C	0.41990000	0.64620000	0.45860000
C	1.08680000	1.24650000	-0.74540000
C	0.16040000	1.44610000	-1.96730000
C	-0.17400000	0.17860000	-2.76970000
C	-1.03710000	-0.87170000	-2.04190000
S	-0.21310000	-2.37640000	-1.41200000
H	1.19880000	-2.64530000	0.46670000
H	1.95340000	0.64200000	-1.06060000
H	1.48430000	2.23020000	-0.45540000
H	0.64890000	2.15770000	-2.65030000
H	-0.77380000	1.92780000	-1.63810000
H	0.75220000	-0.29470000	-3.13040000
H	-0.72020000	0.49680000	-3.67140000

H	-1.76710000	-1.31410000	-2.73460000
H	-1.61250000	-0.41010000	-1.22760000

Table S9. Electronic energies (kcal mol^{-1}), enthalpies (kcal mol^{-1}), Gibbs free energies (kcal mol^{-1}), the number of imaginary vibrational frequencies (and their wavelength), and cartesian coordinates (Å), for all stationary points of the newly proposed biorthogonal reagents (reactant complex RC, transition state TS, product P), computed at ZORA-BP86/TZ2P using AMS2021.

DF-BCN (*reactant*)
E = –2801.01
H = –2691.42
G = –2719.89
N_{imag} = 0

C	0.05779948	-2.81398247	-0.92053611
C	1.58226806	-2.72496541	-0.88387334
C	0.00000000	0.00000000	0.00000000
C	2.42770448	-2.05998604	0.18886274
C	1.21305870	-0.00000000	0.00000000
C	2.57112774	-0.51643849	0.00023595
H	2.00266451	-2.24454084	1.18565520
H	3.43319529	-2.51035815	0.18493219
H	3.18237486	-0.08472494	0.80614273
H	3.08012633	-0.28640258	-0.94862401
C	-0.90487034	-2.27121114	0.11965620
H	-0.51097017	-2.39430562	1.13715305
H	-1.85941589	-2.81774959	0.06743331
C	-1.23707612	-0.77338111	-0.04439264
F	-2.12673831	-0.40088282	0.95455065
F	-1.91353429	-0.57005801	-1.24278514
C	0.88907307	-4.04643823	-0.63758493
H	0.95071259	-4.83355713	-1.38732341
H	-0.34691481	-2.75723013	-1.93230359
H	2.01591343	-2.62022662	-1.88183685
H	0.88648967	-4.40918386	0.39122836

DCl-BCN (*reactant*)
E = –2705.99
H = –2597.77
G = –2627.88
N_{imag} = 0

C	0.06103172	-2.81747609	-0.92857634
C	1.58532531	-2.72569246	-0.88202710
C	0.00114397	-0.04182981	-0.00879780
C	2.42850985	-2.05571946	0.18995261
C	1.21330797	0.00461102	-0.00055983

C	2.57196555	-0.51276032	0.00006376
H	2.00242191	-2.23845782	1.18670726
H	3.43388217	-2.50655363	0.18757998
H	3.18492638	-0.08073551	0.80453454
H	3.08056545	-0.28270187	-0.94925668
C	-0.91531734	-2.28056144	0.10238352
H	-0.52324209	-2.40591402	1.12096739
H	-1.86309083	-2.83557997	0.04462690
C	-1.23888500	-0.76815407	-0.04542664
Cl	-2.35324271	-0.27714181	1.32145271
Cl	-2.12809839	-0.46853298	-1.62015446
C	0.89295768	-4.04619346	-0.62877252
H	0.95753245	-4.84002818	-1.37115978
H	-0.33696912	-2.77067415	-1.94335726
H	2.02329829	-2.62801830	-1.87885537
H	0.88631838	-4.39980288	0.40322054

DCF_3-BCN (*reactant*)
E = –3589.94
H = –3461.71
G = –3498.86
N_{imag} = 0

C	0.11931461	-2.83017196	-0.97841354
C	1.63818901	-2.70437579	-0.86806900
C	-0.04797472	-0.05746897	-0.03921183
C	2.42571552	-2.00750542	0.22861890
C	1.15997401	0.01535388	-0.00276309
C	2.53265983	-0.46310915	0.03567684
H	1.97105060	-2.19697695	1.21134999
H	3.44151191	-2.43311961	0.26184390
H	3.11087059	-0.01225023	0.85514618
H	3.06045646	-0.22248210	-0.90016770
C	-0.91792748	-2.32596505	0.00949160
H	-0.55558543	-2.46791921	1.03421716
H	-1.83576046	-2.92254487	-0.09479856
C	-1.30600371	-0.79462469	-0.09322641
C	-2.19782475	-0.43314624	1.13984571
C	-2.07258838	-0.49286698	-1.42132867
C	0.96580168	-4.03647289	-0.62666244
H	1.07697908	-4.83857568	-1.35443721
H	-0.22519922	-2.81625325	-2.01187578
H	2.11314968	-2.60685187	-1.84777257
H	0.92415205	-4.37631052	0.40920410
F	-2.55165240	0.77075443	-1.47041329
F	-3.12286728	-1.33614983	-1.59768401
F	-1.55031158	-0.74565306	2.29413346

F	-3.36611047	-1.12507992	1.13799208
F	-2.49809874	0.88186240	1.19930698
F	-1.25275074	-0.64908397	-2.49165564

DF-*p*-MOBO (*reactant*)
E = –3425.27
H = –3303.49
G = –3334.55
N_{imag} = 0

C	2.55072064	-0.56746836	0.03611868
H	3.17497571	-0.21808869	-0.79914852
C	1.21364362	0.00108793	0.00379029
C	0.00089670	0.00680713	-0.00914648
C	-1.22273854	-0.79007874	0.01623578
C	-0.83811609	-2.28859274	-0.02720449
C	0.07388388	-2.74542853	1.10086262
C	1.49268727	-2.67984539	1.07808978
C	2.32682965	-2.11346047	-0.06160542
H	3.07300415	-0.30926568	0.96941425
F	-2.04607019	-0.51552358	-1.06772471
F	-1.98482462	-0.52418435	1.14238863
H	-1.64332918	-3.33401603	2.25125081
H	-0.35904188	-4.14914137	4.20052069
H	3.31006929	-2.60551903	-0.06133908
H	1.86171121	-2.32220343	-1.03496490
C	2.19878140	-3.15853756	2.19322714
C	1.55764088	-3.68089052	3.31482607
C	0.16594512	-3.74115611	3.33687173
C	-0.55407304	-3.28146559	2.23624866
H	3.28989603	-3.12172723	2.17306854
H	2.14323987	-4.04387893	4.15955113
H	-1.77953794	-2.85484226	0.00274171
H	-0.38264202	-2.45388735	-1.01213007

DCl-*p*-MOBO (*reactant*)
E = –3329.79
H = –3209.39
G = –3242.07
N_{imag} = 0

C	2.55308526	-0.56050270	0.03740499
H	3.18466623	-0.21740649	-0.79502404
C	1.21780495	0.01365333	-0.00489940
C	0.00596727	-0.02965698	-0.02179104
C	-1.22442169	-0.77370770	0.00104363
C	-0.85652692	-2.28885365	-0.00478983
C	0.06802257	-2.74023216	1.11762358

C	1.48748918	-2.66838089	1.08661679
C	2.32074762	-2.10479406	-0.05578973
H	3.07181116	-0.30101892	0.97244520
Cl	-2.24161237	-0.43163320	-1.48517903
Cl	-2.22782623	-0.35182814	1.46659551
H	-1.63799380	-3.36450016	2.26529671
H	-0.33972495	-4.19415153	4.19844586
H	3.30060880	-2.60375580	-0.05739488
H	1.85150673	-2.31175219	-1.02753018
C	2.20081671	-3.15370324	2.19450399
C	1.56888961	-3.69248891	3.31341350
C	0.17816371	-3.76779726	3.33943623
C	-0.54976792	-3.29945461	2.24790315
H	3.29159605	-3.11171191	2.16891583
H	2.16143032	-4.05997376	4.15137924
H	-1.79519336	-2.85643495	0.03654294
H	-0.40359356	-2.47202898	-0.98845177

DCF_3-*p*-MOBO (*reactant*)
E = –4213.28
H = –4072.91
G = –4112.45
$\boldsymbol{N}_{imag}$ = 0

C	2.50959703	-0.47823504	0.10918472
H	3.13344101	-0.09273682	-0.71027435
C	1.15794646	0.05850194	0.07914154
C	-0.05045457	-0.00653311	0.05502453
C	-1.29499455	-0.76929524	0.04191894
C	-0.85917705	-2.29317217	0.03141677
C	0.08731341	-2.75668123	1.13272530
C	1.50421263	-2.64788407	1.09383599
C	2.31858527	-2.02244864	-0.03125370
H	3.01810596	-0.23318996	1.05353213
C	-2.09052409	-0.48240462	-1.27500911
C	-2.17428835	-0.42642694	1.28891550
H	-1.58795361	-3.50605520	2.25356160
H	-0.25283923	-4.40011507	4.12609795
H	3.31037545	-2.49685673	-0.05112970
H	1.85175180	-2.21162611	-1.00797184
C	2.23913011	-3.16857022	2.17145931
C	1.63335717	-3.78534484	3.26388313
C	0.24627133	-3.90846490	3.29127332
C	-0.50248922	-3.40219541	2.23181207
H	3.32796077	-3.09413099	2.14022212
H	2.24314438	-4.17801887	4.07762722
H	-1.76942167	-2.90571576	0.06269935

H	-0.39549919	-2.44857181	-0.94941782
F	-2.38056393	0.82609101	-1.43258675
F	-3.25699359	-1.17562457	-1.31803208
F	-1.43488621	-0.46347609	2.41973698
F	-3.19387659	-1.31365376	1.44158089
F	-2.72201612	0.80868892	1.20826642
F	-1.36053127	-0.86028745	-2.35840321

DF-O-MOBO (*reactant*)
E = –3199.38
H = –3092.42
G = –3123.39
N_{imag} = 0

C	0.02421741	-2.66016631	0.07914767
C	1.18869594	-2.63877755	-0.95908630
C	0.02504882	0.06537902	-0.08811920
C	2.49207045	-1.96926347	-0.56522702
C	1.23924517	-0.00551882	-0.10235752
C	2.52511106	-0.59315475	-0.19270526
H	0.34253419	-2.29889364	1.06496778
H	1.41385315	-3.67397806	-1.24862610
O	-1.16268581	-1.93783958	-0.35489072
H	-0.34343593	-3.68504104	0.19569009
H	0.79265186	-2.14750235	-1.86161908
C	-1.26803528	-0.60637513	0.00649024
C	3.73909835	0.04398495	0.10441557
C	4.93191779	-0.67235253	0.04335693
C	4.91350851	-2.02487941	-0.30416840
C	3.70508922	-2.66134477	-0.60219733
H	3.73365973	1.09595647	0.38624439
H	5.87519423	-0.17782499	0.27312290
H	5.84498216	-2.58937171	-0.34599465
H	3.70465790	-3.71717844	-0.87789160
F	-1.76012642	-0.50346326	1.30681116
F	-2.23181385	-0.06989248	-0.79798338

DIBBO (*reactant*)
E = –3846.65
H = –3717.98
G = –3750.56
N_{imag} = 0

C	0.08854613	-2.68564814	0.59062693
C	1.13507498	-2.68564814	-0.59062693
C	0.00000000	0.00000000	0.00000000
C	2.47919498	-2.03462109	-0.30834539
C	1.22362111	0.00000000	0.00000000

C	2.48994239	-0.59276137	-0.06678415
H	0.54034873	-2.17804493	1.45478977
H	-2.36387797	-3.85549751	0.50106850
C	-1.25557386	-2.03462109	0.30834539
H	-0.09940755	-3.72324811	0.89606508
H	-4.58149935	-2.90992796	0.15200227
C	-1.26632128	-0.59276137	0.06678415
B	3.88747983	-0.27014379	0.09422762
C	5.19246092	-0.85961467	0.11749379
C	4.94417248	-2.23700433	-0.11828186
C	3.66374148	-2.78402273	-0.31609846
H	1.32302866	-3.72324811	-0.89606508
H	6.19941159	-0.48697692	0.26513107
H	5.80512046	-2.90992796	-0.15200227
H	3.58749909	-3.85549751	-0.50106850
B	-2.66385872	-0.27014379	-0.09422762
C	-3.96883980	-0.85961467	-0.11749379
C	-3.72055137	-2.23700433	0.11828186
C	-2.44012037	-2.78402273	0.31609846
H	0.68327238	-2.17804493	-1.45478977
H	-4.97579047	-0.48697692	-0.26513107

DITO (*reactant*)
E = –3224.36
H = –3119.60
G = –3150.55
N_{imag} = 0

C	0.07682202	-2.81591158	0.58556178
C	1.15101674	-2.81591158	-0.58556178
C	0.00000000	0.00000000	0.00000000
C	2.43213989	-2.05898919	-0.33491851
C	1.22783876	0.00000000	0.00000000
C	2.44624143	-0.67119751	-0.12078902
H	0.55683744	-2.40030512	1.48272494
S	4.06425373	-0.06820920	0.08955218
C	-1.20430114	-2.05898919	0.33491851
H	-0.17970651	-3.85670463	0.82195900
C	4.72237461	-1.65023846	-0.10101322
C	-1.21840267	-0.67119751	0.12078902
C	3.74524959	-2.59762733	-0.30732620
H	1.40754527	-3.85670463	-0.82195900
H	0.67100131	-2.40030512	-1.48272494
S	-2.83641498	-0.06820920	-0.08955218
C	-3.49453585	-1.65023846	0.10101322
C	-2.51741083	-2.59762733	0.30732620
H	-4.56993630	-1.79117557	0.06555976

H	-2.73887008	-3.65363034	0.45426188
H	3.96670884	-3.65363034	-0.45426188
H	5.79777505	-1.79117557	-0.06555976

F-COD (*reactant*)
E = –2247.79
H = –2170.87
G = –2197.54
N_{imag} = 0

C	0.57226549	0.55961320	0.97287107
C	-0.57226549	-0.55961320	0.97287107
C	-0.01460343	0.61280294	-1.76256842
C	-0.28479425	-1.80299046	0.19088391
C	0.01460343	-0.61280294	-1.76256842
C	-0.05365134	-1.87395916	-1.14463745
H	1.48990631	0.10256538	0.58034205
H	-1.48990631	-0.10256538	0.58034205
C	0.28479425	1.80299046	0.19088391
H	0.76872995	0.85723985	2.00940140
H	-0.76872995	-0.85723985	2.00940140
C	0.05365134	1.87395916	-1.14463745
F	0.21894140	2.94192741	0.93751647
F	-0.21894140	-2.94192741	0.93751647
H	0.13575719	-2.82367554	-1.64239483
H	-0.13575719	2.82367554	-1.64239483

CF_3-COD (*reactant*)
E = –3048.91
H = –2953.22
G = –2989.39
N_{imag} = 0

C	0.51317841	0.56717581	0.95428450
C	-0.61493721	-0.56470990	0.85674903
C	0.18013645	0.60753851	-1.78408508
C	-0.21852288	-1.84032455	0.15916822
C	0.19370486	-0.61705917	-1.78028969
C	0.12107565	-1.87612385	-1.16033782
H	1.45044377	0.13860467	0.57723163
H	-1.47100311	-0.13820342	0.31865129
C	0.24636722	1.83959738	0.19219574
H	0.67073244	0.80063668	2.01256882
H	-0.95638736	-0.79357493	1.87180349
C	0.14365285	1.86936737	-1.16657649
C	0.08412034	3.10196261	0.99439747
C	-0.19955487	-3.09893838	0.98310469
H	0.36015383	-2.80760213	-1.67014374

H	-0.00143940	2.79852994	-1.71459887
F	0.67378820	-2.98261018	2.03137883
F	0.16395568	-4.19961528	0.27776234
F	-1.42449656	-3.36025609	1.53136697
F	1.19337615	3.36487062	1.74922777
F	-0.96083544	2.99099414	1.87240467
F	-0.14750901	4.19974015	0.23121296

DF-BCN (TS)

E = –4107.50

H = –3936.08

G = –3972.66

$\boldsymbol{N}_{\text{imag}}$ = 1, ν = –103.6958 i cm^{-1}

C	2.31461533	2.65588954	-1.23290548
H	3.33606597	2.25410929	-1.22402120
H	2.35703604	3.75303903	-1.32575818
H	1.76340495	2.24326996	-2.08355288
C	2.28461261	2.70685615	1.22905784
H	2.32344788	3.80693650	1.27815513
H	3.30704986	2.30865347	1.26079014
H	1.71421414	2.32795753	2.08276149
O	0.31606559	2.69093820	-0.02553424
H	-0.18232076	1.74066982	-0.01432970
N	1.62826506	2.25730662	-0.00120536
C	0.06322019	-2.81501451	-0.95034232
C	1.57964163	-2.72254471	-0.90964567
C	-0.04196803	0.13563305	0.01711125
C	2.35767648	-2.07207049	0.21497843
C	1.20882871	0.11605688	0.03093948
C	2.52097299	-0.53929742	0.04797516
H	1.87678658	-2.27267121	1.18296683
H	3.36263031	-2.52100938	0.26891056
H	3.14466125	-0.14726905	0.86660955
H	3.06532024	-0.32547681	-0.88698762
C	-0.84415011	-2.28347459	0.13814802
H	-0.39986297	-2.41844696	1.13328338
H	-1.79843153	-2.83217196	0.13268698
C	-1.18311649	-0.79597314	-0.00572204
F	-2.07956146	-0.46947779	1.01514953
F	-1.89652208	-0.61721364	-1.19582563
C	0.89501259	-4.05657227	-0.71393045
H	0.95986817	-4.80716062	-1.49985894
H	-0.36560510	-2.72366093	-1.94907222
H	2.03926411	-2.57810945	-1.89072467
H	0.89364603	-4.46574626	0.29715199

DCl-BCN (TS)
E = –4011.62
H = –3841.59
G = –3879.75
N_{imag} = 1, ν = –114.7806 i cm^{-1}

C	0.11088980	-2.67028955	-0.95994093
C	1.62440226	-2.54887768	-0.90598956
C	-0.07061935	0.25281462	0.01929204
C	2.37995367	-1.88506874	0.22603584
C	1.18178606	0.27608269	0.03875374
C	2.50868473	-0.34930141	0.06422744
H	1.89728575	-2.09994212	1.19012215
H	3.39436626	-2.31141957	0.28494605
H	3.11761676	0.05486673	0.88824328
H	3.05535703	-0.12120552	-0.86624083
C	-0.81848600	-2.17054846	0.12538726
H	-0.36656732	-2.29946907	1.11893324
H	-1.74934110	-2.75539277	0.11835015
C	-1.18850013	-0.67974906	0.00992644
Cl	-2.31274033	-0.29358288	1.42033301
Cl	-2.16032499	-0.43020565	-1.53802204
C	0.96536633	-3.89580758	-0.71511171
H	1.04762646	-4.64514860	-1.50057414
H	-0.31332063	-2.58938061	-1.96127860
H	2.08909779	-2.39435723	-1.88315404
H	0.96551425	-4.30489157	0.29600855
C	2.22912547	2.81239071	-1.24715763
H	3.25847464	2.43181067	-1.23169199
H	2.24897417	3.90913788	-1.34921263
H	1.68852036	2.38079056	-2.09512259
C	2.19091099	2.88442260	1.21549844
H	2.20661325	3.98524996	1.25461019
H	3.22108563	2.50757657	1.25312386
H	1.62563404	2.50087194	2.07045209
O	0.22712042	2.81641657	-0.04519858
H	-0.24864090	1.85516367	-0.02641857
N	1.54733046	2.41136649	-0.01322855

DCF_3-BCN (TS)
E = –4894.53
H = –4704.76
G = –4749.59
N_{imag} = 1, ν = –168.7268 i cm^{-1}

C	0.18467935	-2.65415024	-1.04340624
C	1.69034508	-2.52115990	-0.89753384
C	-0.10212179	0.27303246	-0.03263744

C	2.36212616	-1.86168950	0.28771317
C	1.15029130	0.29694318	0.04521576
C	2.47714927	-0.32475373	0.14900693
H	1.82359555	-2.09541686	1.21707530
H	3.37683740	-2.27459034	0.40600010
H	3.02876517	0.07747928	1.01338469
H	3.08013907	-0.08305321	-0.74223727
C	-0.80404134	-2.19019170	0.00465075
H	-0.37776776	-2.34241702	1.00339353
H	-1.70569936	-2.81640500	-0.04970869
C	-1.24067599	-0.68528292	-0.05771835
C	-2.12565517	-0.41504172	1.20607697
C	-2.08307946	-0.42057554	-1.34938370
C	1.03480221	-3.87558261	-0.76042818
H	1.16970339	-4.61481397	-1.54806515
H	-0.17369157	-2.57757770	-2.06887879
H	2.21359128	-2.34990475	-1.84163213
H	0.97881069	-4.29644844	0.24431588
C	2.25738810	2.75098008	-1.26126684
H	3.27997360	2.35952690	-1.18749489
H	2.29517974	3.84397836	-1.38953676
H	1.75002651	2.30324058	-2.12109727
C	2.10766872	2.88905152	1.19594163
H	2.13603082	3.98976789	1.20531619
H	3.12959524	2.50026917	1.29025529
H	1.49779431	2.53580677	2.03287649
O	0.20344768	2.80732904	-0.15232466
H	-0.27882178	1.82542070	-0.12505546
N	1.51118672	2.39428051	-0.04962924
F	-1.28567756	-0.45814384	-2.45153839
F	-2.69306920	0.78666347	-1.34862366
F	-3.04990200	-1.35994201	-1.53272269
F	-1.41497212	-0.68618751	2.33746341
F	-3.22985360	-1.20904218	1.23541498
F	-2.54607473	0.86385412	1.30652169

DF-*p*-MOBO (TS)
E = –4732.95
H = –4548.58
G = –4589.03
N_{imag} = 1, ν = –7.4465 i cm^{-1}

C	2.35885701	2.96107781	-1.22512859
H	3.43120719	2.72837453	-1.18335452
H	2.23262156	4.05116033	-1.35175080
H	1.91165403	2.44940179	-2.08454331
C	2.31043300	3.04798521	1.21254506

H	2.24179786	4.15072703	1.22727979
H	3.36704836	2.75378374	1.26616896
H	1.78085066	2.64665240	2.08305179
O	0.35591568	2.86215802	-0.04376309
H	-0.10162013	1.96934269	-0.00861891
N	1.72477245	2.47831128	0.00008038
C	2.48713958	-0.61052522	0.04821918
H	3.09382593	-0.25519953	-0.79754854
C	1.16456369	-0.00391206	0.05086500
C	-0.06341495	0.03245925	0.05125986
C	-1.23950932	-0.84533657	0.04592833
C	-0.84996213	-2.33618695	-0.01249229
C	0.04975993	-2.80284460	1.11695320
C	1.46570799	-2.73644789	1.08685333
C	2.27212765	-2.15269701	-0.05952440
H	3.03458696	-0.36418940	0.97065915
F	-2.05522028	-0.57455327	-1.05045748
F	-2.03139645	-0.61608866	1.16473952
H	-1.66303710	-3.40164749	2.26961406
H	-0.36433675	-4.23455730	4.20439144
H	3.26166195	-2.63169351	-0.08019443
H	1.79425482	-2.36255455	-1.02658816
C	2.18204746	-3.22535754	2.19063962
C	1.54655827	-3.75918667	3.31012248
C	0.15418680	-3.81647322	3.34160111
C	-0.57392274	-3.34801886	2.25001624
H	3.27313381	-3.19093538	2.16184299
H	2.13639785	-4.13330919	4.14708788
H	-1.78894481	-2.90690917	-0.00032533
H	-0.38329978	-2.49032518	-0.99400164

DCl-*p*-MOBO (TS)
E = –4636.85
H = –4453.97
G = –4495.65
N_{imag} = 1, ν = –28.0227 i cm^{-1}

C	2.48571557	-0.47396875	0.01632113
H	3.10621011	-0.12623504	-0.82333645
C	1.16099820	0.13327707	-0.00788807
C	-0.07220202	0.11659071	-0.02203564
C	-1.22738772	-0.75843280	-0.01378036
C	-0.83113045	-2.25834292	-0.01685685
C	0.07914568	-2.69405980	1.11743557
C	1.49314272	-2.59787580	1.08165128
C	2.28448698	-2.01705209	-0.07605208
H	3.01938424	-0.21588855	0.94404414

Cl	-2.25015120	-0.46509991	-1.51680476
Cl	-2.28255916	-0.39121167	1.43997157
H	-1.61504392	-3.34326624	2.27021688
H	-0.29219365	-4.14165575	4.20311866
H	3.27700935	-2.48975086	-0.10099301
H	1.79981467	-2.23885357	-1.03694705
C	2.22370407	-3.06483564	2.18576394
C	1.60373122	-3.60985785	3.30841276
C	0.21362651	-3.70916764	3.33990990
C	-0.52824231	-3.25900757	2.25012077
H	3.31360681	-3.00463315	2.15409030
H	2.20442558	-3.96496962	4.14601111
H	-1.75665910	-2.84790139	0.00302261
H	-0.35491948	-2.43140951	-0.99148856
C	2.28021603	3.03079249	-1.17310602
H	3.33597937	2.72908425	-1.17005623
H	2.22074807	4.13235879	-1.21878579
H	1.78500943	2.60973977	-2.05427789
C	2.22667026	2.98583367	1.27339677
H	2.16301948	4.08486942	1.35708510
H	3.28192174	2.68407099	1.30518374
H	1.69448688	2.53154277	2.11557681
O	0.27288580	2.87209257	0.00345058
H	-0.17478235	1.96142717	-0.01140064
N	1.63413219	2.50192240	0.02709899

DCF$_3$-*p*-MOBO (TS)
E = –5519.74
H = –5317.20
G = –5364.81
N_{imag} = 1, ν = – 62.2081 i cm^{-1}

C	2.44298147	-0.38718853	-0.00534644
H	3.04357394	-0.03260760	-0.85763225
C	1.12266209	0.24435217	0.01181408
C	-0.12133851	0.22778349	0.01936972
C	-1.26953955	-0.70968598	0.00137184
C	-0.81622304	-2.21809877	0.03643742
C	0.11805446	-2.64543952	1.15323892
C	1.52681302	-2.53021640	1.07683574
C	2.26278045	-1.92671551	-0.10330646
H	3.00466908	-0.13825819	0.90900991
C	-2.05345485	-0.50214698	-1.33942162
C	-2.20469343	-0.38418314	1.21284536
H	-1.52891031	-3.36780387	2.33522143
H	-0.13591727	-4.18520100	4.20519325
H	3.26123565	-2.38275096	-0.16924281

H	1.74707073	-2.15201301	-1.04718060
C	2.29976382	-3.00674126	2.14741274
C	1.72314095	-3.58870599	3.27430468
C	0.33645291	-3.71861296	3.34080150
C	-0.44525852	-3.25587414	2.28508119
H	3.38707393	-2.92829902	2.08288077
H	2.35379976	-3.95181451	4.08599732
H	-1.71732264	-2.84350936	0.06935054
H	-0.33777359	-2.39985940	-0.93299308
C	2.21521399	2.92298370	-1.10563016
H	3.24673315	2.54851888	-1.12980246
H	2.23011404	4.02499274	-1.12761840
H	1.66948595	2.55312106	-1.97933980
C	2.20787916	2.81412150	1.35197797
H	2.22042721	3.90977869	1.47204343
H	3.23976732	2.43997278	1.34825494
H	1.65836093	2.36623250	2.18582280
O	0.22735904	2.84721664	0.11573018
H	-0.25607259	1.91880071	0.07929520
N	1.55230018	2.42530198	0.10142817
F	-1.25999458	-0.82531210	-2.39900429
F	-2.45983459	0.77071306	-1.52973487
F	-3.15339902	-1.29799178	-1.41340007
F	-1.49645450	-0.37022723	2.36753787
F	-3.19213663	-1.31059774	1.36035162
F	-2.80585179	0.82377216	1.10123787

DF-O-MOBO (TS)
E = –4504.99
H = –4336.54
G = –4375.21
N_{imag} = 1, ν = –131.5332 i cm^{-1}

C	2.20226885	2.57623958	-1.46242560
H	3.21982362	2.16993462	-1.41295506
H	2.23855592	3.63344590	-1.76867512
H	1.61502869	2.00905945	-2.19019134
C	2.22577245	3.18827548	0.92718208
H	2.11724448	4.27012342	0.75465568
H	3.29138624	2.93369700	0.95160161
H	1.75390276	2.92459634	1.87860635
O	0.23902124	2.78598267	-0.20462495
H	-0.21566036	1.82149928	0.04029104
N	1.56563415	2.43499083	-0.14785543
C	0.08241227	-2.52334328	-0.29359238
C	1.18315003	-2.06679522	-1.29442194
C	-0.00435325	0.32717043	0.32768798

C	2.46421790	-1.59285306	-0.65449386
C	1.25405009	0.31110022	0.26518692
C	2.49094150	-0.40094114	0.12510077
H	0.46599889	-2.56448256	0.73463890
H	1.41546076	-2.89931017	-1.97177079
O	-1.11491030	-1.71646634	-0.33440721
H	-0.27512055	-3.52342993	-0.56179429
H	0.74381665	-1.26905978	-1.91132511
C	-1.13081070	-0.61044907	0.49443976
C	3.68987339	-0.01568753	0.75313854
C	4.84920969	-0.77292566	0.61099762
C	4.83053864	-1.93193277	-0.16751646
C	3.64453681	-2.33220620	-0.78503801
H	3.69813147	0.87496617	1.37764618
H	5.76535591	-0.46222154	1.11296624
H	5.73498404	-2.52819677	-0.28746640
H	3.62897371	-3.24487686	-1.38329331
F	-1.18484605	-1.02425145	1.83277858
F	-2.33976566	-0.01325383	0.24882906

DIBBO (TS)

E = –5150.71

H = –4960.46

G = –5001.70

N_{imag} = 1, ν = –108.6838 i cm^{-1}

C	2.29226623	2.62334309	-1.26889207
H	3.25919055	2.11150434	-1.36873889
H	2.44720477	3.71285940	-1.33674255
H	1.62952453	2.29824772	-2.07629811
C	2.46823199	2.59224713	1.18353709
H	2.71678304	3.66641451	1.22172705
H	3.41664235	2.02462997	1.16307713
H	1.90351679	2.30981844	2.07638107
O	0.39133346	2.73759584	0.09905777
H	-0.13444036	1.80966014	0.08664602
N	1.68256018	2.24853610	0.00586763
C	0.08431933	-2.66644672	0.57239822
C	1.11181361	-2.62042031	-0.61691287
C	-0.11149820	0.16398115	0.04357344
C	2.43127552	-1.96746554	-0.26993704
C	1.14741940	0.10964347	0.05119827
C	2.39653453	-0.54800451	0.03947381
H	0.52582010	-2.14521453	1.43337837
H	-2.31027595	-3.91149786	0.41895414
C	-1.26365355	-2.04725719	0.27633055
H	-0.07009397	-3.70961149	0.87753940

H	-4.55003142	-3.04644759	0.00746607
C	-1.30627242	-0.60896966	0.05812699
B	3.77539555	-0.22137277	0.32811877
C	5.08793640	-0.79086933	0.40794616
C	4.88149731	-2.15599341	0.07245433
C	3.62609467	-2.70310247	-0.24126325
H	1.30610454	-3.64058373	-0.97291236
H	6.07560097	-0.41524134	0.64920790
H	5.75386256	-2.81472578	0.05899735
H	3.57488203	-3.76386945	-0.48722592
B	-2.71509470	-0.34456867	-0.13997101
C	-4.00113154	-0.97116689	-0.22146507
C	-3.71399036	-2.34174320	0.00751842
C	-2.42070842	-2.84161125	0.24121604
H	0.65502144	-2.06991200	-1.45146551
H	-5.01380868	-0.62783058	-0.39801432

DITO (TS)

E = –4531.25

H = –4363.89

G = –4403.50

$\boldsymbol{N}_{\mathrm{imag}}$ = 1, ν = –73.0722 i cm^{-1}

C	2.36936472	2.63413110	-1.24401521
H	3.39745988	2.25428094	-1.20011208
H	2.38481869	3.72614440	-1.39400232
H	1.84311727	2.16106928	-2.07857939
C	2.28461869	2.84531456	1.20888615
H	2.25217720	3.94697185	1.18929562
H	3.32739500	2.51256620	1.27743070
H	1.72800149	2.48161824	2.07805503
O	0.34996668	2.68335460	-0.07416667
H	-0.12813878	1.74383882	-0.01956638
N	1.67712206	2.28243487	-0.00105484
C	0.07033397	-2.91175004	0.48644700
C	1.13807420	-2.77031662	-0.67109269
C	-0.04473243	0.10224027	0.11363504
C	2.39601867	-2.03978960	-0.29344806
C	1.21761452	0.07422604	0.13655427
C	2.40187702	-0.67952714	0.04578916
H	0.55016784	-2.61465732	1.42947558
S	4.02078240	-0.14998338	0.44089400
C	-1.19131725	-2.11378320	0.30350379
H	-0.20070394	-3.97019263	0.59522663
C	4.66993152	-1.72110498	0.15859891
C	-1.19120909	-0.71916817	0.17383446
C	3.69277062	-2.61398773	-0.21115040

H	1.41066752	-3.77202569	-1.02806603
H	0.66026323	-2.25262121	-1.51420115
S	-2.82568964	-0.11368198	0.01606320
C	-3.48531992	-1.69844103	0.10852589
C	-2.50321539	-2.65292731	0.25305975
H	-4.55963759	-1.84468666	0.05995727
H	-2.71939430	-3.71729997	0.33506952
H	3.89956185	-3.65883050	-0.43837390
H	5.73592347	-1.89181583	0.26781648

F-COD (TS)
E = –3553.52
H = –3414.56
G = –3450.21
$\boldsymbol{N}_{\mathrm{imag}}$ = 1, ν = –92.5224 i cm^{-1}

C	2.23195205	2.83932073	-1.23477447
H	3.25103576	2.43204690	-1.23790076
H	2.27765438	3.93567276	-1.34225527
H	1.67073246	2.41625533	-2.07345039
C	2.22729414	2.93239263	1.22451139
H	2.26190976	4.03396972	1.25440582
H	3.25196009	2.53986848	1.25492457
H	1.67014099	2.56368847	2.09115343
O	0.24202236	2.87033588	-0.00611672
H	-0.23652510	1.92870107	0.02104752
N	1.56588766	2.45335983	0.01012737
C	1.21995651	-2.50856212	-0.64586999
C	0.15099784	-2.65563602	0.52644759
C	1.13265055	0.28379980	0.09235519
C	-1.12161555	-1.90535905	0.31866132
C	-0.12825502	0.26647871	0.09069565
C	-1.27633845	-0.57305286	0.14662199
H	0.71174637	-2.07663313	-1.51737547
H	0.62112800	-2.32048680	1.45969783
C	2.40349831	-1.66972730	-0.30733951
H	1.57870935	-3.50482258	-0.92938996
H	-0.10222062	-3.71519920	0.64871859
C	2.39149208	-0.35529038	0.01383700
F	3.58908819	-2.34963397	-0.25635154
F	-2.23214198	-2.70522364	0.27199697
H	-2.28449405	-0.17506663	0.02100484
H	3.32821886	0.14712107	0.25597297

CF_3-COD (TS)
E = –4355.56

H = –4197.38
G = –4242.41
N_{imag} = 1, ν = –36.8323 i cm^{-1}

C	2.31907072	2.91567631	-1.19204119
H	3.35359611	2.54964992	-1.17185791
H	2.32652750	4.01651747	-1.26325180
H	1.80692527	2.50191625	-2.06650279
C	2.23892857	2.93022429	1.26031991
H	2.24165107	4.03180391	1.31925600
H	3.27322693	2.56619872	1.31117719
H	1.67127116	2.52611400	2.10453197
O	0.29254216	2.88366029	-0.03001059
H	-0.18989859	1.98292140	-0.04372625
N	1.63046467	2.45911805	0.01578518
C	1.16208974	-2.60797339	-0.43717245
C	0.07899606	-2.57636351	0.73419578
C	1.11055267	0.17965810	0.00343318
C	-1.22436445	-1.92837806	0.36451815
C	-0.13997470	0.17561862	-0.01021960
C	-1.32043027	-0.61196186	0.03641874
H	0.68711267	-2.21352294	-1.34420393
H	0.51113890	-2.02413857	1.57796659
C	2.40232468	-1.80318728	-0.16963941
H	1.42858575	-3.65052805	-0.64089581
H	-0.09560878	-3.60188212	1.07667577
C	2.37114119	-0.45408891	0.00080549
C	3.70055320	-2.55125979	-0.05999973
C	-2.44246942	-2.80934290	0.34593418
H	-2.29124512	-0.17730970	-0.20019270
H	3.28975600	0.10572744	0.16741740
F	-2.27711592	-3.85420638	-0.52642414
F	-3.57841837	-2.16227621	-0.01822484
F	-2.67971332	-3.37166376	1.57163270
F	3.98223377	-3.24485470	-1.20529414
F	3.65101075	-3.47984127	0.94606404
F	4.76985958	-1.75016978	0.18945581
